# Thiazole Amides, A Novel Class of Algaecides against Freshwater Harmful Algae

**DOI:** 10.1038/s41598-018-26911-6

**Published:** 2018-06-04

**Authors:** Ying Wang, Qisheng Liu, Zhigang Wei, Na Liu, Yajuan Li, Duo Li, Zhong Jin, Xiaohua Xu

**Affiliations:** 1 State Key Laboratory and Institute of Elementoorganic Chemistry, College of Chemistry, Nankai University, Tianjin, 300071 P. R. China; 2 Collaborative Innovation Center of Chemical Science and Engineering (Tianjin), Tianjin, 300071 P. R. China

## Abstract

Currently, harmful algal blooms are being one of ever-increasing global environmental problems. Much attention has been paid to the use of natural products as the selective algaecides due to their low toxicity, high selectivity and eco-friendly properties. In the present study, the thiazole alkaloid (**1**), originally isolated from *Thermoactino-myces* strain TM-64, was shown to exhibit potent algicidal activity against three typically harmful cyanobacterial algae, *S*. *obliqnus*, *M*. *aeruginosa*, and *C*. *pyrenoidosa*. Based on our previous work, a practical, scalable synthesis of alkaloid (**1**) was developed and reaction could be readily scaled up to more than 100 g. In addition, twenty-six analogues of alkaloid (**1**) by replacement of tryptamine moiety with different aromatic and aliphatic amines were also prepared. The bioassay results showed that most of these derivatives displayed potent algicidal activity against three harmful algae *S*. *obliqnus*, *M*. *aeruginosa*, and *C*. *pyrenoidosa* with IC_50_ values in the range of 1.5–5.0 μg/mL. Amongst them, compounds (**10**) and its hydrochloric salt (**10S**) were found to reveal powerful growth inhibitory activity against harmful cyanobacterial algae with IC_50_ values as low as 0.08 μg/mL, comparable to those of commercial algicide CuSO_4_ and herbicide Diuron.

## Introduction

Over the past decades, harmful algal blooms (HABs) have been becoming an increasing global environmental problem. Virtually, they do not only cause seriously public health problems, but also substantially economic losses^1–3^. To control HABs efficiently, extensive strategies including physical methods (*e*.*g*. clays and flocculants)^4–6^, chemical methods (*e*.*g*. copper sulfate, surfactants, sodium hypochlorite, and herbicides such as Diuron, Endothal, Atrazine, etc.)^7–10^, and biological methods (*e*.*g*. algicidal bacteria, algicidal viruses and plankton grazers)^11–13^ have been developed to date. Despite this significant progress, unfortunately, all these strategies are shown to be either too expensive to implement, or nonspecific to harmful algae. As a result, development of novel selective and eco-friendly algicidal agents against harmful algae is highly desirable.

Recently, the effectiveness of natural products (NPs) as the powerful algicidal agents against marine and freshwater harmful algae has received more and more interests from academic and industrial communities due to their intrinsically low toxicity, high selectivity and eco-friendly properties. Schrader and co-workers discovered that 9,10-anthraquinones, produced or released during lignin decomposition act via photosynthetic electron transport, display selectively algicidal activity against cyanobacterium *Oscillatoria perornata*^14–17^. Amongst them, anthraquinone-59 was shown to have the highest algicidal activity against musty-odor *O*. *perornata* (LC_50_ = 2.06 mg/L). The cyanobacterial alkaloid nostocarboline was also found by Gademann and co-workers to reveal potent algicidal activity with IC_50_ values of 2.1 μM against *Microcystis aeruginosa*, 5.8 μM against *Synechococcus obliqnus*, and 29.1 μM against *Kirchneriella contorta*, respectively^18,19^. Additionally, Mizuno *et al*. reported that natural stilbene analogues exhibited fully inhibitory activity against *O*. *perorata* at a concentration as low as 10 μM^20^.

**Figure 1:**
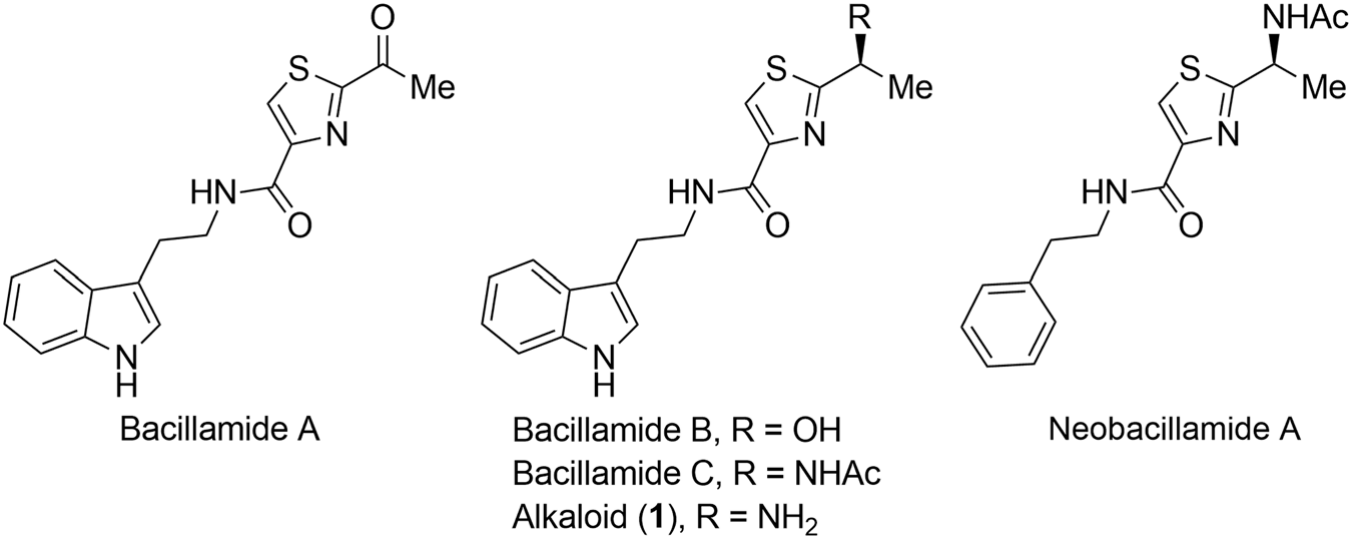
Bacillamides Alkaloids.

On the other hand, the use of algicidal bacteria turns out to be a versatile alternative approach to control the bloom of harmful algae in marine and freshwater environments^12,21,22^. Generally, in addition to killing algal cells directly by contact action, bacteria attack algae by releasing secondary metabolites to inhibit the growth of harmful algae^23–27^, namely allelopathy^28^. These natural secondary metabolites, termed alleochemicals, show extremely low toxicity to mammalian animal, high selectivity, readily degradable, and environment-friendly properties. To date, several types of natural secondary metabolites with algicidal activity including pigments^12,21,22^, diketopiperazines^29–31^, norharmalane^32^, and proteins^33,34^, have been isolated and identified from bacteria. The thiazole alkaloids (Fig. 1), *N*-(2-(1*H*-indol-3-yl)ethyl)-2-acetylthiazole-4-carboxamide (alkaloid (**1**)), was originally isolated from *Thermoactino-myces* strain TM-64 in 1976^35^. As the congeners of this class of alkaloids, bacillamides A–C, were recently isolated from marine *Bacillus endophyticus* collected during termination of a toxic algal bloom^36,37^. It was found that bacillamide A show potent antibiotic activity against dinoflagellates and raphidophytes. All these alkaloids highlight a dipeptide skeleton incorporating a central thiazole motif and a *C*-terminus tryptamide. As an only exception, neobacillamide A, firstly isolated from marine bacterium *B*. *vallismortis* C89^38^, has a unique *C*-terminus phenethylamide unit in its molecular scaffold. Due to their limited availability from natural source, the algicidal activity of this class of secondary metabolites has not been explored.

Nowadays, cyanobacterial blooms are becoming a worldwide aquatic environmental problem, which is tightly coupled with increasing water eutrophication and climate change^39,40^. In particular, *M*. *aeruginosa* and *S*. *obliqnus* were shown to be the most frequently reported HABs species in the freshwater bodies. Therefore, controlling cyanobacteria blooms is becoming one of the most important aspects in the comprehensive management of water source. As parts of our ongoing research on natural alleochemicals, we herein report the algicidal potential of bacillamide alkaloids against three typically harmful cyanobacterial algae, *S*. *obliqnus*, *M*. *aeruginosa*, and *Chlorella pyrenoidosa*. In addition, a mini synthetic library consisting of twenty-six analogues of bacillamide alkaloids were also prepared and evaluated for their algicidal activity. Meanwhile, considering the low solubility of these compounds in water, their water-soluble hydrochloric salts were also subject to evaluation for selective algicidal activity against harmful freshwater algae.

## Methods

### Materials

Melting point are determined using an X-4 digital melting point apparatus and thermometer uncorrected. ^1^H and ^13^C nuclear magnetic resonanance (NMR) spectra are recorded in CDCl_3_ or *d*_6_-DMSO solution on a BRUKER (400 MHz) instrument (Rheinstetten, Germany) using tetramethylsilane as an internal standard, and chemical shift values (*δ*) are reported in parts per million. High resolution mass spectrometer (HRMS) data are obtained on a high resolution ESI-FTICR mass spectrometry (Ionspec 7.0 T). All reagents are commercially available and purified according to the standard methods prior to use.

### Chemistry

**Figure 2:**
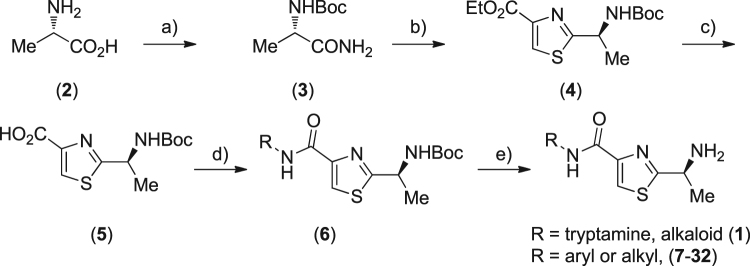
Synthesis of alkaloid (**1**) and its analogues. Reagents and conditions (**a**) (i) aq. NaOH, 0 °C, (Boc)_2_O, then rt 8 h, (ii) (Boc)_2_O, pyridine, NH_4_HCO_3_, rt 12 h, overall yield 92% for two steps (**b**) Na_2_SO_4_, DME, P_2_S_5_, ethyl 3-bromopyruvate, 85% (**c**) LiOH, THF/MeOH/H_2_O, 87% (**d**) (i) *iso*-butyl chloroformate, NMM, CH_2_Cl_2_, (ii) tryptamine, NMM, CH_2_Cl_2_ (**e**) 3 N HCl, ethyl acetate. DME: 1,2-dimethoxyethane, THF: tetrahydrofuran, NMM: *N*-methylmorholine.

The thiazole alkaloid (**1**) was synthesized using L-alanine (**2**) as the starting material according to a slightly modified procedure of our previous work (Fig. 2)^41^. In a similar manner, twenty-six analogues (**7**–**32**) of alkaloid (**1**) in which tryptamine moiety was taken place by substituted aromatic and aliphatic amines were also prepared.

#### (S)-tert-Butyl (1-amino-1-oxopropan-2-yl)carbamate (**3**)

In a 2.5 L three-neck flask equipped with mechanical agitator, (Boc)_2_O (314 g, 1.44 mol, 1.2 equiv) was added slowly to a cooled (0 °C) and stirred solution of L-alanine (**2**) (108 g, 0.12 mol) in 2 M aqueous NaOH solution (600 mL). The reaction mixture was warmed to room temperature gradually over 2 h and stirred at room temperature until the starting materials was consumed completely, and at that time the pH value of the solution was adjusted to 2 with concentrated hydrochloric acid. The mixture was extracted with EtOAc (600 mL × 3) and the combined organic layers were dried over anhydrous sodium sulfate. After removal of solvent in vacuum, the resulted residue was redissolved in 1,4-dioxane (1200 mL). To this solution was added NH_4_HCO_3_ (108 g, 1.37 mol), pyridine (55 mL) and (Boc)_2_O (336 g, 1.5 mol) in turn. The reaction mixture was then stirred at room temperature for another 4 h. The solvent was removed under reduced pressure and water (1000 mL) was then added. The mass was re-extracted with EtOAc (600 mL × 3) and the combined organic layers were dried over anhydrous Na_2_SO_4_ followed by removal of solvent in vacuum. The obtained residue was triturated with acetone (100 mL) to give the pure amide (**3**) as a white solid (211 g) in 92% yield^41^, m. p. 120–122 °C, ^1^H NMR (CDCl_3_, 300 MHz) *δ*: 7.77 (s, 1H), 7.46 (s, 1H), 5.19 (s, 1H), 4.53 (m, 1H), 1.52 (d, *J* = 6.4Hz, 3H), 1.48 (s, 9H).

#### (S)-Ethyl 2-(1-((tert-butoxycarbonyl)amino)ethyl)thiazole-4-carboxylate (**4**)

In a 2 L three-neck flask equipped with mechanical agitator, P_2_S_5_ (71 g, 0.5 mol), Ethyl bromopyruvate (117 g, 0.6 mol), and anhydrous Na_2_SO_4_ (284 g) was added slowly to a cooled (0 °C) and stirred solution of (*S*)-*tert*-butyl (1-amino -1-oxopropan-2-yl)-carbamate (**3**) (102 g, 0.5 mol) in acetone (500 mL) under Ar_2_. The reaction mixture was stirred at room temperature overnight and the solid substances were then filtered off from the solution. After removal of the solvent under reduced pressure, the obtained residue was purified by flash column chromatography (petroleum ether/ethyl acetate = 2: 1, v/v) on silica gel to give compound (**4**) as a yellow oil liquid in 85% yield (127 g).^45 1^H NMR (CDCl_3_, 400 MHz) δ: 8.10 (s, 1H), 5.31 (s, 1H), 5.13 (s, 1H), 4.43 (q, *J* = 7.2 Hz, 2H), 1.64 (d, *J = *6.4 Hz, 3H), 1.46 (s, 9H), 1.41 (t, *J = *7.1 Hz, 3H).

#### (S)-2-(1-((tert-Butoxycarbonyl)-amino)-ethyl)thiazole-4-carboxylic acid (**5**)

In a 250 mL three-necked flask equipped with mechanical agitator, the ester (**4**) (11.2 g, 0.037 mol) was dissolved in a solution of THF/MeOH/H_2_O (40 mL, v: v: v = 4: 2: 1), and then LiOH (1.8 g, 0.074 mol) was added in one portion. The reaction mixture was stirred at room temperature for another 2 h and the solid substances were filtered off from the solution. The filtrate was concentrated under reduced pressure and neutralized to pH 7 with diluted hydrochloric acid. The mixture was extracted with ethyl acetate (100 mL × 3) and the combined organic phase was dried over anhydrous Na_2_SO_4_ followed by removal of the solvent in vacuum. The obtained residue was purified by flash column chromatography (petroleum ether/ethyl acetate = 2: 1, v/v) on silica gel to give compound (**5**) as a white solid (8.8 g) in 87% yield, m. p. 101–103 °C^41^. ^1^H NMR (CDCl_3_, 400 MHz) δ: 8.05 (s, 1H), 4.45 (q, *J = *6.6 Hz, 1H), 4.36 (q, *J = *7.1 Hz, 2H), 1.95 (s, 2H), 1.50 (d, *J* = 6.6 Hz, 3H), 1.35 (t, *J = *7.1 Hz, 3H).

#### N-(2-(1H-Indol-3-yl)ethyl)-2-acetylthiazole-4-carboxamide (alkaloid (**1**))

To a solution of acid (**5**) (680 mg, 2.5 mmol) in dry dichloromethane (25 mL) was added *iso*-butyl chloroformate (340 mg, 2.5 mmol) and *N*-methylmorpholine (NMM) (250 mg, 2.5 mmol). The resulted mixture was vigorously stirred at room temperature for 1 h. Tryptamine (440 mg, 2.5 mmol) and another part of NMM (250 mg, 2.5 mmol) in dichloromethane (10 mL) was added to the aforementioned solution and the reaction mixture was stirred for another 2 h. Water (10 mL) was added and organic phase separated off dried over anhydrous MgSO_4_. The solvent was evaporated under reduced pressure to give a crude product (**6**), which was used directly in the next step reaction without further purification.

In 25 ml sealing tube, the crude product (**6**) (1 mmol) was dissolved in EtOAc (5 mL), then H_2_O (1 mL) and concentrated hydrochloric acid (1 mL) was added. The reaction mixture was stirred at room temperature for 12 h until the reaction is finished monitored by TLC. The reaction solution was neutralized to pH 8–9 with saturated K_2_CO_3_ solution. The organic phase was separated off and aqueous phase was extracted with with EtOAc (2 × 20 mL). The combined organic layer was dried over anhydrous sodium sulfate followed by removal of the solvent under reduced pressure. The obtained residue was subjected to purification by flash column chromatography (petroleum ether/ethyl acetate = 2: 1, v/v) on silica gel to give alkaloid (**1**) as a light brown oil (252 mg) in 80% yield. [α]^D^_20_=+ 0.015 (*c* = 1.0, EtOH). ^1^H NMR (400 MHz, CDCl_3_) *δ*: 1.49 (d, *J = *6.7 Hz, 3H), 1.76 (s, 2H), 3.09 (t, *J = *3.0 Hz, 2H), 3.78 (q, *J = *6.8 Hz, 2H), 4.28 (q, *J* = 6.7 Hz, 1H), 7.03 (d, *J = *2.0 Hz, 1H), 7.12 (t,*J = *7.3 Hz,1H), 7.19 (t, *J* = 7.3 Hz, 1H), 7.36 (d, *J* = 8.1 Hz,1H), 7.55 (t, *J = *5.5 Hz,1H), 7.66 (d, *J* = 7.8 Hz, 1H), 7.99 (s, 1H), 8.77 (s, 1H); ^13^C NMR (CDCl_3_, 100 MHz) δ: 24.7, 25.5, 39.9, 49.6, 111.4, 112.7, 118.8, 119.3, 122.0, 122.3, 122.7, 127.4, 136.6, 149.9, 161.4, 178.5. HRMS(ESI-TOF) calcd. for C_16_H_19_N_4_OS^+^ [M+H]^+^ 315.1274, found: 315.1281.

Following the similar procedure, amides (**7**–**32**) were prepared readily in 80–94% overall yields from the carboxylic acid (**5**) through using substituted aromatic or aliphatic amines as the starting materials (See the Supporting Information for the detailed characterized data).

### Biological studies

According to the method established by Schrader *et al*.^42^, algicidal activity of alkaloid (**1**) and its amide analogues (**7**–**32**), as well as their hydrochloric salts (**7S**–**27S**), was evaluated against three typical Cyanobacterial algae, *S*. *obliqnus*, *M*. *aeruginosa*, *and C*. *Pyrenoidosa* at different concentrations. After three algae species were inoculated, the variation of algae density over 7 days were recorded continuously. It was found that the best period of inoculation is 4th day in the growth cycle of three algae species. The wavelength at 650 nm was determined as ideal incident wavelength for the measurement of algae density. Three Cyanobacterial algae *S*. *obliqnus*, *M*. *aeruginosa*, and *C*. *pyrenoidosa* were offered by *Freshwater algae culture collection of the institute of Hydrobiology* (FACHB-collection). Each culture for three species was maintained separately in continuous, steady-state growth microplates, which were placed in a growth chamber held at 28 ± 1 °C and illuminated continuously under fluorescent lights at a photon flux density of 28 μE·m^−1^s^−1^. Absorbance values of each wells were measured by a Packard model Spectra Count microplate photometer.

Alkaloid (**1**) and its analogues (**7**–**32**), as well as their hydrochloric salts (**7S**–**27S**), were dissolved separately in DMSO to provide stock solution concentrations. Initially, each stock solution was aseptically pipetted into the bottom of separate wells (20 μL per well) in a 96-well microplate and the final test concentrations for each sample were 0, 0.78, 1.56, 3.12, 6.25, 12.5, and 25 μg/mL. Wells of control samples contained only unialgal culture. The solution of CuSO_4_ and Diuron at the same concentration were chose as the reference sample. Each experiment was performed at least three times. Through measuring the area values under the individual growth curves, the inhibitory rate (%) for the individual tested concentration was calculated according to the following equation (1):

1 $${I}_{i}=({A}_{c}-{A}_{i})/{A}_{i}\times 100 \% $$

- *I*_*i*_: inhibitory rate of biomass growth by toxicant concentration *i* (%)
- *A*_*c*_: average area under the growth curve by control sample
- *A*_*i*_: average area under the growth curve by test concentration *i*

Mean values of 96-hour IC_50_ were determined for each group of plates, and standard deviation were calculated and reported as the mean ± SD.

### Algicidal activity

The IC_50_ values of all compounds against three freshwater algae species were calculated using SPSS v19.0, and the algicidal activities of alkaloid (**1**) and its amide analogues (**7**–**32**), as well as the corresponding hydrochloric salts (**7S**–**27S**) are listed in Table 1 and 2, respectively.

**Table 1:** Algicidal activity of alkaloid (1) and the amide analogues (**7–32**) against three freshwater algae.

Entry	R	IC_50_ value (μg/mL)		
		*S*. *obliqnus*	*M*. *aeruginosa*	*C*. *pyrenoidosa*
1	Alkaloid (**1**)	14.33 ± 1.15	114.60 ± 3.35	381.57 ± 5.16
2	C_6_H_5_ (**7**)	0.45 ± 0.09	11.16 ± 1.12	4.61 ± 0.45
3	4-F-C_6_H_4_ (**8**)	2.74 ± 0.11	4.51 ± 0.92	3.57 ± 0.37
4	4-Cl-C_6_H_4_ (**9**)	4.12 ± 0.13	2.19 ± 0.09	1.37 ± 0.12
5	4-Br-C_6_H_4_ (**10**)	0.08 ± 0.03	1.51 ± 0.11	1.52 ± 0.05
6	4-Me-C_6_H_4_ (**11**)	2.66 ± 0.18	6.16 ± 1.13	10.41 ± 1.12
7	4-OMe-C_6_H_4_ (**12**)	0.08 ± 0.02	2.35 ± 0.91	17.35 ± 0.92
8	4-CF_3_-C_6_H_4_ (**13**)	4.35 ± 0.17	2.36 ± 0.12	4.58 ± 0.23
9	3-Cl-C_6_H_4_ (**14**)	1.52 ± 0.12	5.19 ± 0.19	4.37 ± 0.14
10	3-Br-C_6_H_4_ (**15**)	0.66 ± 0.07	4.79 ± 0.15	5.77 ± 0.11
11	3-Me-C_6_H_4_ (**16**)	2.86 ± 0.14	24.16 ± 3.12	7.18 ± 0.05
12	3-CF_3_-C_6_H_4_ (**17**)	9.21 ± 1.12	12.19 ± 2.13	10.44 ± 0.99
13	2-F-C_6_H_4_ (**18**)	3.95 ± 0.92	2.55 ± 0.19	3.41 ± 0.14
14	2-Cl-C_6_H_4_ (**19**)	0.10 ± 0.08	12.23 ± 1.16	8.16 ± 1.34
15	2-Br-C_6_H_4_ (**20**)	3.31 ± 0.13	8.79 ± 1.23	2.84 ± 0.93
16	2-Me-C_6_H_4_ (**21**)	0.43 ± 0.02	10.05 ± 1.71	26.07 ± 3.25
17	2-OMe-C_6_H_4_ (**22**)	0.68 ± 0.06	3.12 ± 0.56	2.19 ± 0.71
18	4-OCF_3_-C_6_H_4_ (**23**)	5.93 ± 0.11	7.20 ± 0.71	3.80 ± 0.16
19	3,4-Cl_2_-C_6_H_3_ (**24**)	4.31 ± 0.21	9.81 ± 1.13	5.11 ± 1.01
20	3-Me,4-F-C_6_H_3_ (**25**)	1.81 ± 0.07	6.73 ± 1.21	5.10 ± 0.13
21	4-CO_2_Et-C_6_H_4_ (**26**)	29.53 ± 1.56	3.29 ± 0.21	4.16 ± 0.56
22	4-COMe-C_6_H_4_ (**27**)	4.73 ± 0.54	2.33 ± 0.32	2.84 ± 0.23
23	2-pydinyl (**28**)	3.97 ± 0.17	3.09 ± 0.15	14.76 ± 1.26
24	*t*-Bu (**29**)	6.23 ± 0.98	5.31 ± 0.45	3.46 ± 0.67
25	*n*-Bu (**30**)	5.23 ± 0.36	5.47 ± 0.41	4.65 ± 0.25
26	PhCH_2_CH_2_- (**31**)	5.98 ± 0.49	57.49 ± 6.26	2.84 ± 0.56
27	PhCH_2_- (**32**)	100.20 ± 0.34	9.48 ± 1.08	4.16 ± 0.88
28	CuSO_4_	0.99 ± 0.06	1.29 ± 0.02	1.56 ± 0.09
29	Diuron	0.79 ± 0.08	1.35 ± 0.15	1.42 ± 0.27

**Table 2:** Algicidal activity of the amide analogues in hydrochloric salt (**7S–27S**) form against three freshwater algae.

Entry	Hydrochloric salt (R)	IC_50_ value (μg/mL)		
		*S*. *obliqnus*	*M*. *aeruginosa*	*C*. *pyrenoidosa*
1	C_6_H_5_ (**7S**)	0.14 ± 0.06	1.33 ± 0.16	2.54 ± 0.28
2	4-F-C_6_H_4_ (**8S**)	1.14 ± 0.09	3.00 ± 0.12	2.69 ± 0.23
3	4-Cl-C_6_H_4_ (**9S**)	4.00 ± 0.34	1.18 ± 0.21	0.40 ± 0.10
4	4-Br-C_6_H_4_ (**10S**)	0.55 ± 0.16	0.36 ± 0.02	0.75 ± 0.07
5	4-Me-C_6_H_4_ (**11S**)	6.83 ± 0.45	4.34 ± 0.36	3.37 ± 0.28
6	4-OMe-C_6_H_4_ (**12S**)	3.16 ± 0.29	1.40 ± 0.18	2.88 ± 0.39
7	4-CF_3_-C_6_H_4_ (**13S**)	0.11 ± 0.02	0.43 ± 0.04	0.26 ± 0.02
8	3-Cl-C_6_H_4_ (**14S**)	2.01 ± 0.19	0.79 ± 0.08	0.66 ± 0.06
9	3-Br-C_6_H_4_ (**15S**)	1.59 ± 0.16	2.96 ± 0.26	2.75 ± 0.30
10	3-Me-C_6_H_4_ (**16S**)	4.07 ± 0.39	1.95 ± 028	1.82 ± 0.14
11	3-CF_3_-C_6_H_4_ (**17S**)	1.59 ± 0.23	3.09 ± 0.42	2.39 ± 0.24
12	2-F-C_6_H_4_ (**18S**)	1.12 ± 0.09	0.26 ± 0.01	0.27 ± 0.09
13	2-Cl-C_6_H_4_ (**19S**)	0.14 ± 0.02	3.07 ± 0.34	2.50 ± 0.16
14	2-Br-C_6_H_4_ (**20S**)	0.68 ± 0.06	1.23 ± 0.12	0.41 ± 0.04
15	2-Me-C_6_H_4_ (**21S**)	13.63 ± 1.20	4.93 ± 045	5.15 ± 0.56
16	2-OMe-C_6_H_4_ (**22S**)	1.74 ± 0.24	1.12 ± 0.16	0.35 ± 0.02
17	4-OCF_3_-C_6_H_4_ (**23S**)	4.68 ± 0.38	4.45 ± 0.58	0.66 ± 0.03
18	3,4-Cl_2_-C_6_H_3_ (**24S**)	5.59 ± 0.78	5.18 ± 0.66	3.42 ± 0.32
19	3-Me,4-F-C_6_H_3_ (**25S**)	16.62 ± 1.48	1.79 ± 0.22	1.59 ± 0.19
20	4-CO_2_Et-C_6_H_4_ (**26S**)	10.60 ± 1.18	2.46 ± 0.23	2.38 ± 0.32
21	4-COMe-C_6_H_4_ (**27S**)	0.75 ± 0.05	0.58 ± 0.04	0.42 ± 0.02
22	CuSO_4_	0.99 ± 0.06	1.29 ± 0.02	1.56 ± 0.09
23	Diuron	0.79 ± 0.08	1.35 ± 0.15	1.42 ± 0.27

### Preliminary toxicity tests on non-target organisms

Toxicity of this class of thiazole amides towards non-target organisms, such as higher plants and fishes, was evaluated preliminarily using compound (**10**) as a representative sample. Herbicidal activity of compound (**10**) against two terrestrial plants, *Brassica campestris* and *Echinochloa crusgalli*, and two aquatic plants, *Ceratophyllum demersum L* and *Hydrilla verticillata*, was evaluated at a concentration of 500 mg/L using a previously reported method^43^. In addition, toxicity of compound (**10**) toward fish *Barchydanio rerio var* was also tested at the concentrations of 200 mg/L and 100 mg/L, respectively. The preliminary results were reported in Table 3.

**Table 3:** Toxicity of compound (**10**) toward higher plants and fish.

Higher plant	*B. campestris*	*E. crusalli*	*C. demersum L*.	*H*. *verticillata*
Herbicidal activity (500 mg/L)	n.a.	n.a.	n.a.	n.a.
Fish	Barchydanio rerio var			
Dosage (mg/L)	100	200	control sample	
Survival ratio in 7 days (%)	100	100	100	

n.a.: no activity.

## Results and Discussion

### Synthesis

As illustrated in Fig. 2, alklaoid (**1**) and its amide analogues were prepared from L-alanine in five linear steps. Generally, the thiazole ester (**4**) is the key intermediate for synthesis of bacillamide-type alkaloid. To the best of our knowledge, although there are several approaches to synthesis this compound^44–49^, none of them are practical for scale-up preparation. In our previous work^41^, we developed a procedure through utilizing *N*-Boc-alanine as the starting materials. Firstly, it was converted into *N*-Boc-amino acid amide with excellent yield using the (Boc)_2_O-pyridine system. Subsequent thionation using Lawesson’s reagent afforded the desired *N*-Boc-amino acid thioamide, which was then reacted with ethyl 3-bromopyruvate to afford an intermediate followed by treatment with trifluoroacetic anhydride and 2,6-lutidine to give the thiazole ester (**4**). Unfortunately, careful purification by column chromatography is necessary for each step in the synthetic sequence. In present study, this synthetic route was modified for the convenience of scale-up synthesis. Therefore, a practical one-pot synthetic methodology for the key intermediate thiazole ester (**4**) was developed. Without Lawesson’s reagent, *N*-Boc-L-amino acid amide (**3**) directly reacted with ethyl 3-bromopyruvate in the presence of P_2_S_5_ to provide compound (**4**) in 85% yield, and isolation and purification of the related intermediate is not necessary. On the basis, reaction could be readily scaled up to more than 100 g.

The ester (**4**) was then successfully hydrolyzed to the carboxylic acid (**5**) in 87% yield using LiOH in a mixture solvents of THF/MeOH/H_2_O. Finally, treatment of acid (**5**) with *iso-*butyl chloroformate and subsequent tryptamine in the presence of NMM followed by the removal of the *N*-Boc protected group provided smoothly alkaloid (**1**) in an overall yield of 80%. In a similar manner, a series of the thiazole amide analogues (**7**–**32**) were prepared in 80–94% yields by coupling of the carboxylic acid (5) with substituted aromatic and aliphatic amines.

### Algicidal activity

As shown in Table 1, most of test samples displayed potent algicidal activity against three harmful algae *S*. *obliqnus*, *M*. *aeruginosa*, and *C*. *pyrenoidosa* with IC_50_ values in the range of 1.5–5.0 μg/mL. Compared to naturally occurring alkaloid (**1**)^50^, most of synthetic analogues exhibit obviously higher algicidal activity with IC_50_ values of less than 5.0 μg/mL. For free amine derivatives, compounds (**7**), (**10**), (**12**), (**15**), (**19**), (**21**), and (**22**) show excellent algicidal activity against *S*. *obliqnus* with IC_50_ values of less than 1.0 μg/mL. In particular, compounds (**10**) and (**12**) display highest algicidal activity against *S*.*obliqnus* with an IC_50_ value as low as 0.08 μg/mL, respectively, while natural alkaloid (**1**) only show algicidal activity with an IC_50_ value of 14.33 μg/mL under the same conditions. Notably, related to commercial algicide CuSO_4_ and herbicide Diuron, compound (**10**) displays comparably inhibitory potential toward all three harmful algae with IC_50_ values of 0.08, 1.51, and 1.52 μg/mL, respectively. From data outlined in Table 2, most water-soluble hydrochloric salt analogues show comparable algicidal activity to those of the corresponding free amines. Of the salts to be evaluated, compounds (**10S**), (**13S**), and (**27S**) appear to be the promising algicidal agents which show remarkable algicidal efficacy against all three algae species with IC_50_ value of less than 1.0 μg/mL.

In order to deduce the structure-activity relationship (SAR), several alkyl-substituted thiazole amide analogues (**29**–**32**) (Entries 24–27, Table 1) were thus prepared and evaluated for their algicidal activities. Except compound (**32**), almost all these compounds display obviously higher algicidal activity than alkaloid (**1**) under the experimental conditions, suggesting that it is thiazole amide motif, not tryptamine, that is the active pharmacophore for their algicidal activity. Generally, structural modification using substituted anilines instead of tryptamine further increases the algicidal activity of this class of thiazole amide derivatives. The different substitution modes in the phenyl ring of aniline also influence their algicidal activity greatly. For Cyanobacterial algae *S*. *obliqnus*, the thiazole amides derived from 4-bromo and 4-OMe substituted anilines, compounds (**10**) and (**12**) (Entries 5 and 7, Table 1), both exhibit highest algicidal activity with an IC_50_ value of 0.08 μg/mL more than 10 times higher than those of commercial algicide CuSO_4_ (IC_50_ 0.99 μg/mL) and herbicide Diuron (IC_50_ 0.79 μg/mL). In addition, compound (**10**) also exhibits comparable algicidal activity to the commercial control samples against algae *M*. *aeruginosa* (IC_50_ 1.51 μg/mL). While for algae *C*. *pyrenoidosa*, a 4-chloro or 4-bromo substituent group (Entries 4 and 5, Table 1) improves significantly the algicidal activity (IC_50_ 1.37 μg/mL for compound (**9**), 1.52 μg/mL for compound (**10**)). Generally, the thiazole amides in hydrochloric salt form show higher algicidal activity than the corresponding free amines against Cyanobacterial algae *M*. *aeruginosa*, and *C*. *pyrenoidosa*. For instance, the hydrochloric salts of compounds (**13**) (R = 4-trifluoromethylphenyl), (**20**) (R = 2-bromophenyl) and (**27**) (R = 4-acetylphenyl) reveal the obviously enhanced algicidal activity against all three Cyanobacterial algae than the relative free amines (Entries 7, 14 and 21, Table 2). It is noteworthy that both free amine (**10**) and its hydrochloric acid salt (**10S**) show a comparable algicidal activity relative to commercial algicide CuSO_4_ and herbicide Diuron.

**Figure 3:**
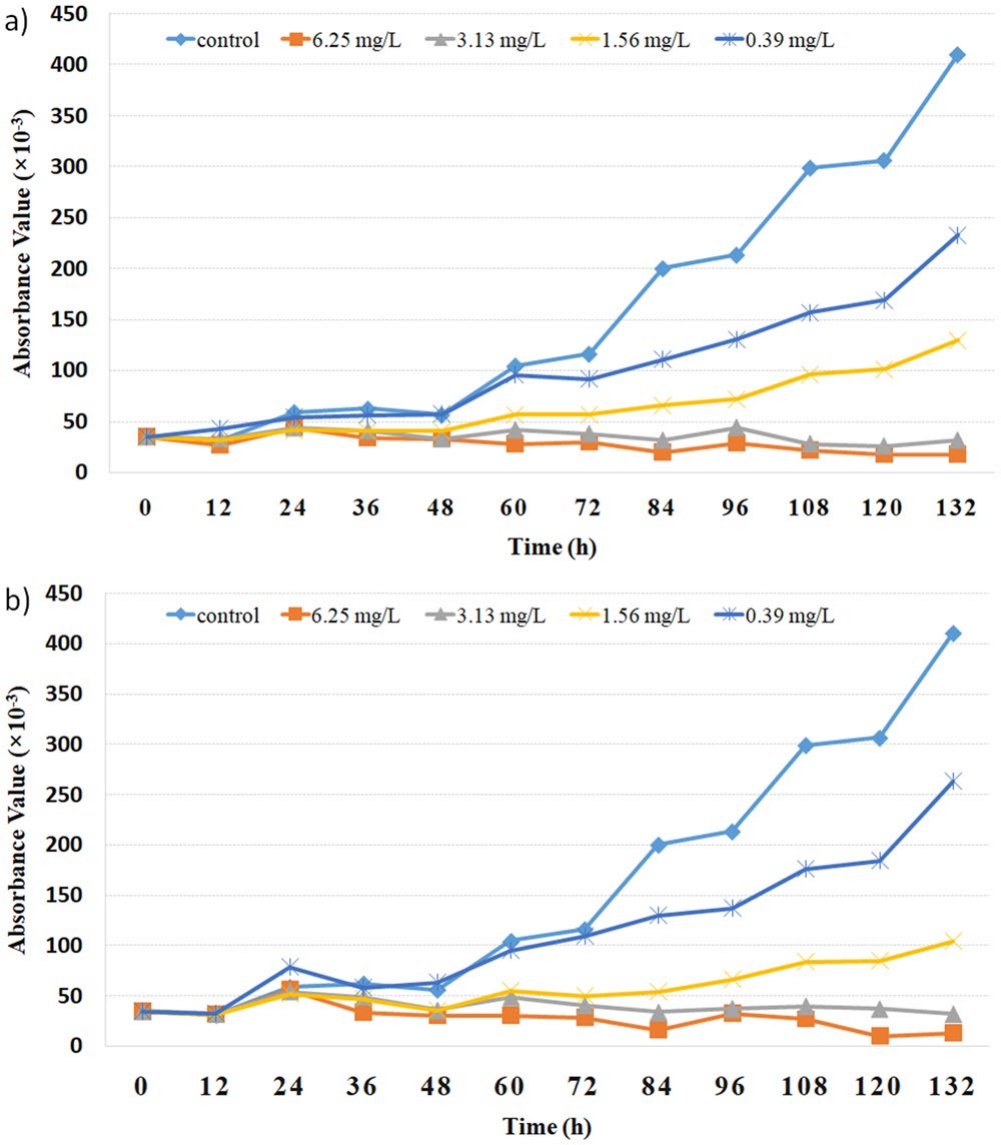
Variation of absorbance values with the increment of time at the different concentration (**a**) for thiazole amide (10) (**b**) for hydrochloric salt (10S).

**Figure 4:**
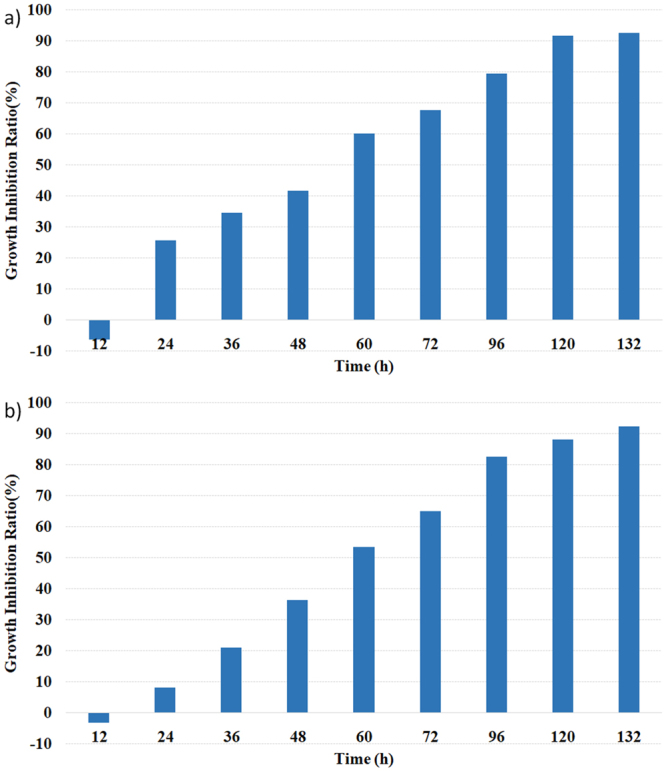
Variation of growth inhibitory ratios with the increment of time at the lowest complete inhibitory concentration (3.13 μg/mL) (**a**) for thiazole amide (**10**) (**b**) for hydrochloric salt (**10S**).

Currently, *M*. *aeruginosa* appears to be the most harmful algae existing in the freshwater bodies in the developing countries. To investigate growth inhibitory potential of this class of thiazole amides on *M*. *aeruginosa*, thiazole amide (**10**) and its hydrochloric acid salt (**10S**) were subject to further survey for the optimal inhibition time and concentration (Figs 3 and 4). Figure 3 shows the variation of absorbance values at the different dosage concentration with the increment of time. It is found that relative to the control experiment, both thiazole amide (**10**) and its hydrochloric salt (**10S**) can completely inhibit the growth of *M*. *aeruginosa* in 4–5 days at a concentration of 3.13 μg/mL or above. Therefore, the lowest complete inhibition concentration for thiazole amide (**10**) and its salts (**10S**) is determined to be 3.13 μg/mL against *M*. *aeruginosa*. The variation of growth inhibitory ratios of compounds (**10**) and (**10S**) against *M*. *aeruginosa* at the lowest complete inhibition concentration is illustrated in Fig. 4. As observed, for administration of thiazole amide (**10**), the growth inhibitory ratios after 96 and 120 hours increase gradually to 79% and 92%, respectively. Under the same experimental conditions, growth inhibitory ratios for the salt (**10S**) are 82% and 88%, respectively.

Finally, toxicity tests of this class of thiazole amide derivatives on non-target organisms were investigated preliminarily using free thiazole amide (**10**) as a representative compound (Table 3). The result demonstrates that, at a concentration as high as 500 mg/L, compound (**10**) is still none-toxic towards both terrestrial plants, *B*. *campestris* and *E*. *crusgalli*, and aquatic plants, *C*. *demersum L* and *H*. *verticillata*. Additionally, compound (**10**) is also shown to be safe towards fish *Barchydanio rerio var* at a concentration of 100 and 200 mg/L, respectively.

In summary, we have achieved a practical and scalable synthesis of bacillamide alkaloid (**1**). On the basis of the strategy, a mini compound library comprised 47 thiazole amide analogues derived from aryl- and alkylamines was prepared and investigated their algicidal activity against three harmful cyanobacterial algae, *S*. *obliqnus*, *M*. *aeruginosa*, and *C*. *pyrenoidosa*. The biological evaluation results showed that some amide analogues, such as compounds (**10**), (**10S**), (**13S**), and (**27S**), exhibit comparable algicidal activity relative to those of commercial algicide CuSO_4_ and herbicides Diuron. The preliminary results on toxic tests to non-target organisms also demonstrated that these amides are safety to terrestrial, aquatic plants, and fish. All the above-mentioned results suggest this class of thiazole amide analogues to be a novel class of algaecides against harmful Cyanobacterial algae. The studies on detailed algicidal mechanism of these thiazole amides, as well as extensive toxicity tests toward non-target organisms, are being pursued in our laboratory.

## Electronic supplementary material

Supplementary Information
