# Supplementary material for: Thiazole Amides, A Novel Class of Algaecides against Freshwater Harmful Algae
Source: Sci Rep. 2018 Jun 4;8:8555. doi: 10.1038/s41598-018-26911-6 (PMC5986738; doi:10.1038/s41598-018-26911-6)
Supplement: Supplementary file 1 — Supplementary Information [file 41598_2018_26911_MOESM1_ESM.pdf]

Supplementary Information for

Thiazole Amides, A Novel Class of Algaecides against Freshwater Harmful Algae

Ying Wang, Qisheng Liu , Zhigang Wei, Na Liu, Yajuan Li, Duo Li, Zhong Jin* and Xiaohua Xu*

State Key Laboratory of Elemento-Organic Chemistry, Collaborative Innovation Center of Chemical Science and Engineering (Tianjin), Nankai University, Tianjin 300071, People's Republic of China

Email: xiaohuaxu@nankai.edu.cn, zjin@nankai.edu.cn

NMR Spectra Data for Target Compounds

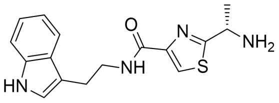

(*S*)-*N*-(2-(1*H*-Indol-3-yl)ethyl)-2-(1-aminoethyl)thiazole-4-carboxamide (alkaloid (**1**))

Light brown oil. ^{1}H NMR (400 MHz, CD_3OD) *δ*: 10.8 (brs, 1H), 8.37 (t, *J* = 6.0 Hz, 1H), 8.08 (s, 1H), 7.61 (d, *J* = 8.0 Hz,1H), 7.34 (d, *J* = 8.1 Hz,1H), 7.18 (4, *J* = 2.4 Hz, 1H), 7.06 (dd, *J* = 8.0, 1.2 Hz,1H), 6.96 (d, *J* = 6.8 Hz, 1H), 4.25 (q, *J* = 6.7 Hz, 1H), 3.56 (q, *J* = 8.0 Hz, 2H), 2.94 (t, *J* = 7.6 Hz, 2H), 2.60 (brs, 2H), 1.40 (d, *J* = 6.7 Hz, 3H); ^{13}C NMR ($CDCl_3$, 100 MHz) δ: 181.3, 161.1, 150.3, 136.7, 127.7, 123.4, 123.1, 121.4, 118.9, 118.7, 112.3, 111.8, 49.6, 39.9, 25.8, 25.1. HRMS(ESI-TOF) calcd. for $C_{16}H_{19}N_4OS^+$ $[M+H]^+$ 315.1274, found: 315.1281.

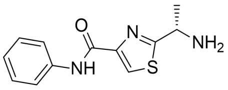

(*S*)-2-(1-Aminoethyl)-*N*-phenylthiazole-4-carboxamide (**7**)

White soild, m.p. 117–122 ºC, yield 90.5% (two steps from compound (**5**)). ^{1}H NMR ($CDCl_3$, 400 MHz) δ: 9.19 (s, 1H), 8.14 (s, 1H), 7.74 (d, *J* = 7.7 Hz, 2H), 7.40 (t, *J* = 7.9 Hz, 2H), 7.17 (t, *J* = 7.4 Hz, 1H), 4.46 (q, *J* = 6.7 Hz, 1H), 1.94 (s, 2H), 1.61 (d, *J* = 6.7 Hz, 3H); ^{13}C NMR($CDCl_3$, 100 MHz) δ: 178.9, 159.0, 149.9, 137.7, 129.0, 124.3, 123.5, 119.7, 49.7, 24.8. ESI-HRMS: calcd for $C_{12}H_{13}N_3OS$ $[M+H]^+$ 240.0825; found, 240.0856.

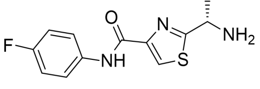

(*S*)-2-(1-Aminoethyl)-*N*-(4-fluorophenyl)thiazole-4-carboxamide (**8**)

White soild, m.p. 123–125 °C, yield 94.2% (two steps from compound (**5**)). ^{1}H NMR ($CDCl_3$, 400 MHz) δ: 9.07 (s, 1H), 8.04 (s, 1H), 7.61 (dd, *J* = 8.8, 4.8 Hz, 2H), 6.99 (t, *J* = 8.6 Hz, 2H), 4.36 (q, *J* = 6.7 Hz, 1H), 1.77 (s, 2H), 1.51 (d, *J* = 6.7 Hz, 3H); ^{13}C NMR ($CDCl_3$, 100 MHz) δ: 179.1, 160.6, 159.0, 149.7, 133.7 (d, *J* = 3.1 Hz), 123.6, 121.4 (d, *J* = 8.0 Hz), 115.7 (d, *J* = 22.5 Hz), 49.7, 24.8. ESI-HRMS: calcd for $C_{17}H_{20}FN_3O_3S$ [M+ H]$^+$ 266.0758; found, 266.0761.

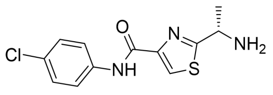

(*S*)-2-(1-Aminoethyl)-*N*-(4-chlorophenyl)thiazole-4-carboxamide (**9**)

Thick oil, yield 93.5% (two steps from compound (**5**)). ^{1}H NMR ($CDCl_3$, 400 MHz) δ: 9.17 (s, H), 8.12 (s, 1H), 7.67 (d, *J* = 8.8 Hz, 2H), 7.33 (d, *J* = 8.8 Hz, 2H), 4.43 (q, *J* = 6.7 Hz, 1H), 1.82 (s, 2H), 1.58 (d, *J* = 6.7 Hz, 3H); ^{13}C NMR ($CDCl_3$, 100 MHz) δ: 179.2, 159.0, 149.6, 136.3, 129.2, 129.0, 123.8, 120.9, 49.7, 24.8. ESI-HRMS: calcd for $C_{12}H_{12}ClN_3OS$ [M+ H]$^+$ 282.0462; found, 282.0468.

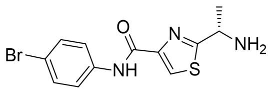

(*S*)-2-(1-Aminoethyl)-*N*-(4-bromophenyl)thiazole-4-carboxamide (**10**)

White soild, m.p. 119–121 °C, yield 92.7% (two steps from compound (**5**)). ^{1}H NMR ($CDCl_3$, 400 MHz) δ: 9.16 (s, 1H), 8.11 (s, 1H), 7.63 (d, *J* = 8.8 Hz, 2H), 7.47 (d, *J* = 8.8 Hz, 2H), 4.43 (q, *J* = 6.7 Hz, 1H), 1.82 (s, 2H), 1.58 (d, *J* = 6.7 Hz, 3H); ^{13}C NMR ($CDCl_3$, 100 MHz) δ: 179.2, 159.0, 149.6, 136.8, 132.0, 123.8, 121.2, 116.8, 49.6, 24.8. ESI-HRMS: calcd for $C_{12}H_{12}BrN_3OS$ [M+ H]$^+$ 325.9957; found, 325.9961.

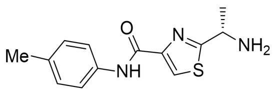

(*S*)-2-(1-Aminoethyl)-*N*-(*p*-tolyl)thiazole-4-carboxamide (**11**)

White soild, m.p. 108–111 °C, yield 93.3% (two steps from compound (**5**)). ^{1}H NMR ($CDCl_3$, 400 MHz) δ: 9.11 (s, 1H), 8.10 (s, 1H), 7.60 (d, *J* = 7.5 Hz, 2H), 7.17 (d, *J* = 7.3 Hz, 2H), 4.42 (q, *J* = 6.7 Hz, 1H), 2.34 (s, 3H), 1.85 (s, 2H), 1.58 (d, *J* = 6.1 Hz, 3H); ^{13}C NMR ($CDCl_3$, 100 MHz) δ:

178.9, 158.9, 150.0, 135.1, 133.9, 129.5, 123.3, 119.7, 49.7, 24.8, 20.9. ESI-HRMS calcd for $[C_{13}H_{15}N_3OS+H]^+$ 262.1009, found 262.1013.

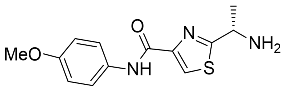

(*S*)-2-(1-Aminoethyl)-*N*-(4-methoxyphenyl)thiazole-4-carboxamide (**12**)

White soild, m.p. 126–127 °C, yield 92.3% (two steps from compound (**5**)). ^{1}H NMR ($CDCl_3$, 400 MHz) δ: 9.07 (s, 1H), 8.09 (s, 1H), 7.62 (d, *J* = 8.4 Hz, 2H), 6.91 (d, *J* = 8.4 Hz, 2H), 4.42 (q, *J* = 6.6 Hz, 1H), 3.81 (s, 3H), 1.83 (s, 2H), 1.58 (d, *J* = 6.6 Hz, 3H); ^{13}C NMR ($CDCl_3$, 100 MHz) δ: 178.9, 158.8, 156.4, 150.0, 130.9, 123.2, 121.4, 114.2, 55.4, 49.7, 24.8. ESI-HRMS: calcd for $C_{13}H_{15}N_3O_2S$ $[M+H]^+$ 278.0958; found, 278.0960.

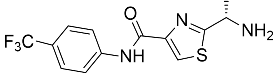

(*S*)-2-(1-Aminoethyl)-*N*-(4-(trifluoromethyl)phenyl)thiazole-4-carboxamide (**13**)

White soild, m.p. 139–141 °C, yield 89.7% (two steps from compound (**5**)). ^{1}H NMR ($CDCl_3$, 400 MHz) δ: 9.25 (s,1H), 8.08 (s,1H), 7.78 (d, *J* = 8.5 Hz, 2H), 7.56 (d, *J* = 8.5 Hz, 2H), 4.38 (q, *J* = 6.7 Hz, 1H), 1.76 (s, 2H), 1.52 (d, *J* = 6.7 Hz, 3H); ^{13}C NMR ($CDCl_3$, 100 MHz) δ: 179.4, 159.2, 149.4, 140.8, 126.3 (q, *J* = 3.9 Hz), 124.3, 119.3, 49.7, 24.8. ESI-HRMS calcd for $[C_{13}H_{12}F_3N_3OS+H]^+$ 316.0726, found 316.0731

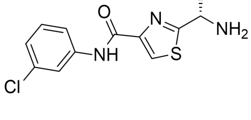

(*S*)-2-(1-Aminoethyl)-*N*-(3-chlorophenyl)thiazole-4-carboxamide (**14**)

White soild, m.p.155–159 °C, yield 90.5% (two steps from compound (**5**)). ^{1}H NMR ($CDCl_3$, 400 MHz) δ: 9.21 (s, 1H), 8.16 (s, 1H), 7.86 (s, 1H), 7.61 (d, *J* = 8.0 Hz, 1H), 7.32 (t, *J* = 8.0 Hz, 1H), 7.14 (d, *J* = 7.8 Hz, 1H), 4.46 (q, *J* = 6.6 Hz, 1H), 1.85 (s, 2H), 1.62 (d, *J* = 6.6 Hz, 3H); ^{13}C NMR ($CDCl_3$, 100 MHz) δ: 179.2, 159.0, 149.5, 138.9, 134.7, 130.0, 124.3, 124.0, 119.7, 117.7, 49.7, 24.8. ESI-HRMS: calcd for $C_{12}H_{12}ClN_3OS$ $[M+H]^+$, 282.0462; found, 282.0467.

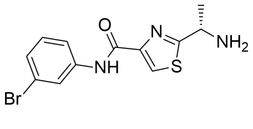

(S)-2-(1-Aminoethyl)-*N*-(3-bromophenyl)thiazole-4-carboxamide (**15**)

Yellow soild, m.p. 157–159 ºC, yield 91.3% (two steps from compound (**5**)). ^{1}H NMR ($CDCl_3$, 400 MHz) δ: 9.17 (s, 1H), 8.12 (s, 1H), 7.96 (t, J = 1.8 Hz, 1H), 7.65 (sd, J =8.0 Hz, J = 1.6 Hz, 1H), 7.29 – 7.20 (m, 2H), 4.43 (q, J = 6.7 Hz, 1H), 1.81 (s, 2H), 1.58 (d, J = 6.7 Hz, 3H); ^{13}C NMR ($CDCl_3$, 100 MHz) δ: 179.2, 159.0, 149.5, 139.0, 130.3, 127.2, 124.0, 122.6, 122.5, 118.1, 49.6, 24.8. ESI-HRMS: calcd for $C_{12}H_{12}BrN_3OS$ [M+ H]$^+$ 325.9957; found, 325.9960.

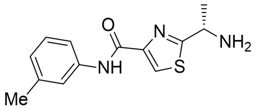

(S)-2-(1-Aminoethyl)-*N*-(*m*-tolyl)thiazole-4-carboxamide (**16**).

White soild, m.p. 135–137 ºC, yield 91.4% (two steps from compound (**5**)). ^{1}H NMR ($CDCl_3$, 400 MHz) δ: 9.15 (s, 1H), 8.13 (s, 1H), 7.59 (s, 1H), 7.54(d, J = 8.1 Hz, 1H), 7.29 (t, J = 3.8 Hz, 2H), 6.99 (d, J = 7.5 Hz, 1H), 4.46 (q, J = 6.7 Hz, 1H), 2.40 (s, 3H), 1.85 (s, 2H), 1.61 (d, J = 6.7 Hz, 3H); ^{13}C NMR ($CDCl_3$, 100 MHz) δ: 178.9, 159.0, 150.0, 138.9, 137.6, 128.8, 125.1, 123.4, 120.3, 116.8, 49.7, 24.8, 21.5. ESI-HRMS: calcd for $C_{13}H_{15}N_3OS$ [M+ H]$^+$ 262.1009; found, 262.1009.

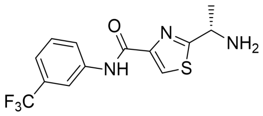

(S)-2-(1-Aminoethyl)-*N*-(3-(trifluoromethyl)phenyl)thiazole-4-carboxamide (**17**)

Thick oil , yield 86.6% (two steps from compound (**5**)). ^{1}H NMR ($CDCl_3$, 400 MHz) δ: 9.31 (s, 1H), 8.17 (s, 1H), 8.03 (s, 1H), 7.98 (d, J = 8.1 Hz, 2H), 7.52 (t, J = 7.8 Hz, 1H), 7.42 (d, J = 7.7 Hz, 1H), 4.47 (q, J = 6.6 Hz, 1H), 1.86 (s, 2H), 1.62 (d, J = 6.6 Hz, 3H); ^{13}C NMR ($CDCl_3$, 100 MHz) δ: 179.4, 159.2, 149.4, 138.3, 129.6, 124.1, 122.7, 120.8 (q, J = 3.8 Hz), 116.3 (q, J = 3.7 Hz), 49.7, 24.8. ESI-HRMS: calcd for $C_{13}H_{12}F_3N_3OS$ [M+ H]$^+$ 316.0726; found, 316.0729.

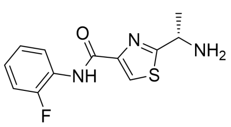

(S)-2-(1-Aminoethyl)-*N*-(2-fluorophenyl)thiazole-4-carboxamide (**18**)

White soild, m.p. 98–104 ºC, yield 93.6% (two steps from compound (**5**)). ^{1}H NMR ($CDCl_3$, 400 MHz) δ: 9.40 (s,1H), 8.43 (t, *J* = 8.1 Hz, 1H), 8.06 (s, 1H), 7.15-6.95 (m, 3H), 4.37 (q, *J* = 6.6 Hz, 1H), 1.81 (s, 2H), 1.52 (d, *J* = 6.7 Hz, 3H); ^{13}C NMR ($CDCl_3$, 100 MHz) δ: 179.1, 159.0, 152.5 (d, *J* = 243.6 Hz), 149.6, 126.3 (d, *J* = 10.1 Hz), 124.6 (d, *J* = 3.8 Hz), 124.3 (d, *J* = 7.7 Hz), 123.9, 121.4, 114.8 (d, *J* = 19.1 Hz), 49.7, 24.7. ESI-HRMS: calcd for $C_{12}H_{12}FN_3OS$ $[M+H]^+$ 266.0758; found, 266.0762

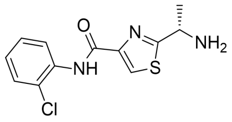

(*S*)-2-(1-Aminoethyl)-*N*-(2-chlorophenyl)thiazole-4-carboxamide (**19**)

White soild, m.p. 131–134 ºC, yield 87.7% (two steps from compound (**5**)). ^{1}H NMR ($CDCl_3$, 400 MHz) δ: 9.91 (s, 1H), 8.61 (d, *J* = 8.2 Hz, 1H), 8.15 (s, 1H), 7.43 (d, *J* = 8.0 Hz, 1H), 7.34 (t, *J* = 7.8 Hz, 1H), 7.08 (t, *J* = 7.7 Hz, 1H), 4.45 (q, *J* = 6.7 Hz, 1H), 1.85 (s, 2H), 1.62 (d, *J* = 6.7 Hz, 3H); ^{13}C NMR ($CDCl_3$, 100 MHz) δ: 179.0, 159.0, 149.7, 134.7, 129.1, 127.7, 124.5, 124.0, 123.0, 121.1, 49.7, 24.7. ESI-HRMS: calcd for $C_{12}H_{12}ClN_3OS$ $[M+H]^+$ 282.0462; found, 282.0466.

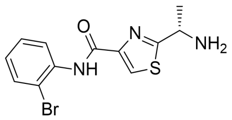

(*S*)-2-(1-Aminoethyl)-*N*-(2-bromophenyl)thiazole-4-carboxamide (**20**)

White soild, m.p. 126–128 ºC, yield 92.3% (two steps from compound (**5**)). ^{1}H NMR ($CDCl_3$, 400 MHz) δ: 9.95 (s, 1H), 8.59 (d, *J* = 8.2 Hz, 1H), 8.16 (s, 1H), 7.60 (d, *J* = 7.9 Hz, 1H), 7.38 (t, *J* = 7.7 Hz, 1H), 7.02 (t, *J* = 7.5 Hz, 1H), 4.46 (d, *J* = 6.4 Hz, 1H), 1.91 (s, 2H), 1.64 (d, *J* = 6.2 Hz, 3H); ^{13}C NMR ($CDCl_3$, 100 MHz) δ: 178.9, 159.1, 149.7, 135.9, 132.4, 128.4, 125.0, 124.0, 121.3, 113.5, 49.7, 24.7. ESI-HRMS: calcd for $C_{12}H_{12}BrN_3OS$ $[M+H]^+$ 325.9957; found, 325.9959.

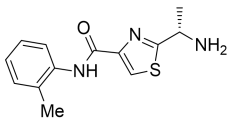

(*S*)-2-(1-aminoethyl)-*N*-(*o*-tolyl)thiazole-4-carboxamide (**21**)

White soild, m.p. 136–138 ºC , yield 90.1% (two steps from compound (**5**)). ^{1}H NMR ($CDCl_3$, 400 MHz) δ: 9.22 (s, 1H), 8.17 (d, *J* = 8.1 Hz, 1H), 8.12 (s, 1H), 7.33–7.16 (m, 2H), 7.08 (t, *J* = 7.4 Hz,

1H), 4.42 (q, J = 6.7 Hz, 1H), 2.37 (s, 3H), 1.87 (s, 2H), 1.59 (d, J = 6.7 Hz, 3H); ^{13}C NMR ($CDCl_3$, 100 MHz) δ: 178.8, 158.9, 150.1, 135.8, 130.4, 127.9, 126.8, 124.6, 123.4, 121.5, 49.7, 24.7, 17.6. ESI-HRMS: calcd for $C_{13}H_{15}N_3OS$ $[M+ H]^+$ 262.1009; found, 262.1013.

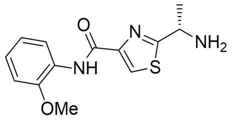

(*S*)-2-(1-Aminoethyl)-*N*-(2-methoxyphenyl)thiazole-4-carboxamide (**22**)

White soild, m.p. 82–84 °C , yield 90.5% (two steps from compound (**5**)). ^1H NMR ($CDCl_3$, 400 MHz) δ: 9.80 (s, 1H), 8.55 (dd, J = 7.9, 1.3 Hz, 1H), 8.12 (s, 1H), 7.10 (td, J = 7.8, 1.5 Hz, 1H), 7.03 (t, J = 7.7 Hz, 1H), 6.94 (d, J = 8.0 Hz, 1H), 4.45 (q, J = 6.7 Hz, 1H), 3.96 (s, 3H), 1.87 (s, 2H), 1.61 (d, J = 6.7 Hz, 3H); ^{13}C NMR ($CDCl_3$, 100 MHz) δ: 178.7, 159.0, 150.3, 148.4, 127.5, 123.9, 123.3, 121.1, 119.8, 110.0, 55.8, 49.7, 24.7. ESI-HRMS: calcd for $C_{13}H_{15}N_3O_2S$ $[M+ H]^+$ 278.0958; found, 278.0962.

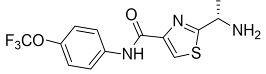

(*S*)-2-(1-Aminoethyl)-*N*-(4-(trifluoromethoxy)phenyl)thiazole-4-carboxamide (**23**)

White soild, m.p. 126–127 °C, yield 87.4% (two steps from compound (**5**)). ^1H NMR ($CDCl_3$, 400 MHz) δ: 9.15 (brs, 1H), 8.06 (s, 1H), 7.67 (d, J = 8.4 Hz, 2H), 7.15 (d, J = 8.4 Hz, 2H), 4.25 (q, J = 6.6 Hz, 1H), 2.17 (brs, 2H), 1.51 (d, J = 6.6 Hz, 3H); ^{13}C NMR ($CDCl_3$, 100 MHz) δ: 178.8, 159.5, 149.5, 145.4, 136.7, 124.0, 121.9, 120.8, 49.5, 24.6. ESI-HRMS: calcd for $C_{13}H_{15}N_3O_2S$ $[M+ H]^+$ 278.0958; found, 278.0960.

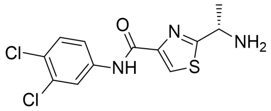

(S)-2-(1-Aminoethyl)-*N*-(3,4-dichlorophenyl)thiazole-4-carboxamide (**24**)

White soild, m.p. 121–124 °C, yield 87.7% (two steps from compound (**5**)). ^1H NMR ($CDCl_3$, 400 MHz) δ: 9.11 (s, 1H), 8.06 (s, 1H), 7.88 (d, J = 2.4 Hz, 1H), 7.50 (dd, J = 8.8, 2.4 Hz, 1H), 7.35 (d, J = 8.8 Hz, 1H), 4.36 (q, J = 6.7 Hz, 1H), 1.74 (s, 2H), 1.51 (d, J = 6.7 Hz, 3H); ^{13}C NMR ($CDCl_3$,

100 MHz) δ: 179.4, 159.0, 149.2, 137.2, 132.8, 130.5, 127.4, 124.2, 121.3, 118.9, 49.6, 24.8. ESI-HRMS: calcd for $C_{12}H_{11}Cl_{2}N_3OS$ $[M+ H]^+$ 316.2062; found, 316.2060.

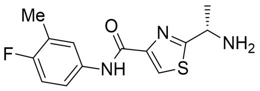

(*S*)-2-(1-aminoethyl)-*N*-(4-fluoro-3-methylphenyl)thiazole-4-carboxamide (**25**)

White soild, m.p. 128–130 °C, yield 79.6% (two steps from compound (**5**)). ^{1}H NMR ($CDCl_3$, 400 MHz) δ: 9.10 (s, 1H), 8.12 (s, 1H), 7.59 (dd, *J* = 6.8, 2.5 Hz, 1H), 7.51 – 7.47 (m, 1H), 7.01 (t, *J* = 9.0 Hz, 1H), 4.44 (q, *J* = 6.7 Hz, 1H), 2.31 (d, *J* = 1.8 Hz, 3H), 1.87 (s, 2H), 1.60 (d, *J* = 6.7 Hz, 3H); ^{13}C NMR ($CDCl_3$, 100 MHz) δ: 179.0, 158.9, 149.8, 133.3, 125.5, 125.3, 123.5, 122.9, 118.7, 115.3, 49.7, 24.8, 14.7. ESI-HRMS: calcd for $C_{10}H_{17}N_3OS$ $[M+H]^+$ 279.3332; found, 279.3334.

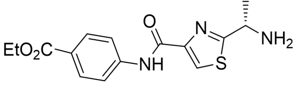

(*S*)-Ethyl 4-(2-(1-aminoethyl)thiazole-4-carboxamido)benzoate (**26**)

White soild, m.p. 109–111 °C, yield 92.5% (two steps from compound (**5**)). ^{1}H NMR ($CDCl_3$, 400 MHz) δ: 9.27 (s, 1H), 8.08 (s, 1H), 8.00 (d, *J* = 8.6 Hz, 2H), 7.74 (d, *J* = 8.6 Hz, 2H), 4.38 (q, *J* = 6.7 Hz, 1H), 4.31 (q, *J* = 7.1 Hz, 2H), 1.86 (s, 2H), 1.52 (d, *J* = 6.7 Hz, 3H), 1.33 (t, *J* = 7.1 Hz, 3H); ^{13}C NMR ($CDCl_3$,100 MHz) δ: 179.2, 166.1, 159.1, 149.5, 141.8, 130.8, 126.0, 124.2, 118.8, 60.8, 49.6, 24.8, 14.3. ESI-HRMS: calcd for $C_{15}H_{17}N_3O_3S$ $[M+H]^+$ 320.1063; found, 320.1066.

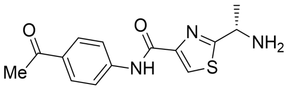

(*S*)-*N*-(4-Acetylphenyl)-2-(1-aminoethyl)thiazole-4-carboxamide (**27**)

White soild, m.p. 139–141 °C, yield 93.3% (two steps from compound (**5**)). ^{1}H NMR ($CDCl_3$, 400 MHz) δ: 9.30 (s, 1H), 8.09 (s, 1H), 7.92 (d, *J* = 8.2 Hz, 2H), 7.76 (d, *J* = 8.1 Hz, 2H), 4.38 (q, *J* = 6.4 Hz, 1H), 2.53 (s, 3H), 1.97 (s, 2H), 1.52 (d, *J* = 6.5 Hz, 3H); ^{13}C NMR ($CDCl_3$, 100 MHz) δ: 196.9, 179.4, 159.1, 149.4, 142.0, 132.9, 129.8, 124.3, 118.9, 49.6, 26.4, 24.8. ESI-HRMS: calcd for $C_{14}H_{15}N_3O_2S$ $[M+H]^+$ 290.0958; found, 290.0957.

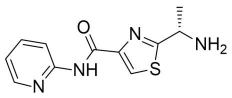

(S)-2-(1-Aminoethyl)-N-(pyridin-2-yl)thiazole-4-carboxamide (**28**)

White soild, m.p. 93–94 °C, yield 89.4% (two steps from compound (**5**)). ^{1}H NMR ($CDCl_3$, 400 MHz) δ: 9.77 (s, 1H), 8.39 (d, J = 8.4 Hz, 1H), 8.36 (d, J = 4.9 Hz, 1H), 8.17 (s, 1H), 7.77 (t, J = 7.8 Hz, 1H), 7.09 (t, J = 6.0 Hz, 1H), 4.42 (q, J = 6.6 Hz, 1H), 1.85 (s, 2H), 1.59 (d, J = 6.6 Hz, 3H); ^{13}C NMR ($CDCl_3$,100 MHz) δ: 179.0, 159.3, 151.2, 149.5, 148.1, 138.3, 124.2, 119.8, 114.0, 49.7, 24.7. ESI-HRMS: calcd for $C_{11}H_{12}N_4OS$ $[M+H]^+$ 249.0805; found, 249.0809.

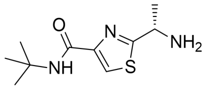

(S)-2-(1-Aminoethyl)-N-(*tert*-butyl)thiazole-4-carboxamide (**29**)

White soild, m.p. 75–78 °C, yield 92.3% (two steps from compound (**5**)). ^{1}H NMR ($CDCl_3$, 400 MHz) δ: 7.95 (s, 1H), 7.22 (s, 1H), 4.37 (q, J = 6.6 Hz, 1H), 1.89 (s, 2H), 1.54 (d, J = 6.7 Hz, 3H), 1.48 (s, 9H); ^{13}C NMR ($CDCl_3$, 100 MHz) δ: 178.2, 160.6, 150.9, 122.0, 51.1, 49.6, 28.8, 24.7. ESI-HRMS: calcd for $C_{10}H_{17}N_3OS$ $[M+H]^+$ 228.1165; found, 228.1168.

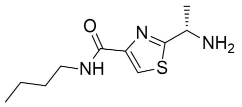

(S)-2-(1-Aminoethyl)-N-butylthiazole-4-carboxamide (**30**)

White soild, m.p. 109–111 °C, yield 93.6% (two steps from compound (**5**)). ^{1}H NMR ($CDCl_3$, 400 MHz) δ: 8.00 (s, 1H), 7.32 (s, 1H), 4.38 (q, J = 6.7 Hz, 1H), 3.53 – 3.36 (m, 2H), 1.98 (s, 2H), 1.65 – 1.57 (m, 2H), 1.55 (d, J = 6.7 Hz, 3H), 1.46 – 1.36 (m, 2H), 0.98 (t, J = 11.2 Hz 3H); ^{13}C NMR ($CDCl_3$, 100 MHz) δ: 178.4, 161.2, 150.0, 122.5, 49.6, 39.0, 31.8, 24.7, 20.1, 13.7. ESI-HRMS: calcd for $C_{10}H_{17}N_3OS$ $[M+H]^+$ 228.1165; found, 228.1164.

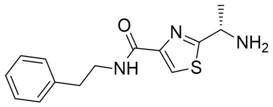

(S)-2-(1-Aminoethyl)-N-phenethylthiazole-4-carboxamide (**31**)

White soild, m.p. 141–143 °C, yield 82.4% (two steps from compound (**5**)). ^{1}H NMR (400 MHz, $CDCl_3$) δ: 8.77 (s, 1H), 8.01 (s, 1H), 7.42 (s, 1H), 7.32 (t, *J* = 8.0 Hz, 2H), 7.24 (t, *J* = 8.0 Hz, 2H), 4.42 (q, *J* = 6.6 Hz, 1H), 1.85 (s, 2H), 1.59 (d, *J* = 6.6 Hz, 3H); ^{13}C NMR (100 MHz, $CDCl_3$) δ: 179.0, 159.3, 151.2, 149.5, 148.1, 138.3, 124.2, 119.8, 114.0, 49.7, 24.7. ESI- HRMS: calcd for $C_{14}H_{17}N_3OS$ $[M+H]^+$ 249.0805; found,249.0809.

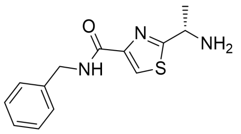

(*S*)-2-(1-Aminoethyl)-*N*-benzylthiazole-4-carboxamide (**32**)

White soild, m.p. 64–67 °C, yield 94.6% (two steps from compound (**5**)). ^{1}H NMR ($CDCl_3$, 400 MHz) δ: 8.07 (s, 1H), 7.66 (s, 1H), 7.43 – 7.31 (m, 5H), 4.66 (d, *J* = 6.0 Hz, 2H), 4.36 (q, *J* = 6.6 Hz, 1H), 1.96 (s, 2H), 1.54 (d, *J* = 6.7 Hz, 3H); ^{13}C NMR ($CDCl_3$, 100 MHz) δ: 178.6, 161.2, 149.7, 138.3, 128.7, 127.9, 127.5, 123.0, 49.6, 43.3, 24.7. ESI-HRMS: calcd for $C_{13}H_{15}N_3OS$ $[M+H]^+$ 262.1009; found, 262.1012.

NMR Spectra Data

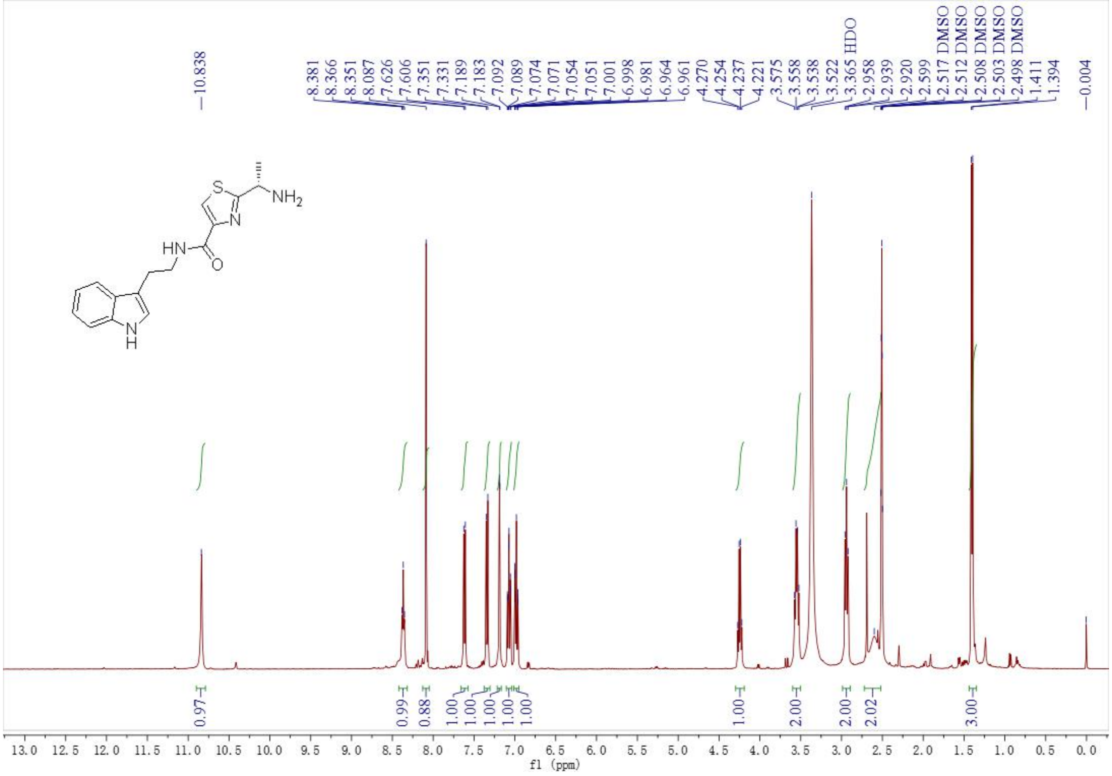

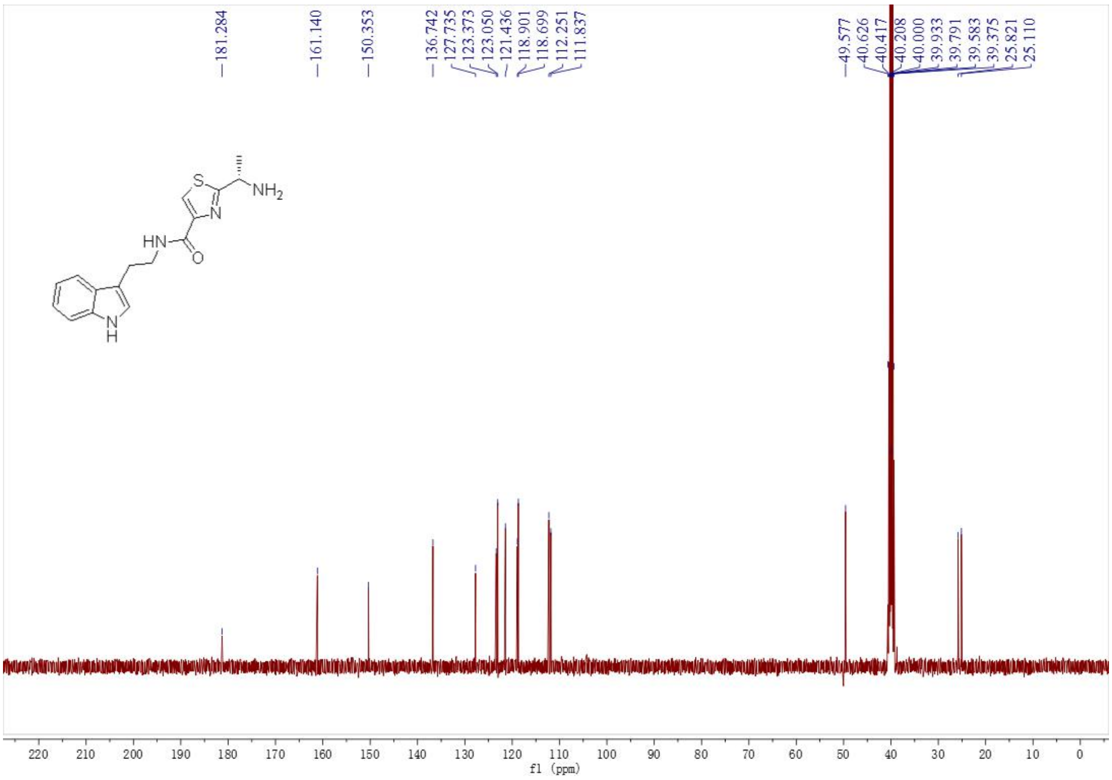

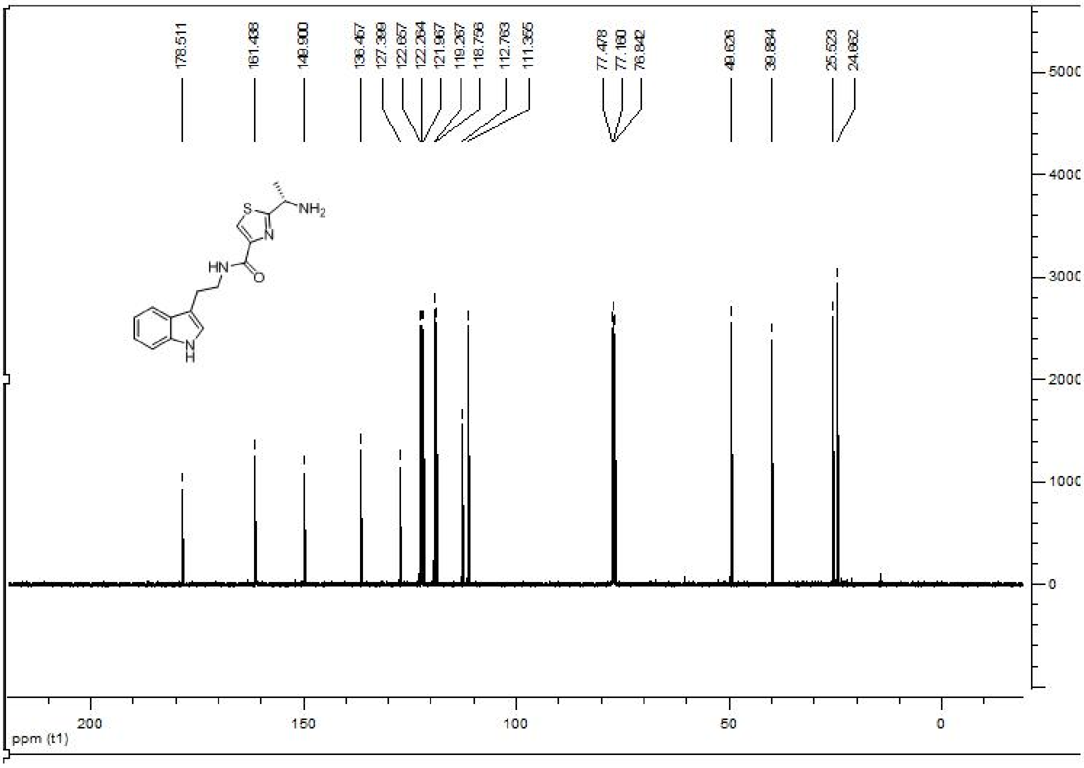
178.511
161.438
149.900
136.457
127.399
122.667
122.264
121.967
119.267
118.756
112.763
111.355
77.478
77.160
76.842
49.626
39.884
25.523
24.862
NH2
S
N
HN
O
H
N
5000
4000
3000
2000
1000
0
200
150
100
50
0
ppm (t1)

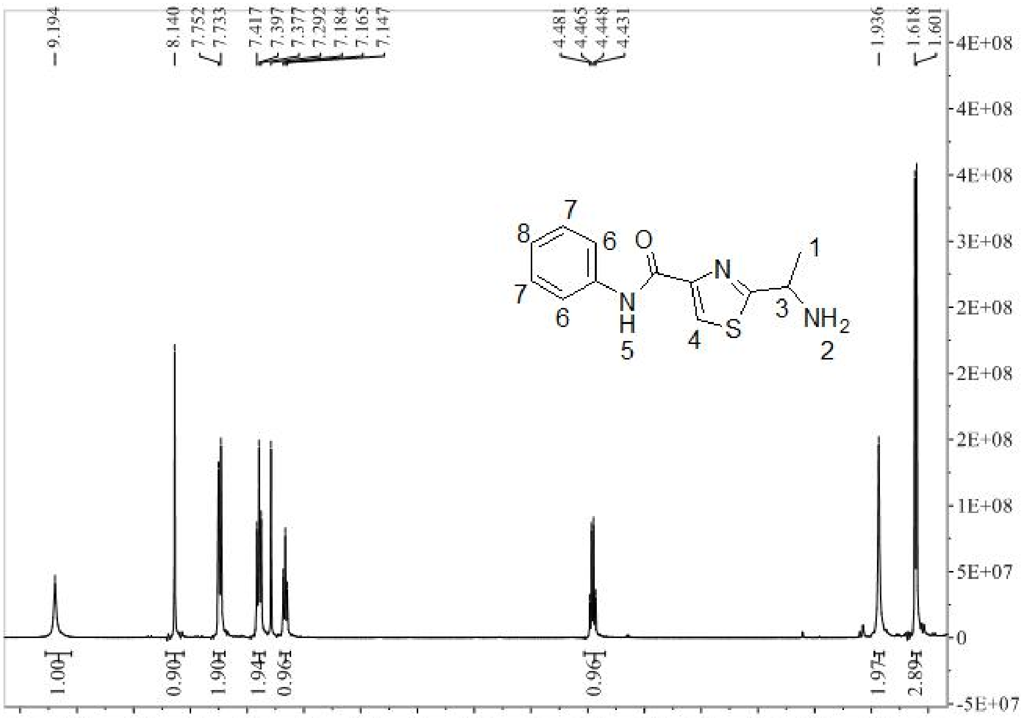
9.194
8.140
7.752
7.733
7.417
7.397
7.377
7.292
7.184
7.165
7.147
4.481
4.465
4.448
4.431
1.936
1.618
1.601
1
2
3
4
5
6
7
8
O
N
S
N
H
NH2
1.00
0.90
1.90
1.94
0.96
0.96
1.97
2.89
4E+08
3E+08
2E+08
1E+08
5E+07
0
-5E+07
9.5
9.0
8.5
8.0
7.5
7.0
6.5
6.0
5.5
5.0
4.5
4.0
3.5
3.0
2.5
2.0
1.5
ppm

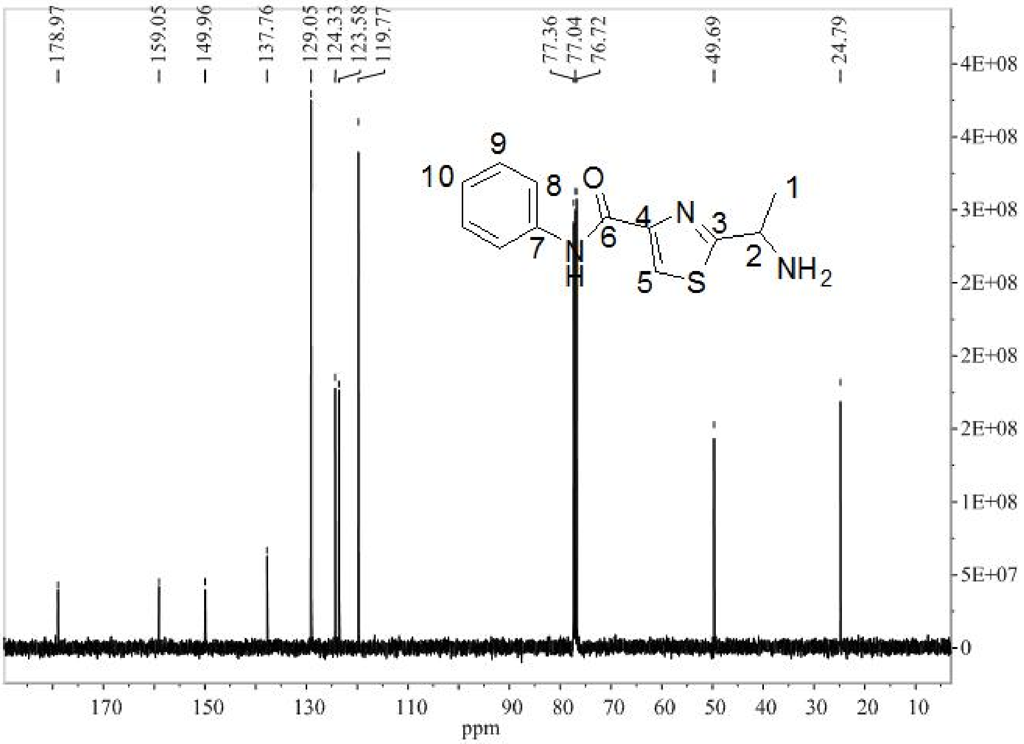
178.97
159.05
149.96
137.76
129.05
124.33
123.58
119.77
77.36
77.04
76.72
49.69
24.79
4E+08
4E+08
3E+08
2E+08
2E+08
2E+08
1E+08
5E+07
0
170 150 130 110 90 80 70 60 50 40 30 20 10
ppm
9
10
8
O
1
N
3
4
6
7
N
H
2
NH2
5
S

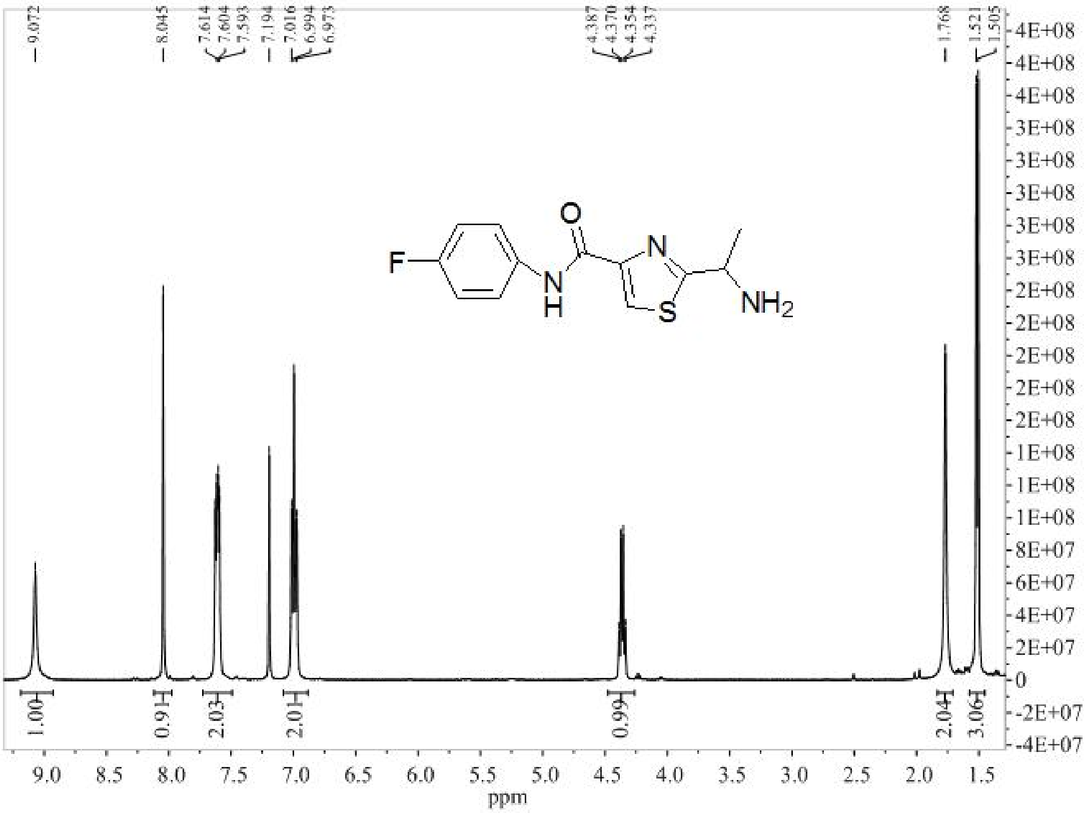
9.072
8.045
7.614
7.604
7.593
7.194
7.016
6.994
6.973
4.387
4.370
4.354
4.337
1.768
1.521
1.505
1.00
0.91
2.03
2.01
0.99
2.04
3.06
9.0 8.5 8.0 7.5 7.0 6.5 6.0 5.5 5.0 4.5 4.0 3.5 3.0 2.5 2.0 1.5
ppm
F
O
N
N
H
S
NH2

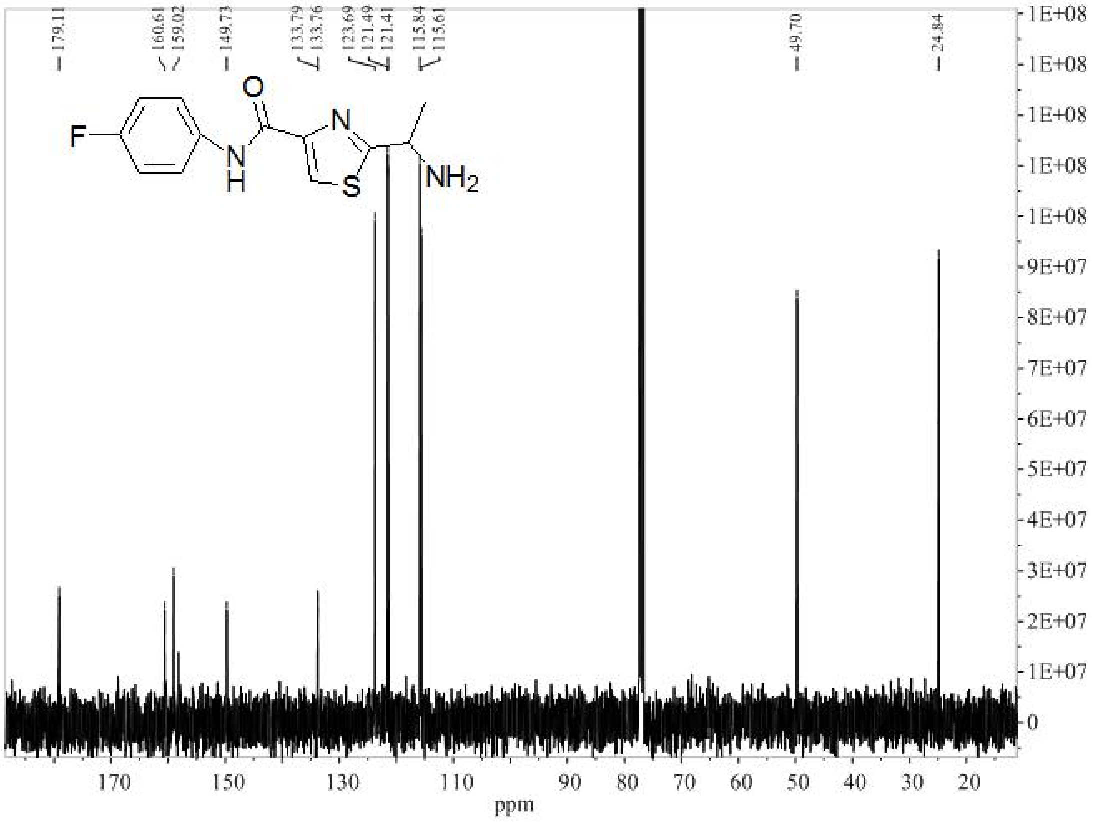

179.11
160.61
159.02
149.73
133.79
133.76
123.69
121.49
121.41
115.84
115.61
49.70
24.84
F
O
N
H
S
NH2
ppm

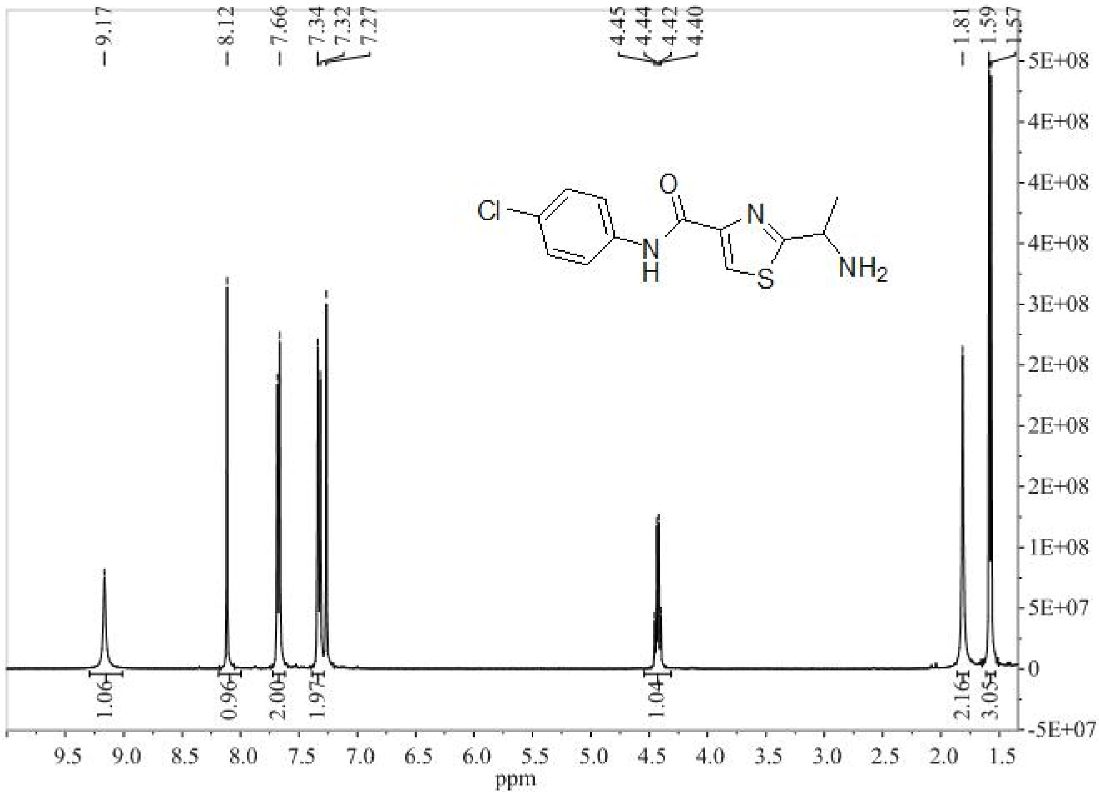

9.17
8.12
7.66
7.34
7.32
7.27
4.45
4.44
4.42
4.40
1.81
1.59
1.57
Cl
O
N
H
S
NH2
1.06
0.96
2.00
1.97
1.04
2.16
3.05
ppm

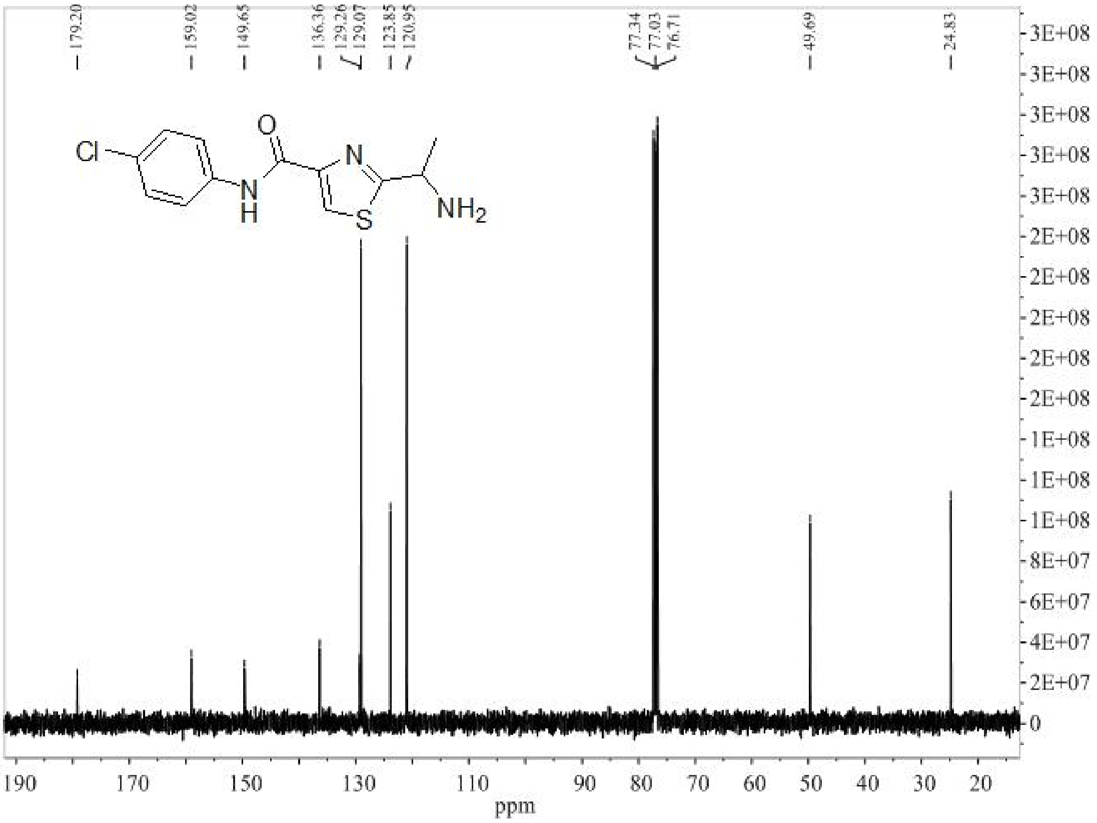
179.20
159.02
149.65
136.36
129.26
129.07
123.85
120.95
77.34
77.03
76.71
49.69
24.83
Cl
O
N
H
N
S
NH2
ppm

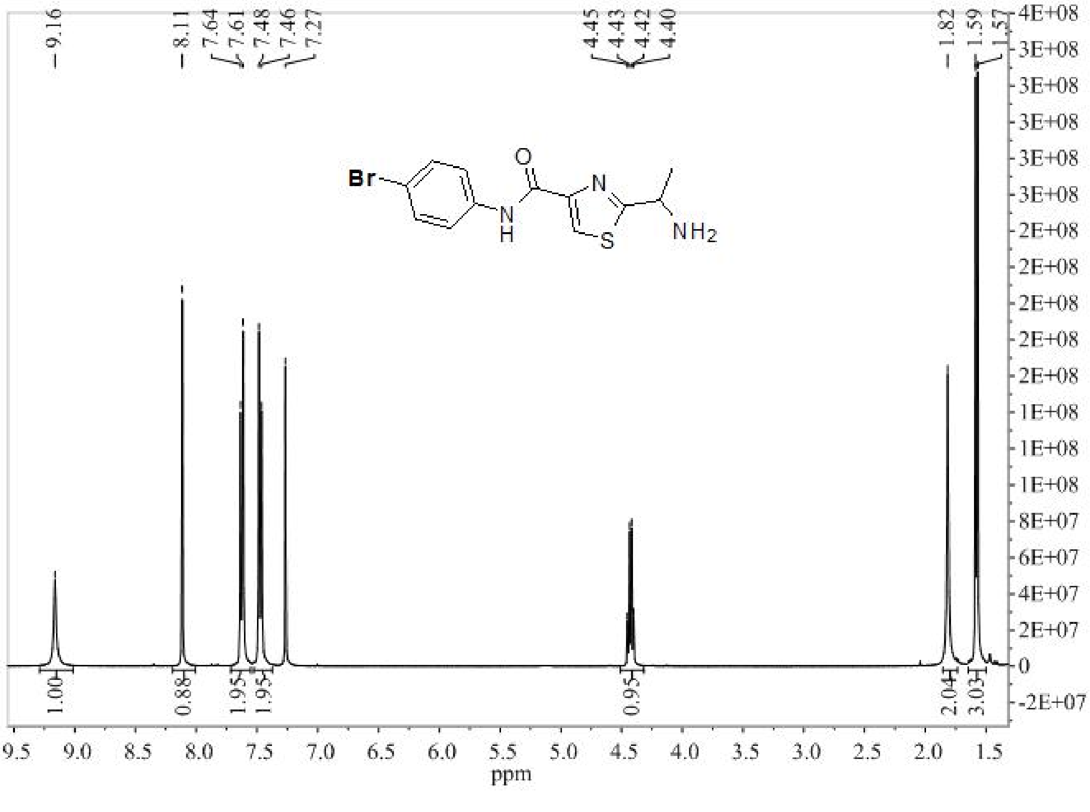
9.16
8.11
7.64
7.61
7.48
7.46
7.27
4.45
4.43
4.42
4.40
1.82
1.59
1.57
Br
O
N
H
N
S
NH2
1.00
0.88
1.95
1.95
0.95
2.04
3.03
ppm

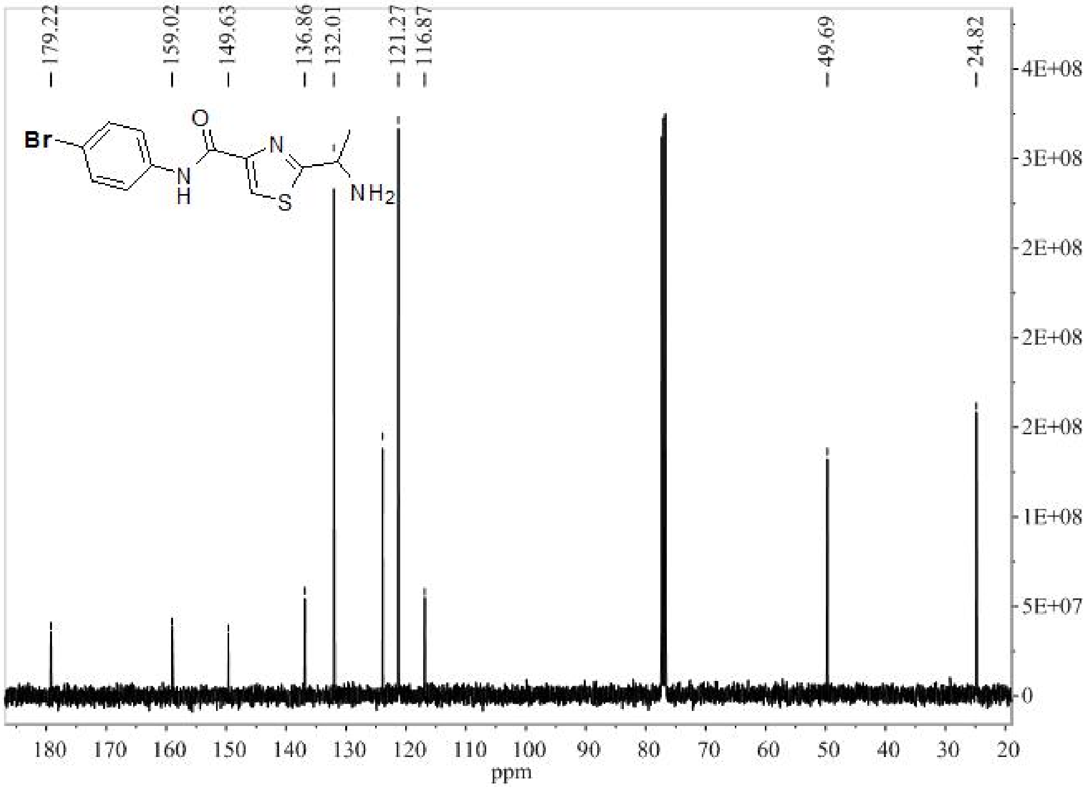
179.22
159.02
149.63
136.86
132.01
121.27
116.87
49.69
24.82
Br
O
N
H
N
S
NH2
ppm

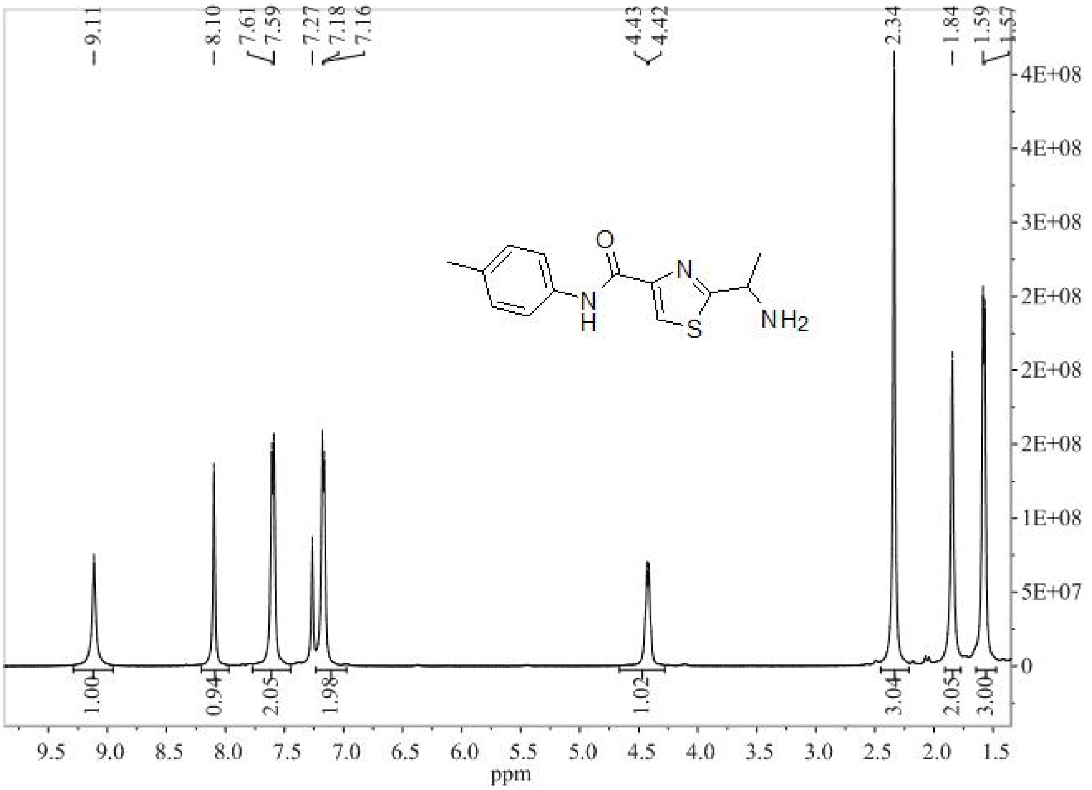
9.11
8.10
7.61
7.59
7.27
7.18
7.16
4.43
4.42
2.34
1.84
1.59
1.57
O
N
H
N
S
NH2
1.00
0.94
2.05
1.98
1.02
3.04
2.05
3.00
ppm

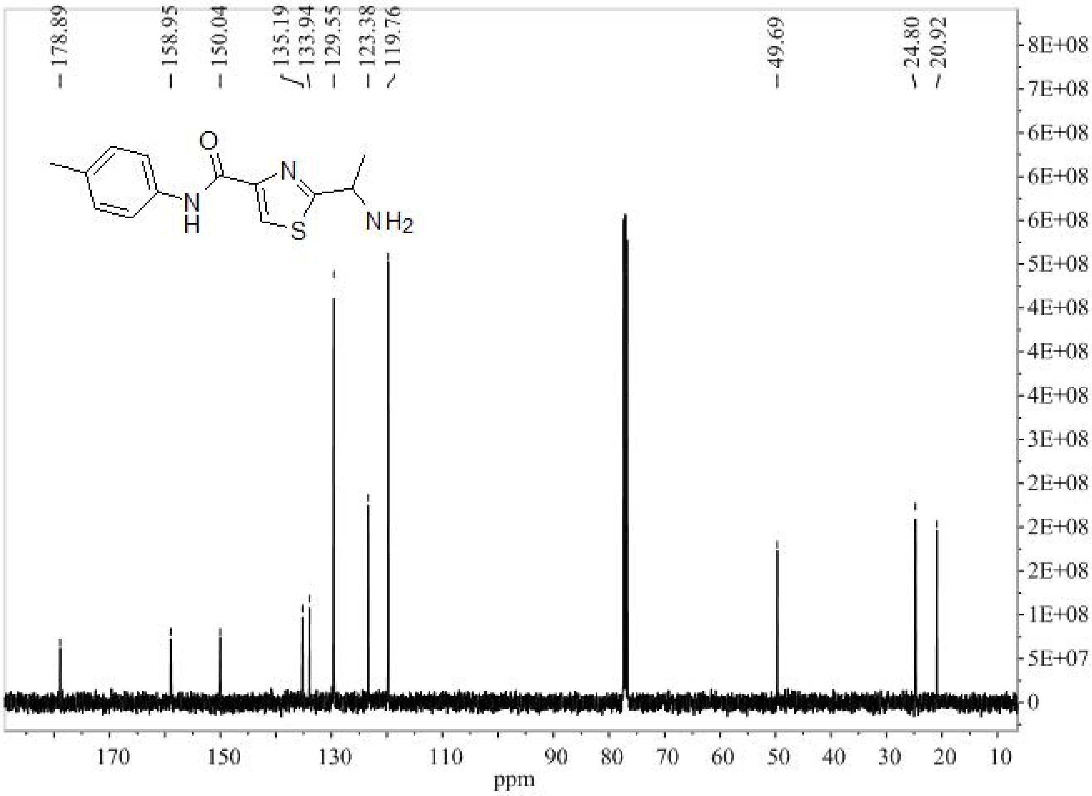

178.89
158.95
150.04
135.19
133.94
129.55
123.38
119.76
49.69
24.80
20.92
O
N
H
S
NH2
ppm

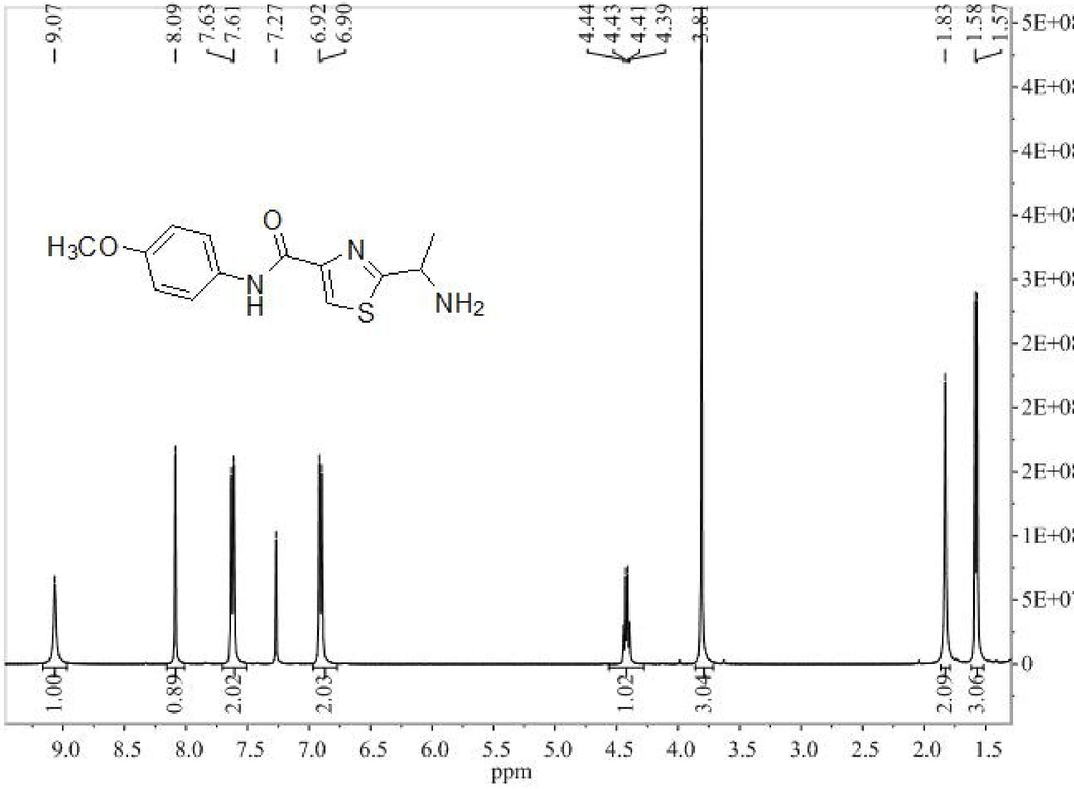

9.07
8.09
7.63
7.61
7.27
6.92
6.90
4.44
4.43
4.41
4.39
3.81
1.83
1.58
1.57
H3CO
O
N
H
S
NH2
1.00
0.89
2.02
2.03
1.02
3.04
2.09
3.06
ppm

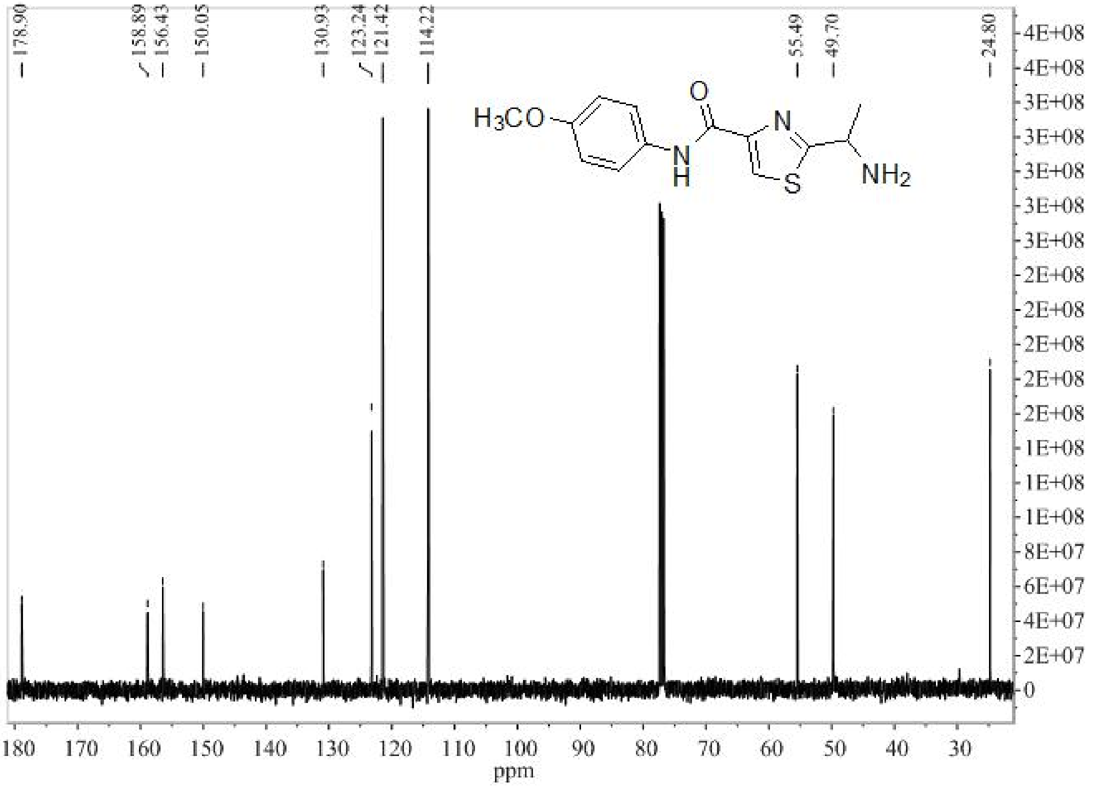

178.90
158.89
156.43
150.05
130.93
123.24
121.42
114.22
55.49
49.70
24.80
H3CO
O
N
N
H
S
NH2
ppm

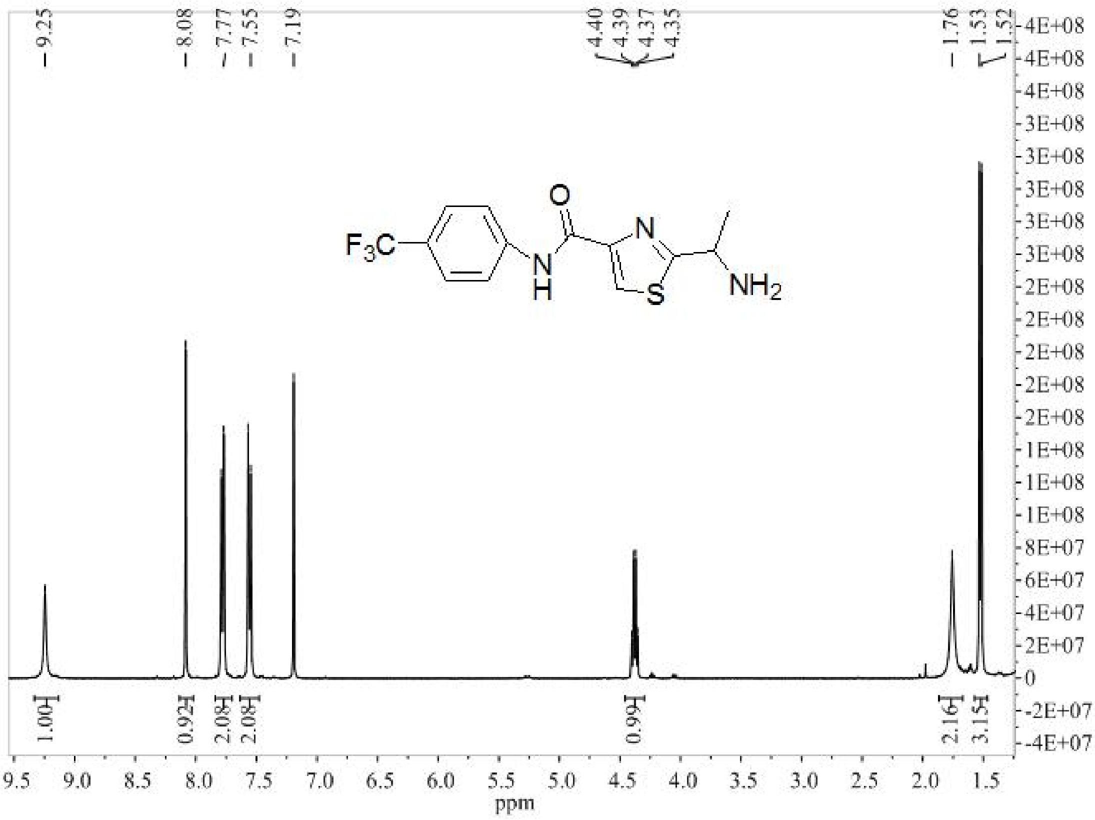

9.25
8.08
7.77
7.55
7.19
4.40
4.39
4.37
4.35
1.76
1.53
1.52
F3C
O
N
N
H
S
NH2
1.00
0.92
2.08
2.08
0.99
2.16
3.15
ppm

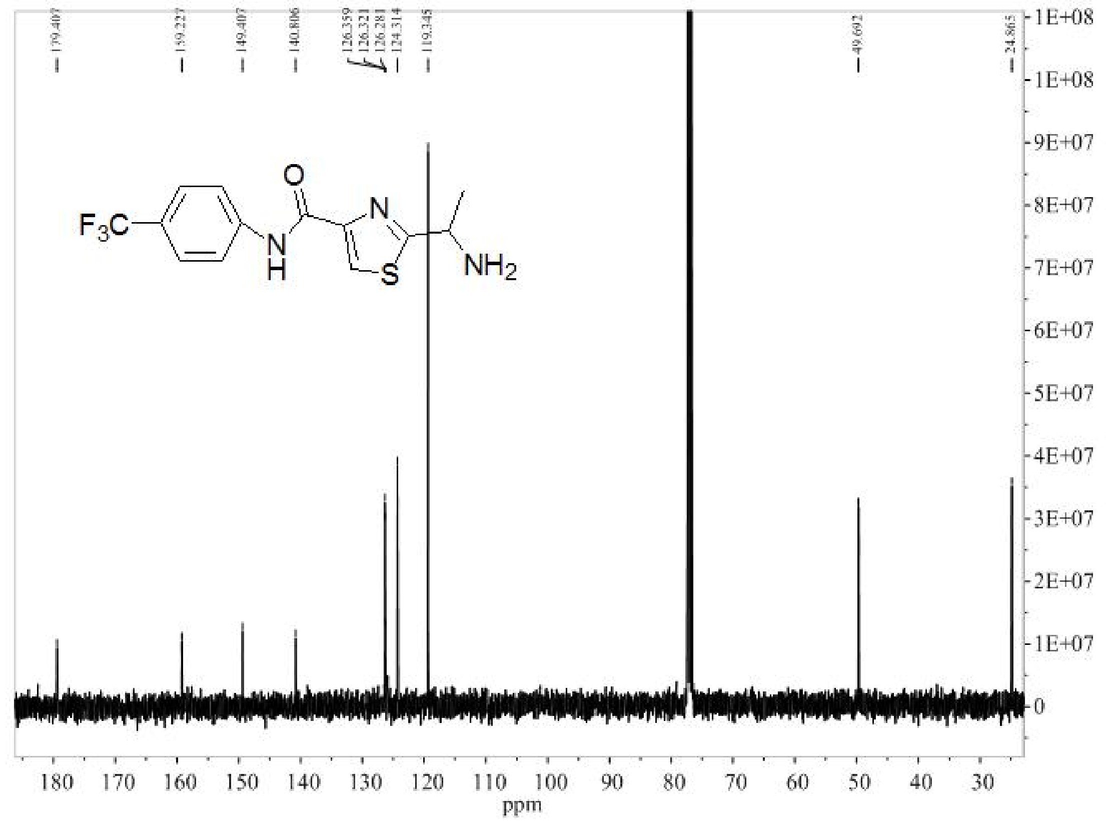

179.407
159.227
149.407
140.806
126.359
126.321
126.281
124.314
119.345
49.692
24.863
F3C
O
N
H
N
S
NH2
ppm

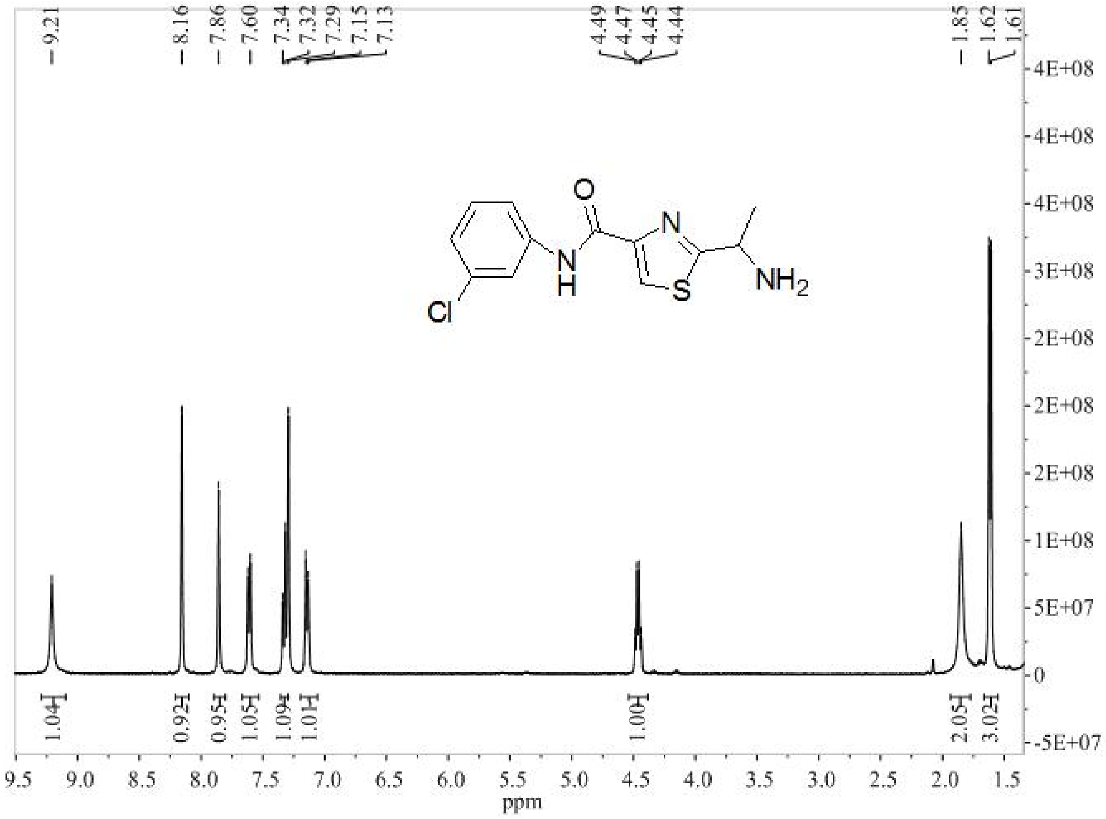

9.21
8.16
7.86
7.60
7.34
7.32
7.29
7.15
7.13
4.49
4.47
4.45
4.44
1.85
1.62
1.61
O
N
H
N
S
NH2
Cl
1.04
0.92
0.95
1.05
1.09
1.01
1.00
2.05
3.02
ppm

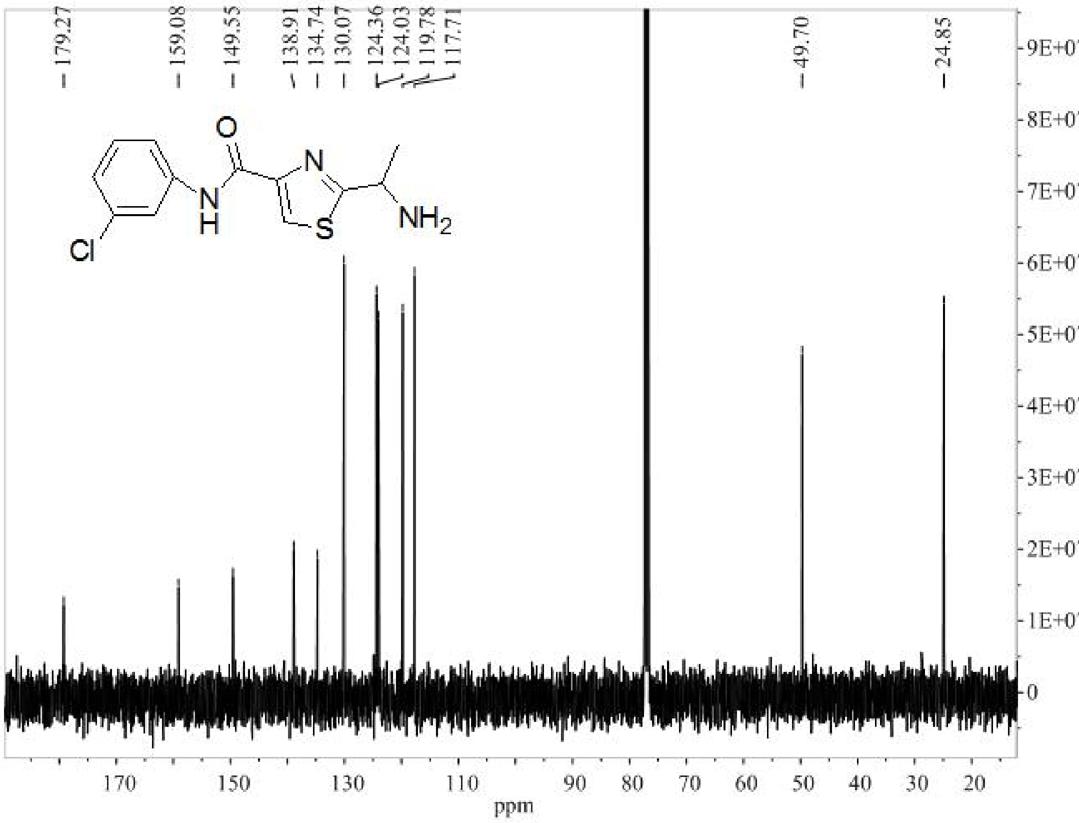
179.27
159.08
149.55
138.91
134.74
130.07
124.36
124.03
119.78
117.71
49.70
24.85
O
N
H
N
S
NH2
Cl
9E+07
8E+07
7E+07
6E+07
5E+07
4E+07
3E+07
2E+07
1E+07
0
170
150
130
110
90
80
70
60
50
40
30
20
ppm

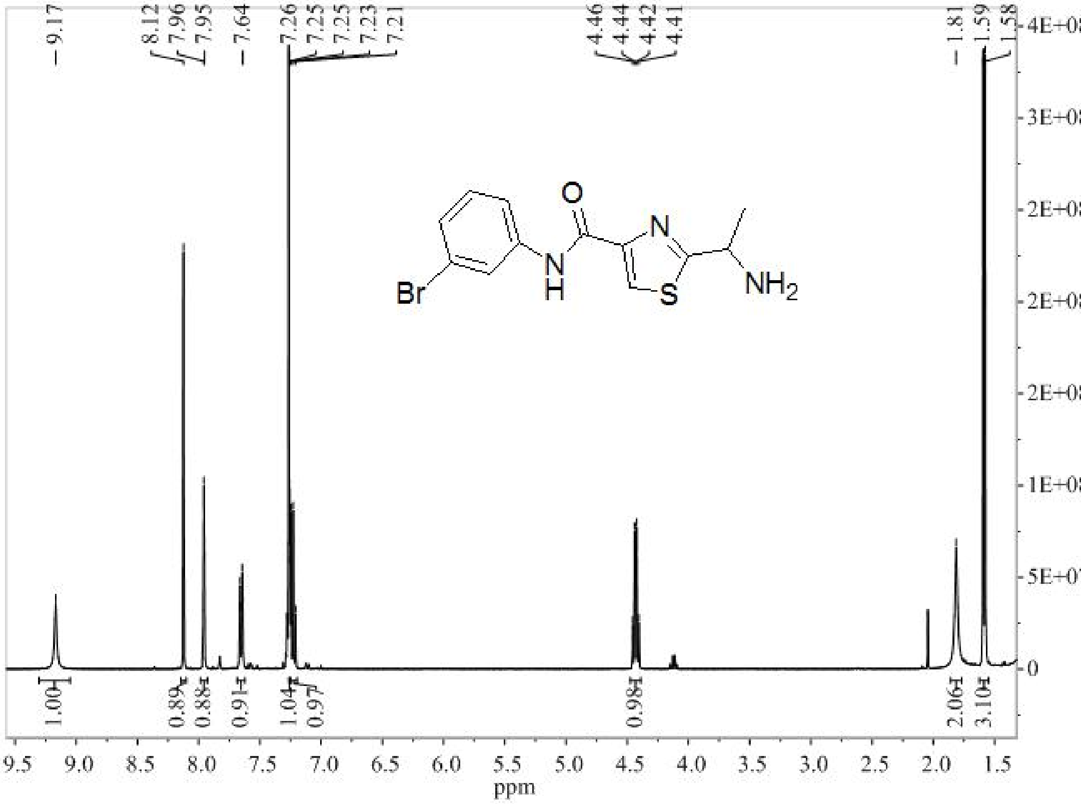
9.17
8.12
7.96
7.95
7.64
7.26
7.25
7.25
7.23
7.21
4.46
4.44
4.42
4.41
1.81
1.59
1.58
O
N
H
N
S
NH2
Br
1.00
0.89
0.88
0.91
1.04
0.97
0.98
2.06
3.10
4E+08
3E+08
2E+08
2E+08
2E+08
1E+08
5E+07
0
9.5
9.0
8.5
8.0
7.5
7.0
6.5
6.0
5.5
5.0
4.5
4.0
3.5
3.0
2.5
2.0
1.5
ppm

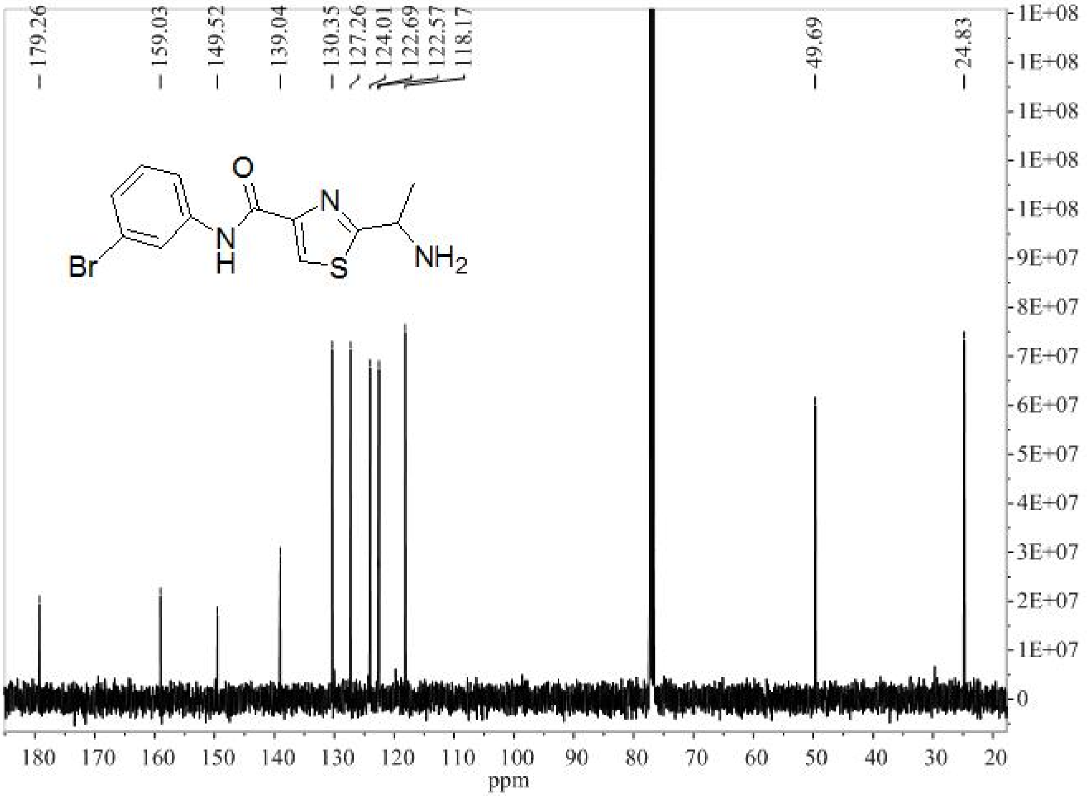

179.26
159.03
149.52
139.04
130.35
127.26
124.01
122.69
122.57
118.17
49.69
24.83
Br
O
N
H
N
S
NH2
1E+08
1E+08
1E+08
1E+08
1E+08
9E+07
8E+07
7E+07
6E+07
5E+07
4E+07
3E+07
2E+07
1E+07
0
180 170 160 150 140 130 120 110 100 90 80 70 60 50 40 30 20
ppm

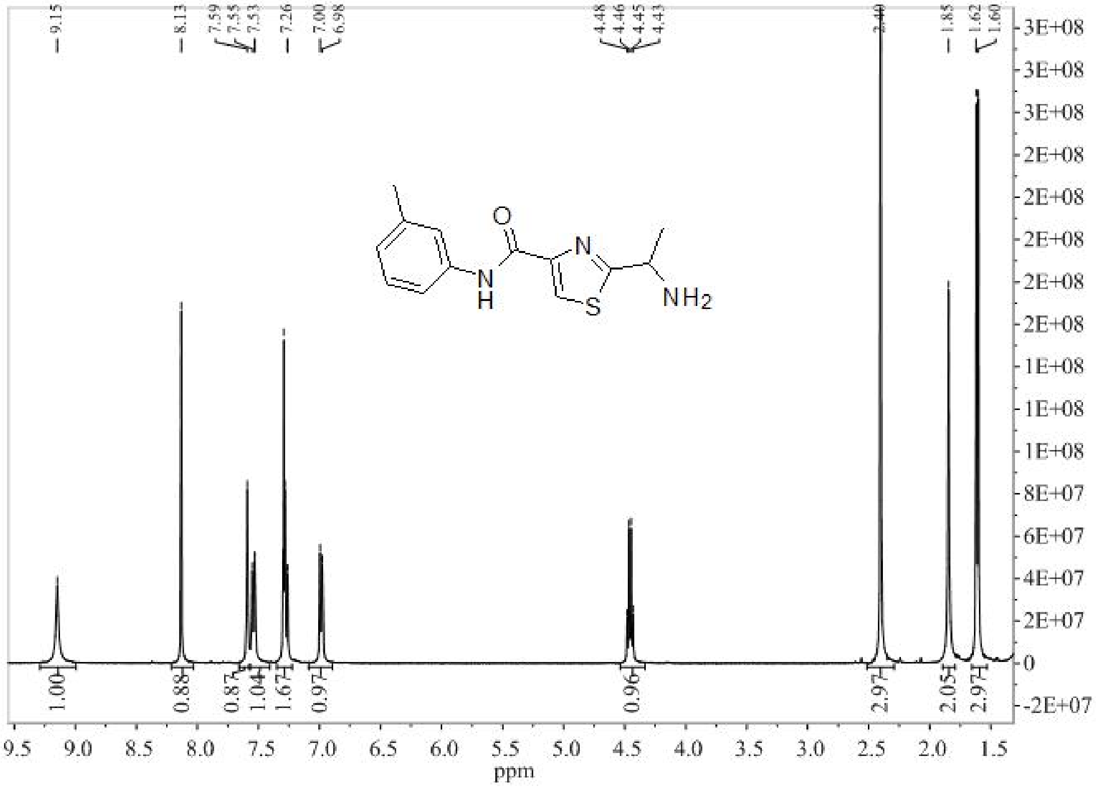
9.15
8.13
7.59
7.55
7.53
7.26
7.00
6.98
4.48
4.46
4.45
4.43
2.40
1.85
1.62
1.60
O
N
N
H
S
NH2
1.00
0.88
0.87
1.04
1.67
0.97
0.96
2.97
2.05
2.97
ppm

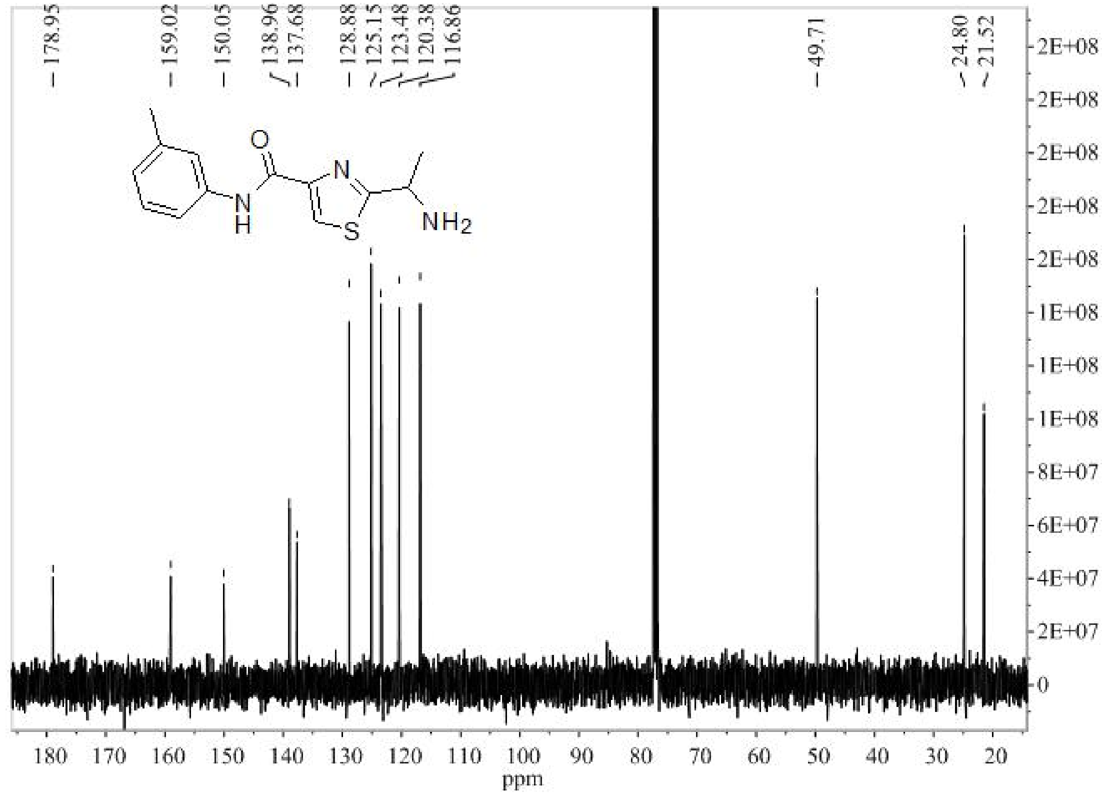
178.95
159.02
150.05
138.96
137.68
128.88
125.15
123.48
120.38
116.86
49.71
24.80
21.52
O
N
N
H
S
NH2
ppm

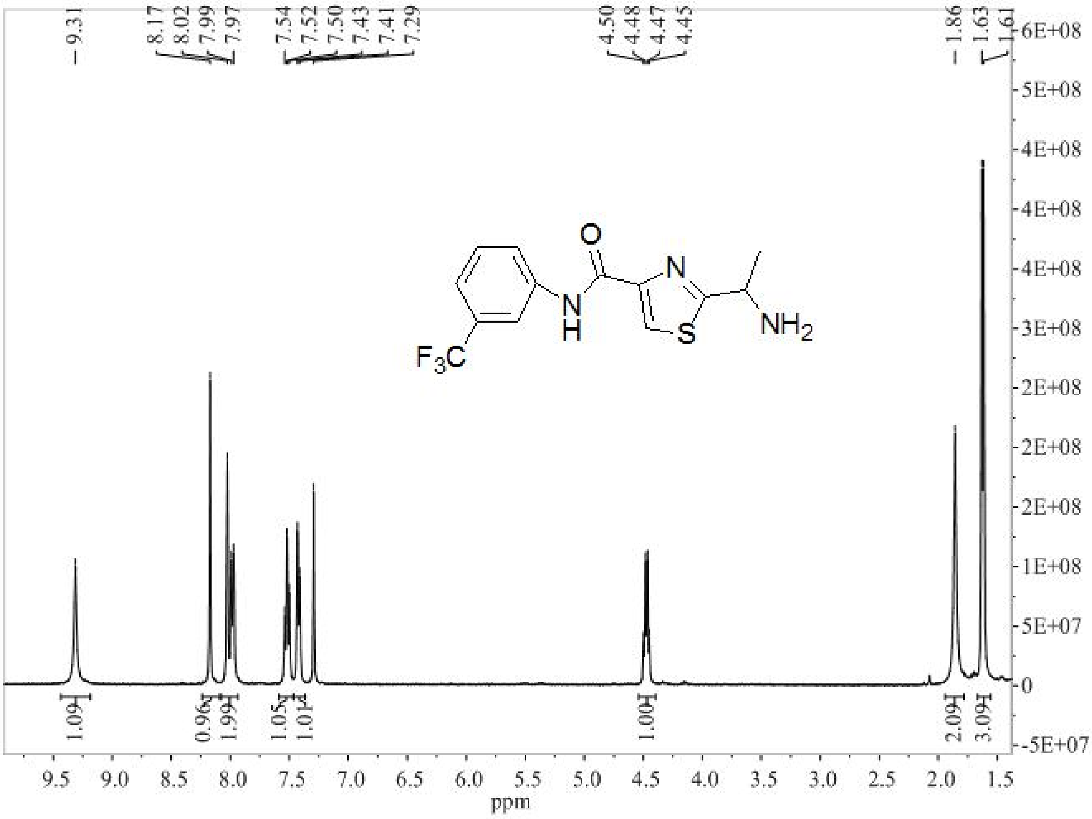
9.31
8.17
8.02
7.99
7.97
7.54
7.52
7.50
7.43
7.41
7.29
4.50
4.48
4.47
4.45
1.86
1.63
1.61
O
N
H
N
S
NH2
F3C
1.09
0.96
1.99
1.05
1.01
1.00
2.09
3.09
6E+08
5E+08
4E+08
4E+08
4E+08
3E+08
2E+08
2E+08
2E+08
1E+08
5E+07
0
-5E+07
9.5 9.0 8.5 8.0 7.5 7.0 6.5 6.0 5.5 5.0 4.5 4.0 3.5 3.0 2.5 2.0 1.5
ppm

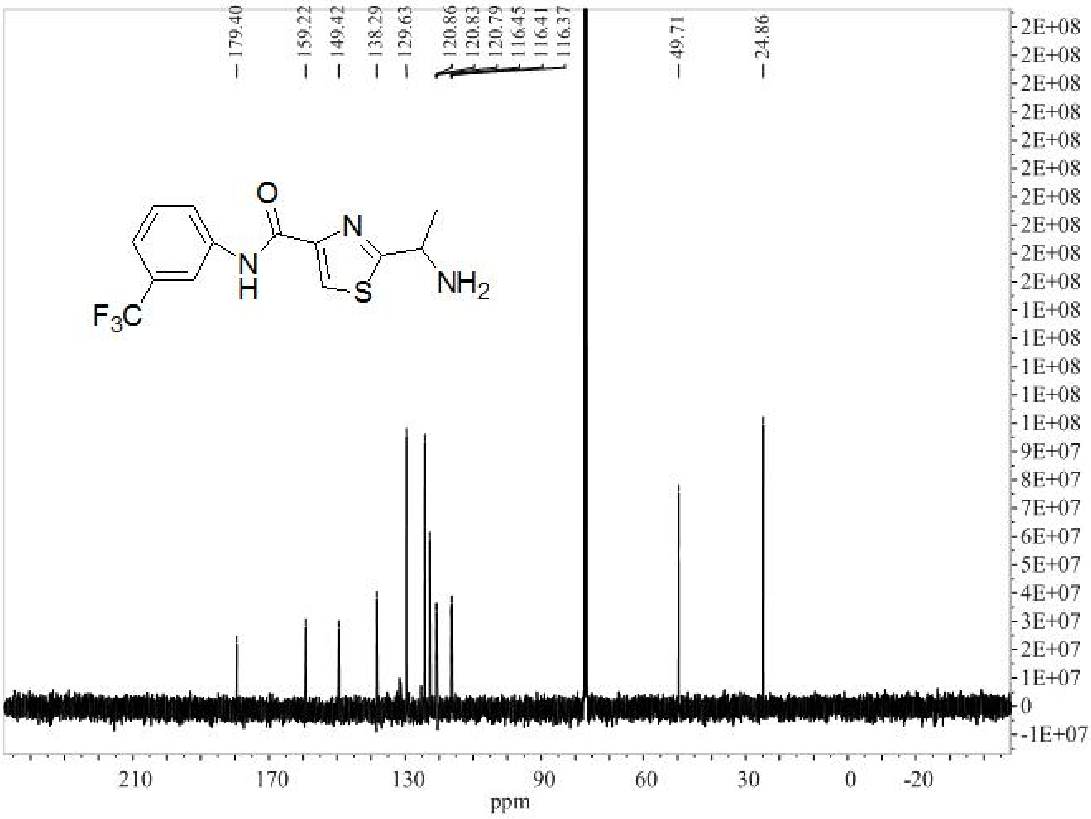
179.40
159.22
149.42
138.29
129.63
120.86
120.83
120.79
116.45
116.41
116.37
49.71
24.86
O
N
H
N
S
NH2
F3C
2E+08
1E+08
9E+07
8E+07
7E+07
6E+07
5E+07
4E+07
3E+07
2E+07
1E+07
0
-1E+07
210 170 130 90 60 30 0 -20
ppm

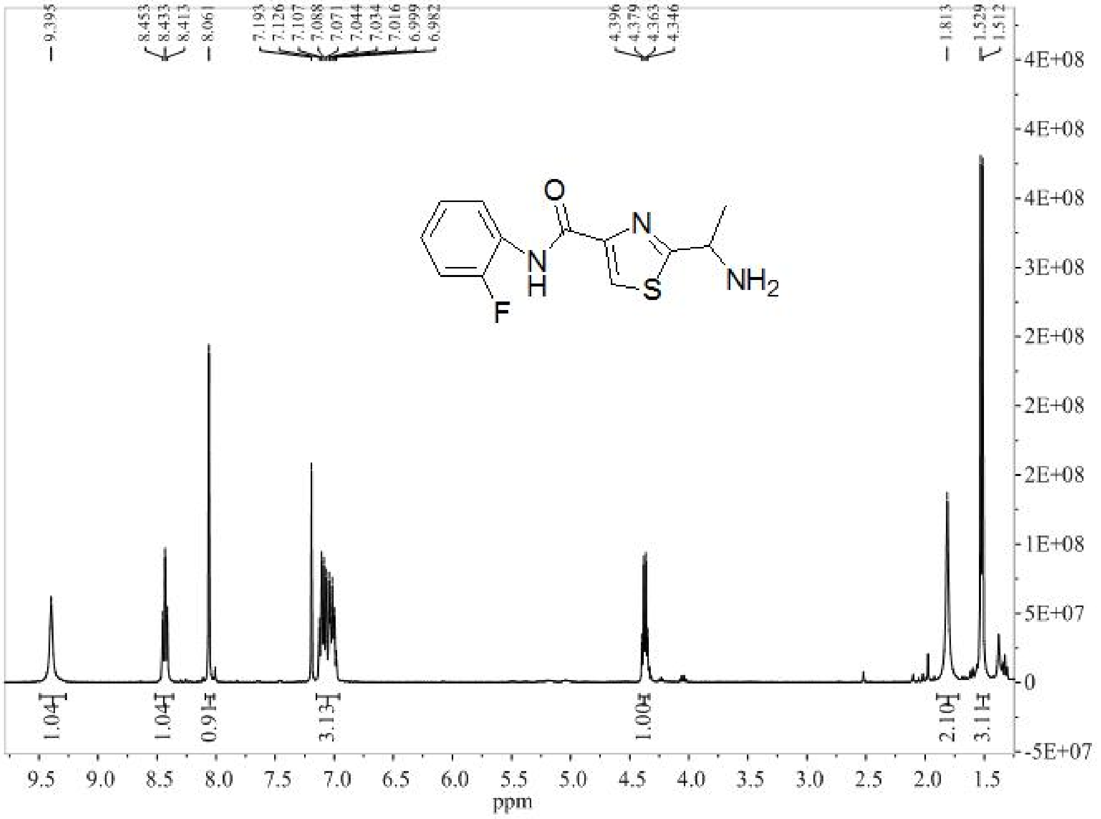

9.395
8.453
8.433
8.413
8.061
7.193
7.126
7.107
7.088
7.071
7.044
7.034
7.016
6.999
6.982
4.396
4.379
4.363
4.346
1.813
1.529
1.512
1.04
1.04
0.91
3.13
1.00
2.10
3.1
ppm

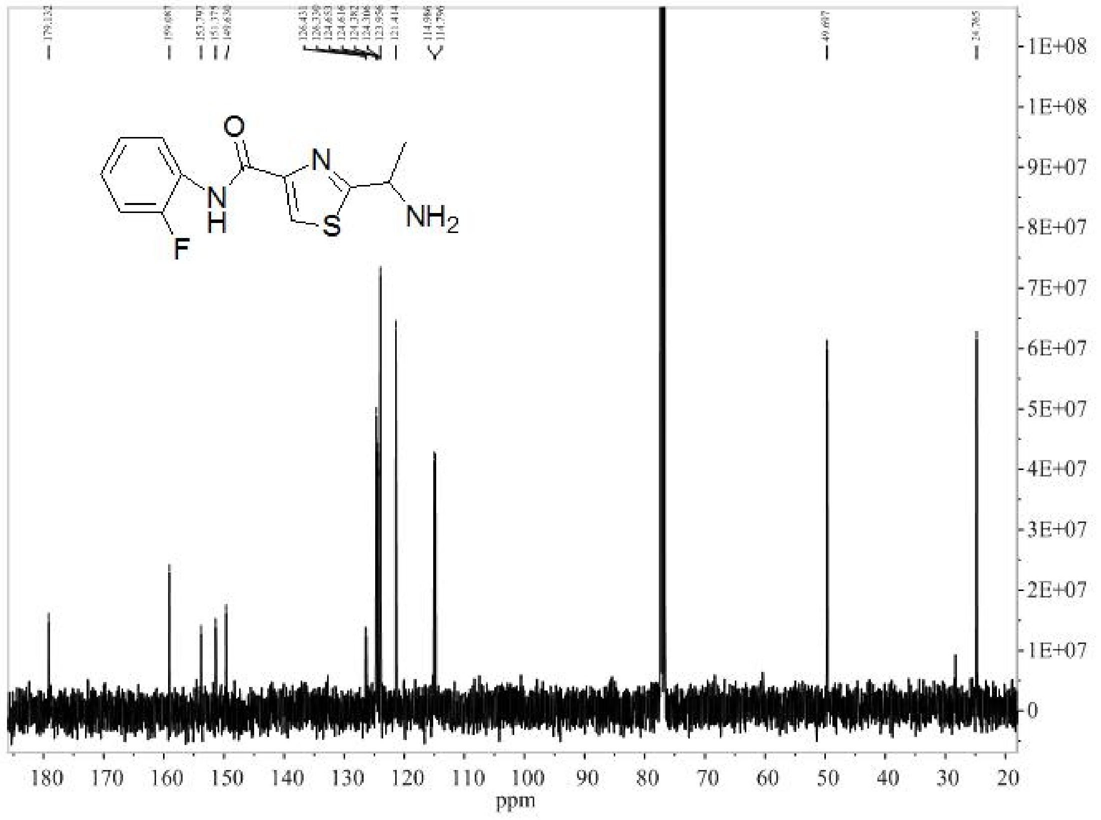

ppm

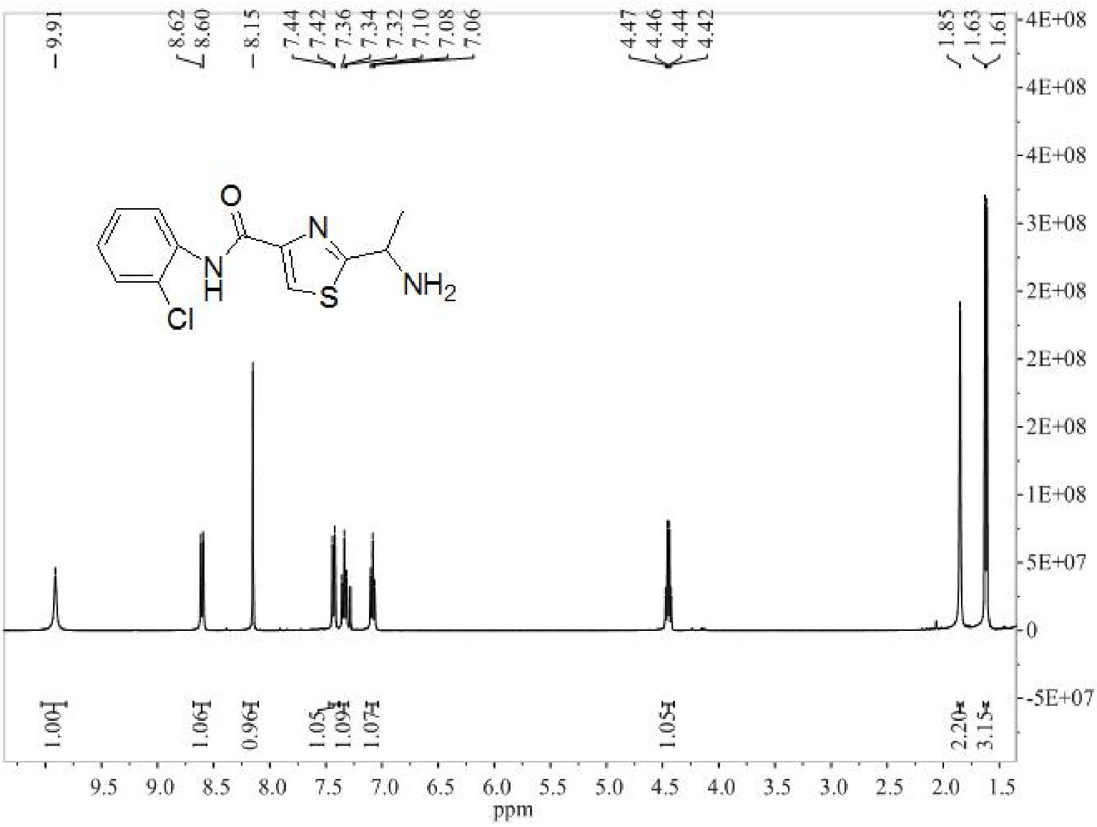
9.91
8.62
8.60
8.15
7.44
7.42
7.36
7.34
7.32
7.10
7.08
7.06
4.47
4.46
4.44
4.42
1.85
1.63
1.61
O
N
N
H
S
NH2
Cl
1.00
1.06
0.96
1.05
1.09
1.07
1.05
2.20
3.15
ppm

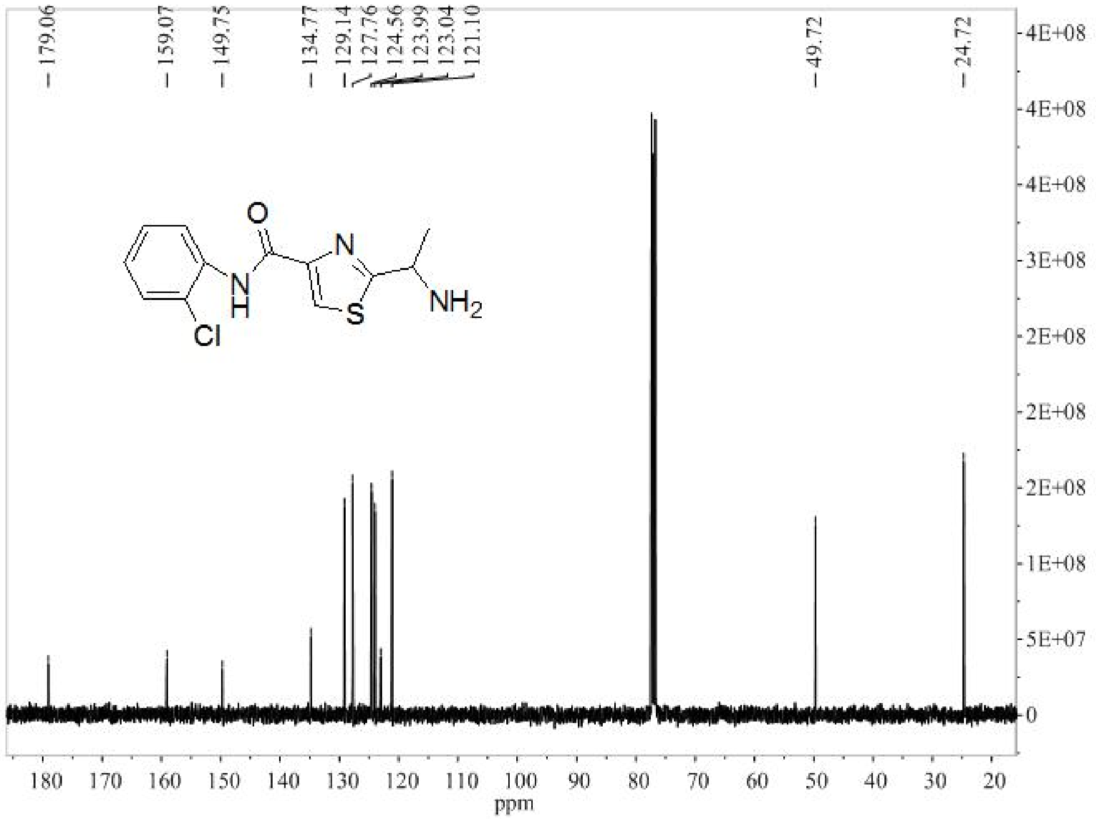
179.06
159.07
149.75
134.77
129.14
127.76
124.56
123.99
123.04
121.10
49.72
24.72
O
N
N
H
S
NH2
Cl
ppm

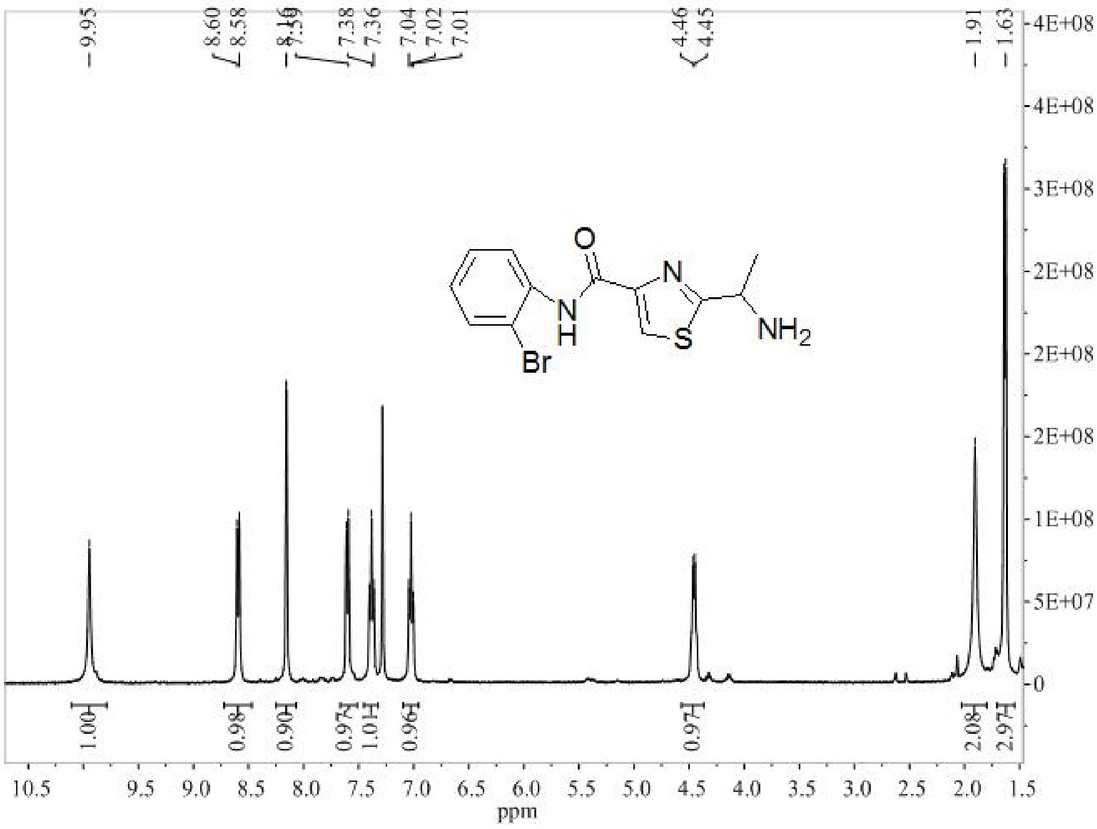
9.95
8.60
8.58
8.16
7.59
7.38
7.36
7.04
7.02
7.01
4.46
4.45
1.91
1.63
O
N
H
S
NH2
Br
1.00
0.98
0.90
0.97
1.01
0.96
0.97
2.08
2.97
ppm

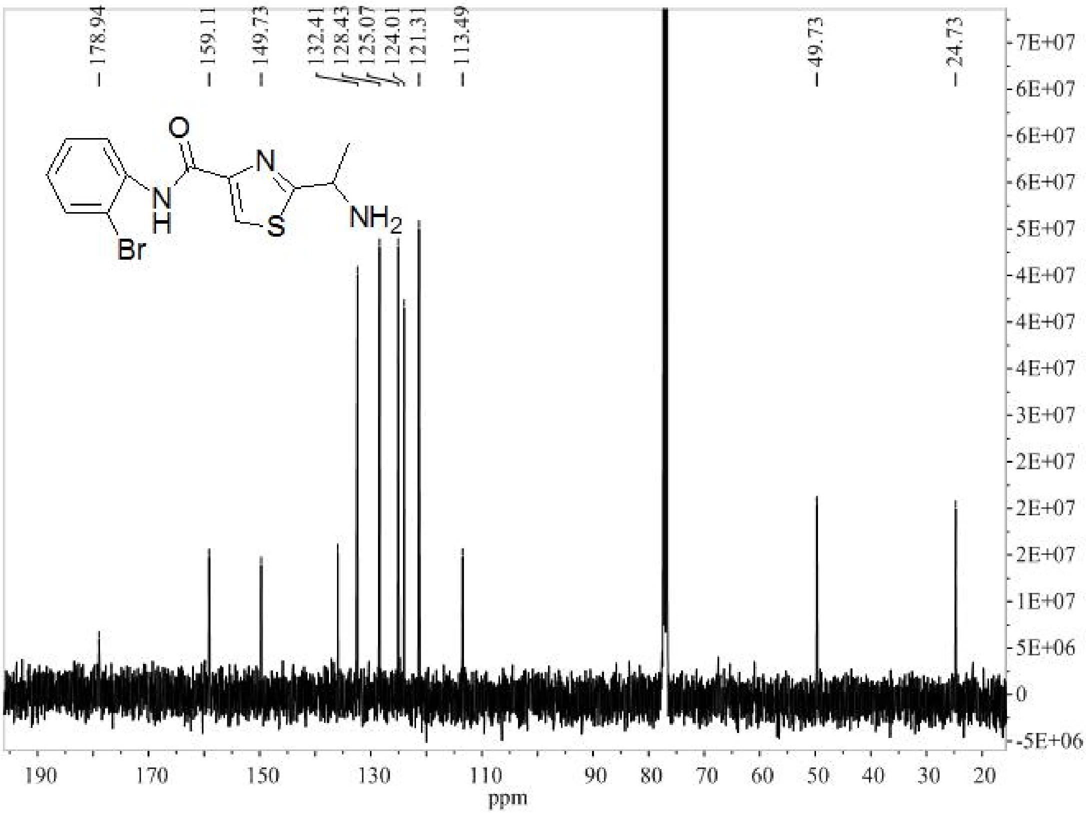
178.94
159.11
149.73
132.41
128.43
125.07
124.01
121.31
113.49
49.73
24.73
O
N
H
S
NH2
Br
ppm

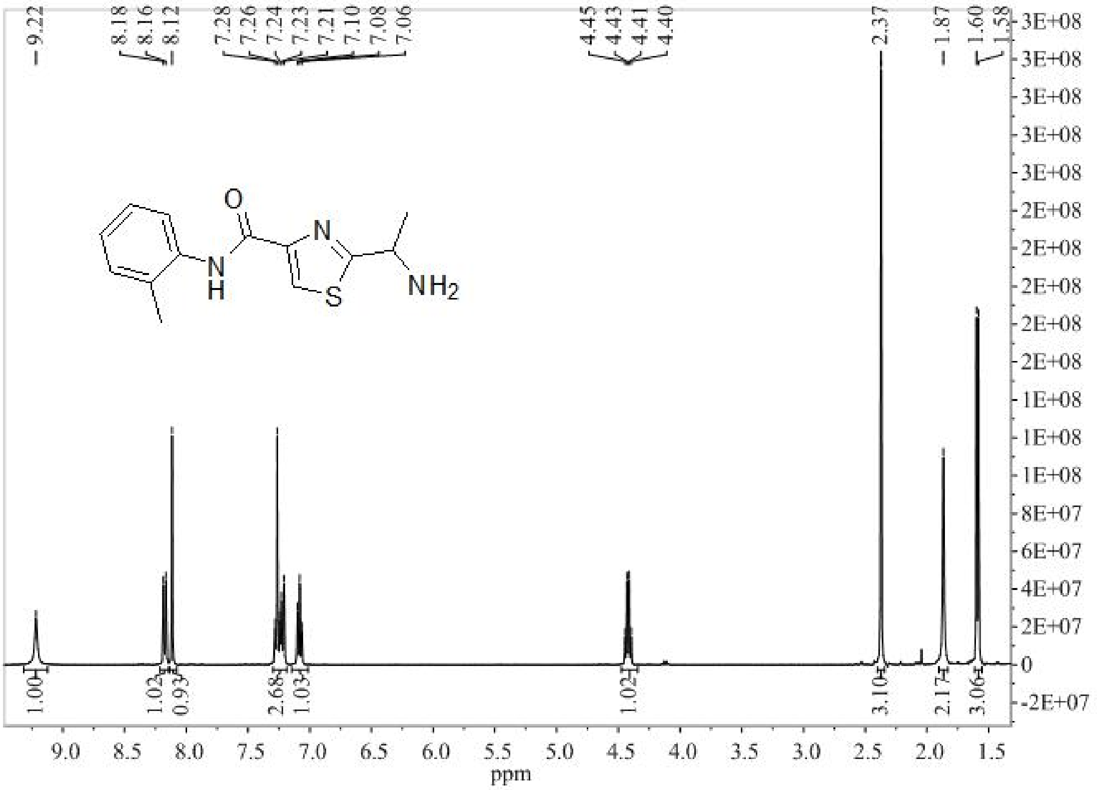
9.22
8.18
8.16
8.12
7.28
7.26
7.24
7.23
7.21
7.10
7.08
7.06
4.45
4.43
4.41
4.40
2.37
1.87
1.60
1.58
O
N
H
N
S
NH2
1.00
1.02
0.93
2.68
1.03
1.02
3.10
2.17
3.06
ppm

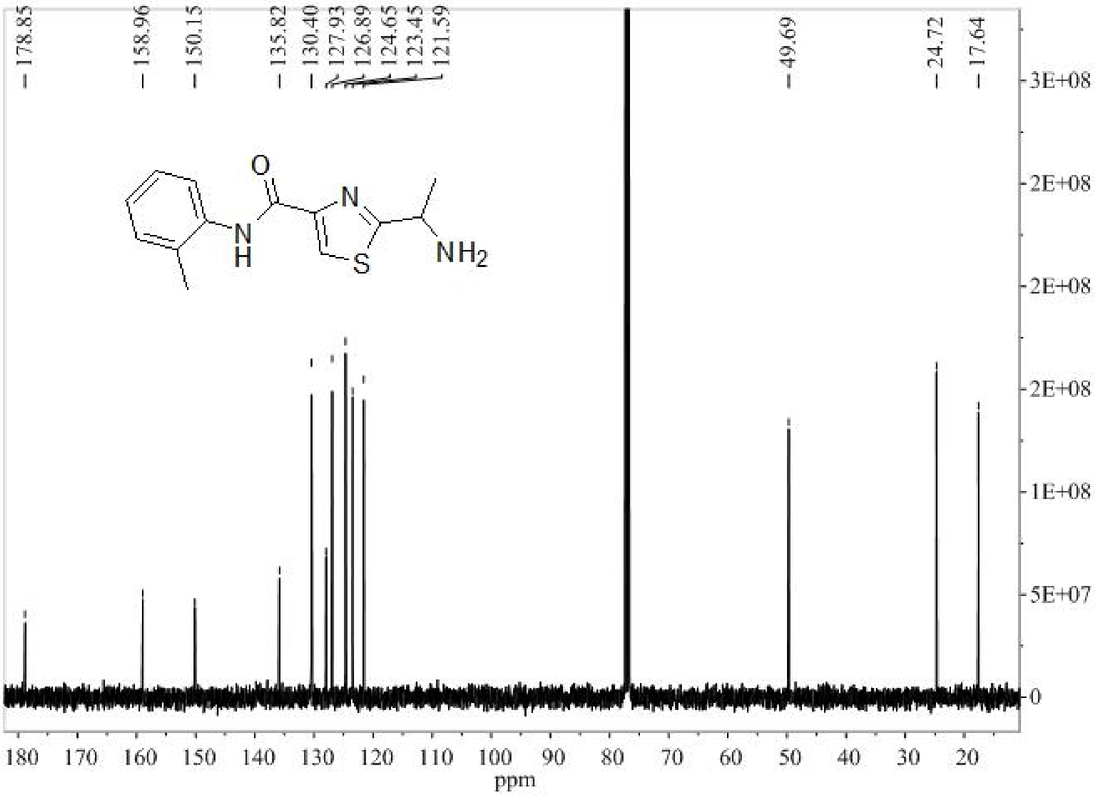
178.85
158.96
150.15
135.82
130.40
127.93
126.89
124.65
123.45
121.59
49.69
24.72
17.64
O
N
H
N
S
NH2
ppm

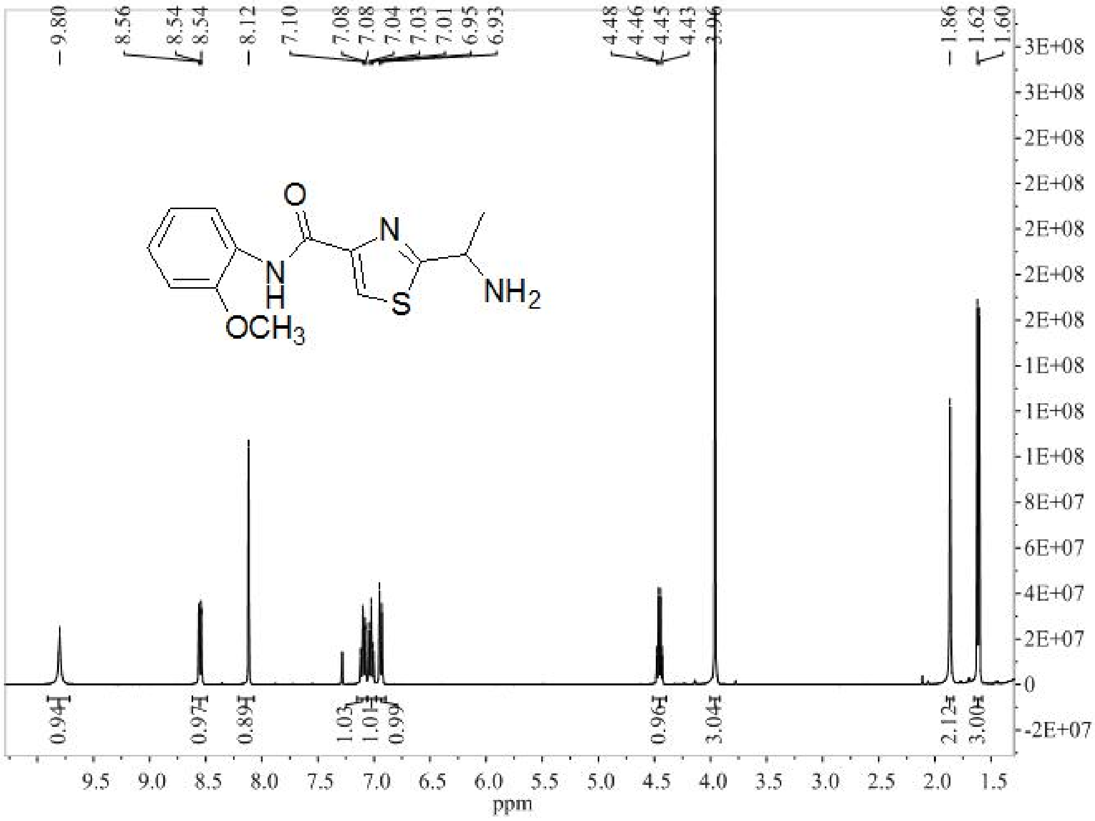

9.80
8.56
8.54
8.54
8.12
7.10
7.08
7.08
7.04
7.03
7.01
6.95
6.93
4.48
4.46
4.45
4.43
3.96
1.86
1.62
1.60
O
N
H
N
S
NH2
OCH3
0.94
0.97
0.89
1.03
1.01
0.99
0.96
3.04
2.12
3.00
ppm

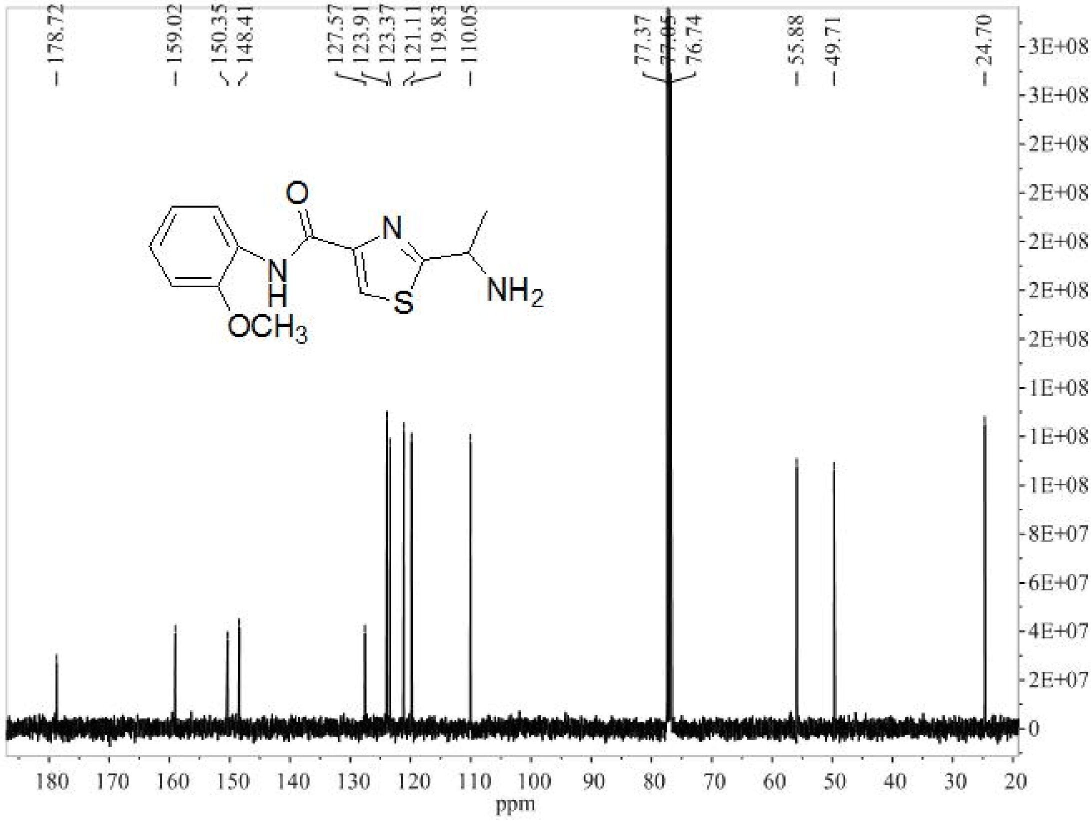

178.72
159.02
150.35
148.41
127.57
123.91
123.37
121.11
119.83
110.05
77.37
77.05
76.74
55.88
49.71
24.70
O
N
H
N
S
NH2
OCH3
ppm

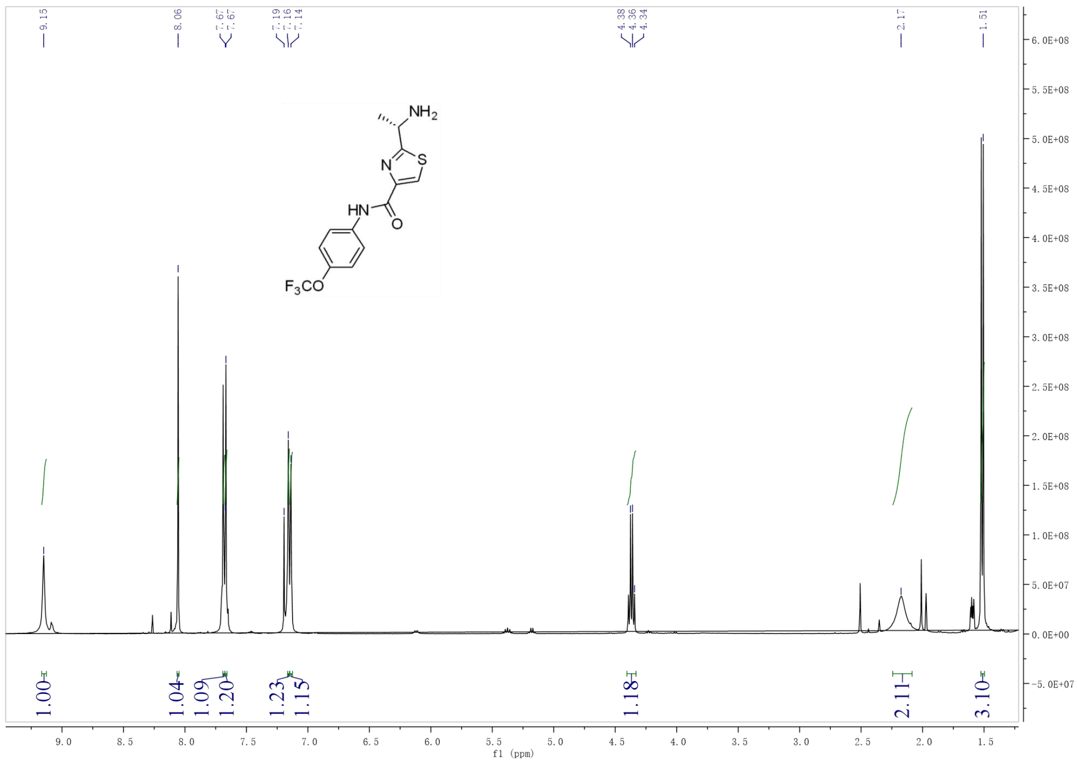
9.15
8.06
7.67
7.67
7.19
7.16
7.14
4.38
4.36
4.34
2.17
1.51
NH2
N
S
HN
O
F3CO
1.00
1.04
1.09
1.20
1.23
1.15
1.18
2.11
3.10
f1 (ppm)

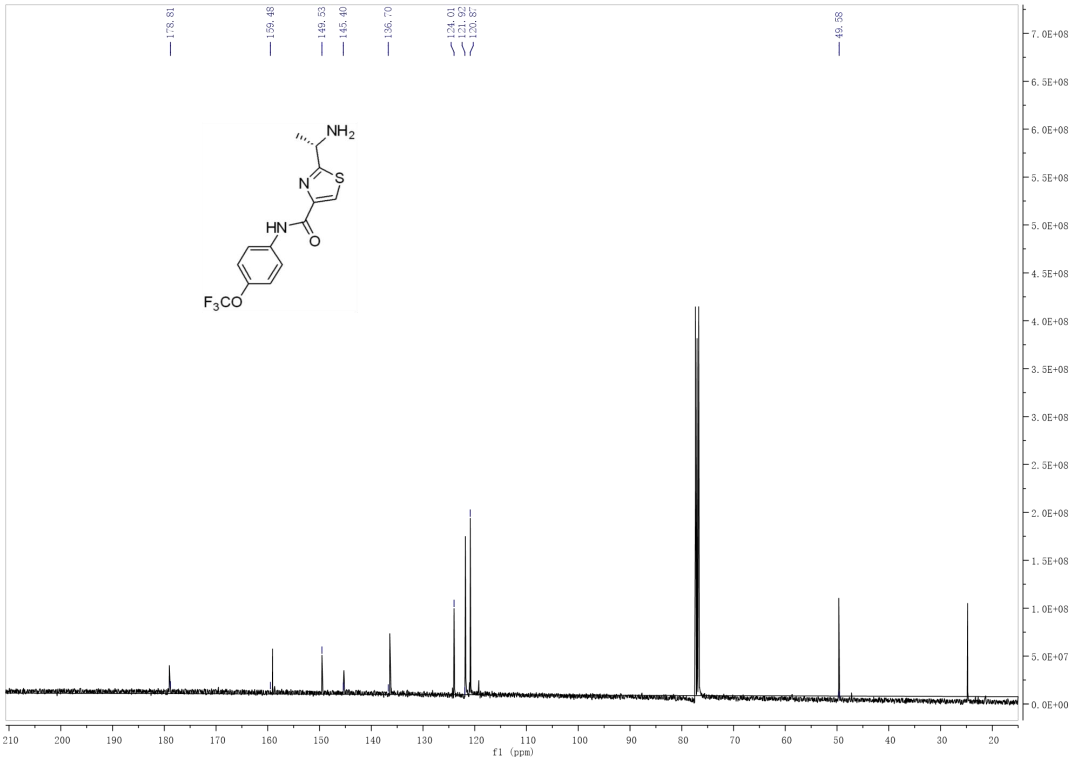
178.81
159.48
149.53
145.40
136.70
124.01
121.92
120.87
49.58
NH2
N
S
HN
O
F3CO
f1 (ppm)

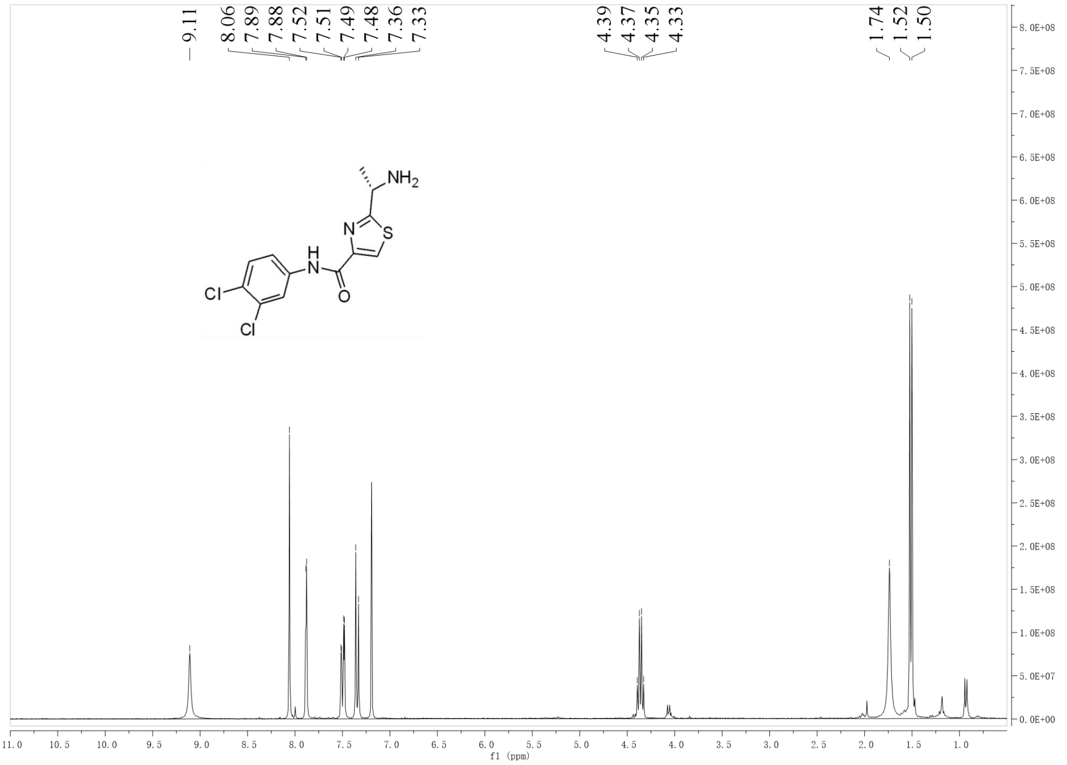
9.11
8.06
7.89
7.88
7.52
7.51
7.49
7.48
7.36
7.33
4.39
4.37
4.35
4.33
1.74
1.52
1.50
NH2
N
S
H
N
O
Cl
Cl
f1 (ppm)

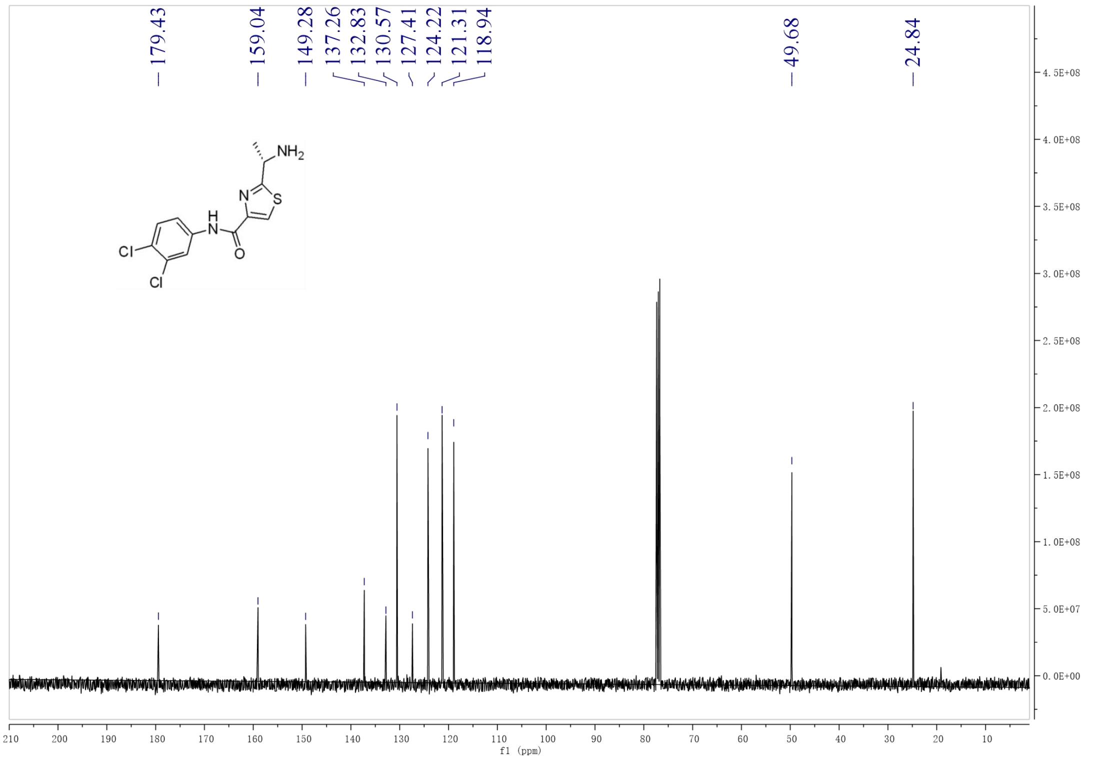
179.43
159.04
149.28
137.26
132.83
130.57
127.41
124.22
121.31
118.94
49.68
24.84
NH2
N
S
H
N
O
Cl
Cl
f1 (ppm)

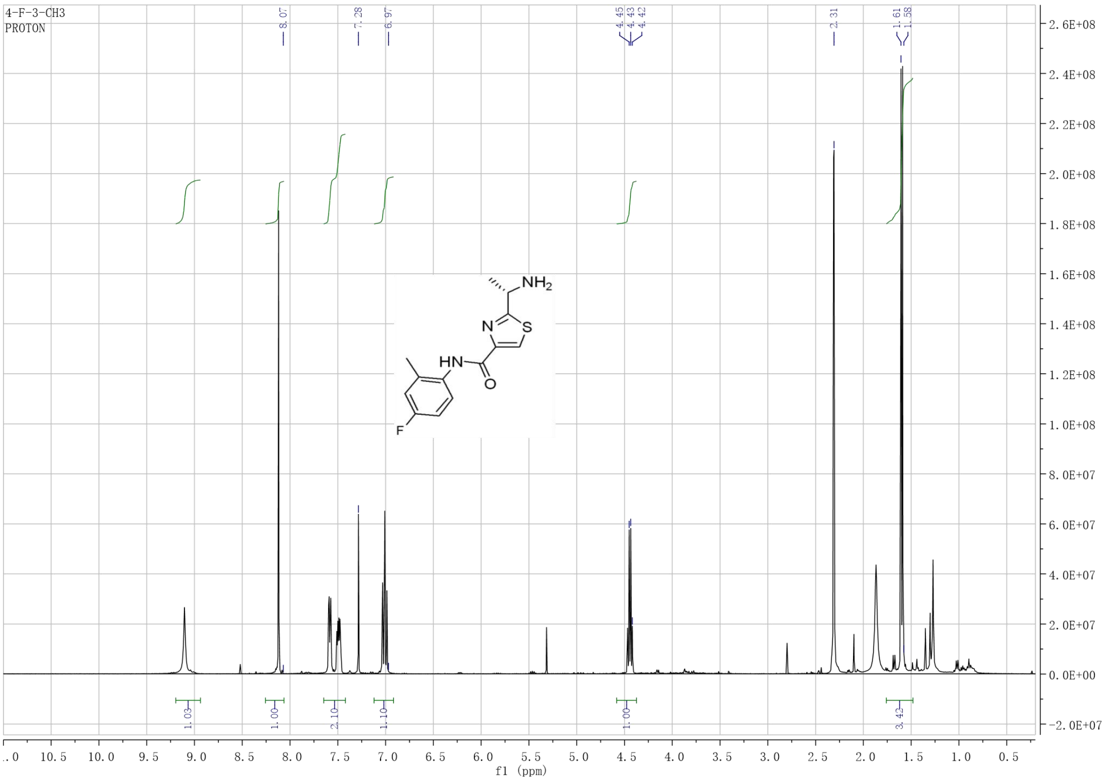
4-F-3-CH3
PROTON
8.07
7.28
6.97
4.45
4.43
4.42
2.31
1.61
1.58
NH2
N
S
HN
O
F
1.03
1.00
2.10
1.10
1.00
3.42
f1 (ppm)

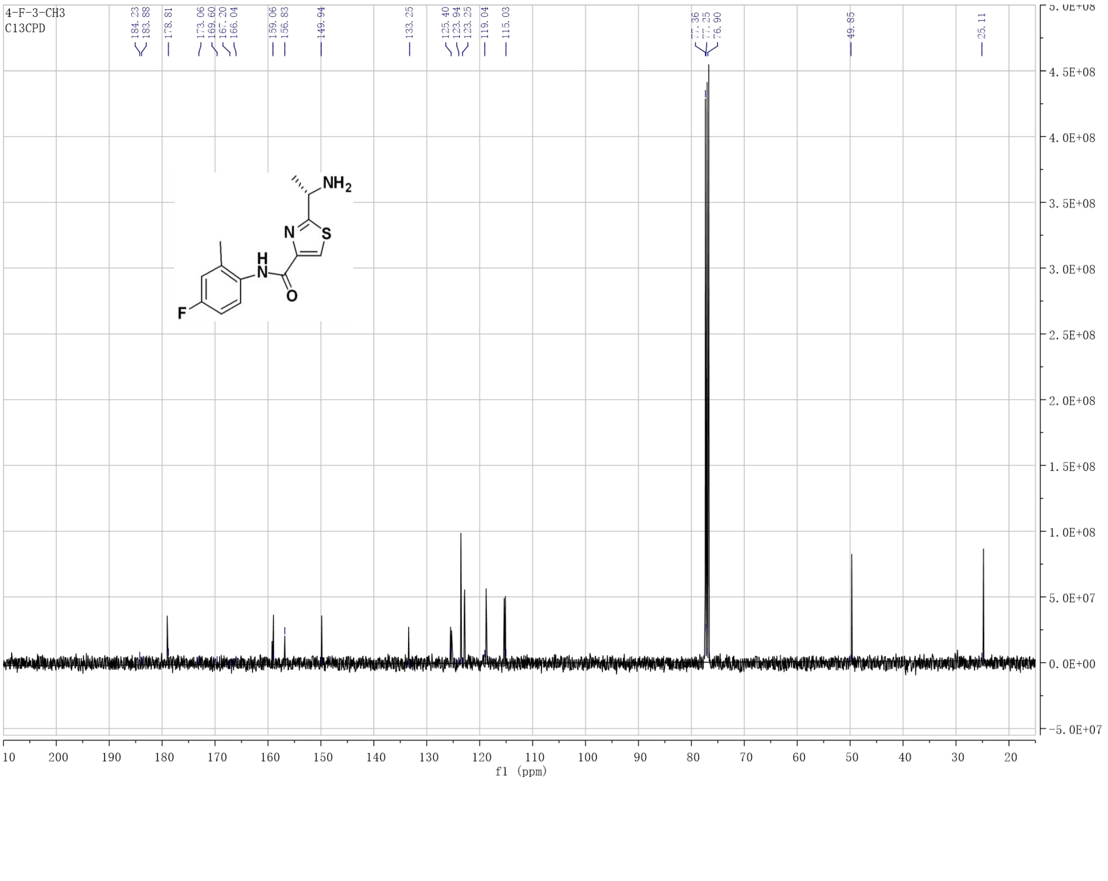
4-F-3-CH3
C13CPD
184.23
183.88
178.81
173.06
169.60
167.20
166.04
159.06
156.83
149.94
133.25
125.40
123.94
123.25
119.04
115.03
77.36
77.25
76.90
49.85
25.11
NH2
N
S
H
N
O
F
f1 (ppm)

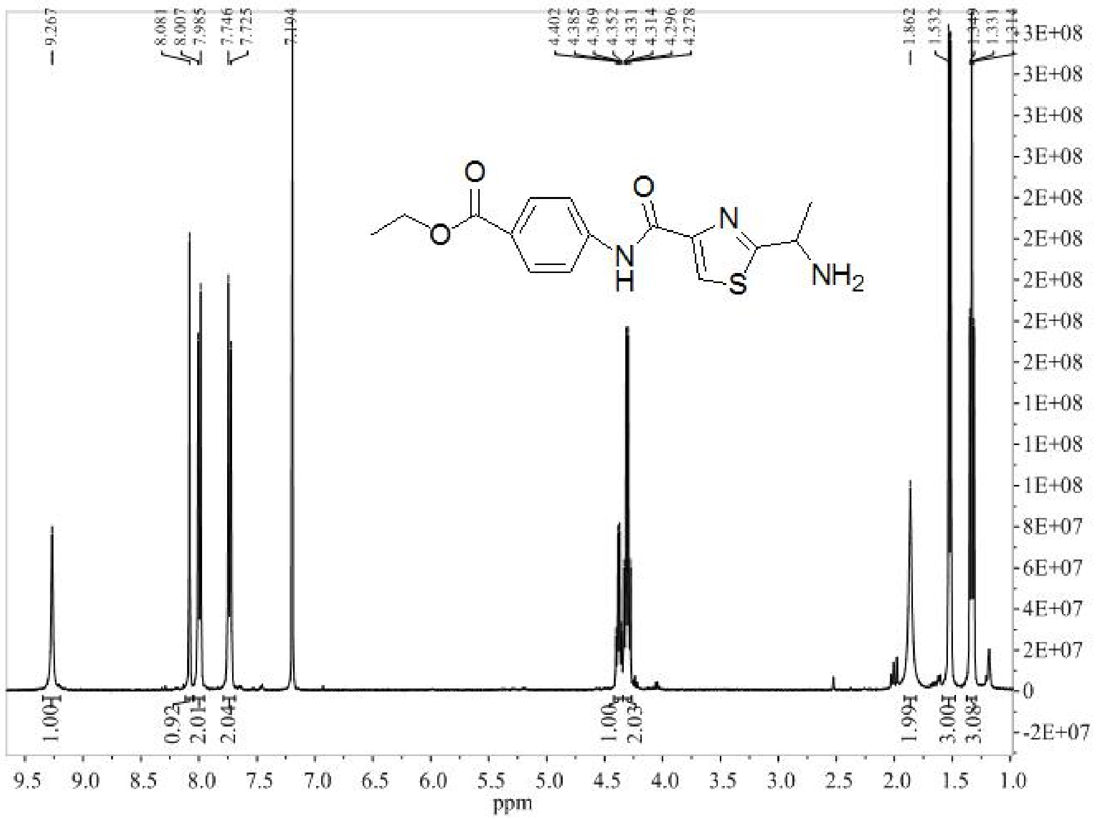

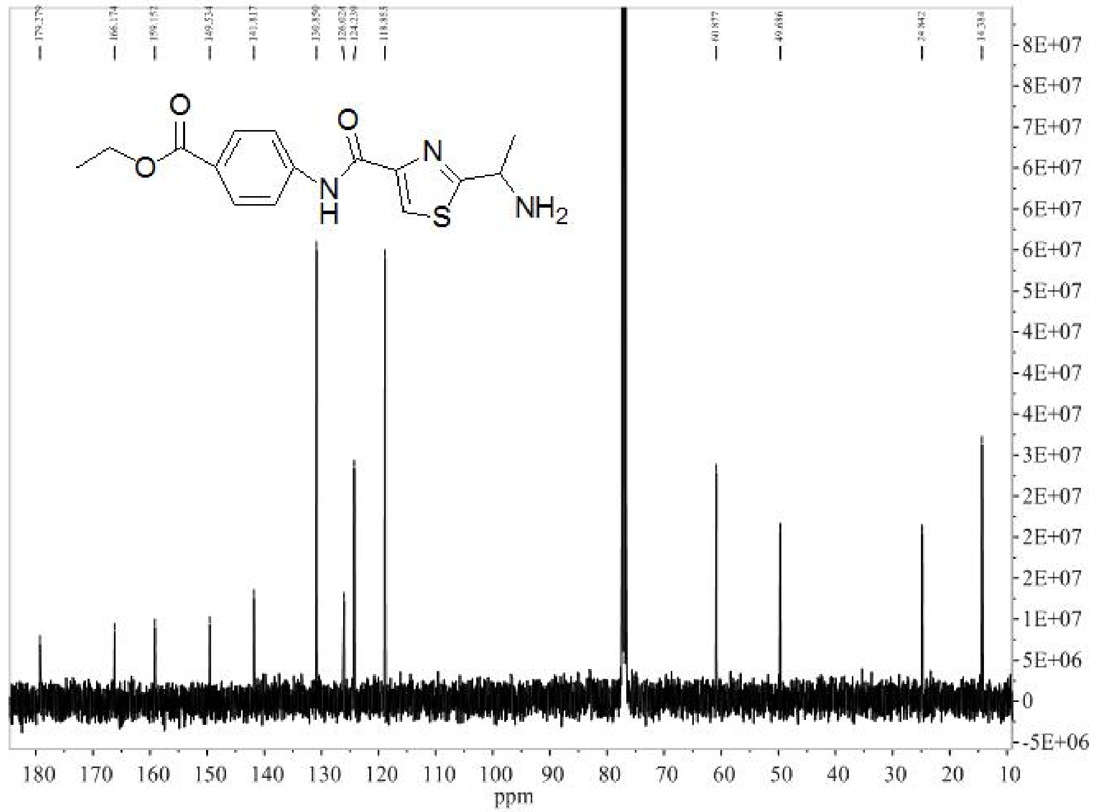

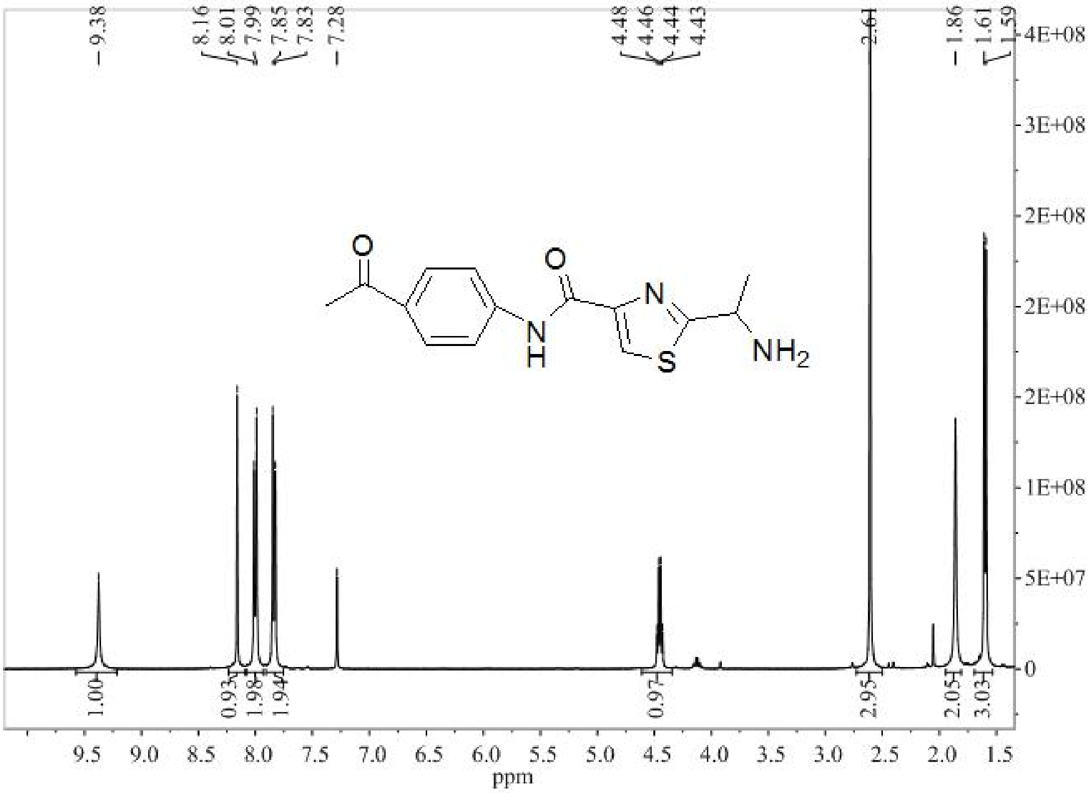
9.38
8.16
8.01
7.99
7.85
7.83
7.28
4.48
4.46
4.44
4.43
2.61
1.86
1.61
1.59
O
O
N
N
H
S
NH2
1.00
0.93
1.98
1.94
0.97
2.95
2.05
3.03
ppm

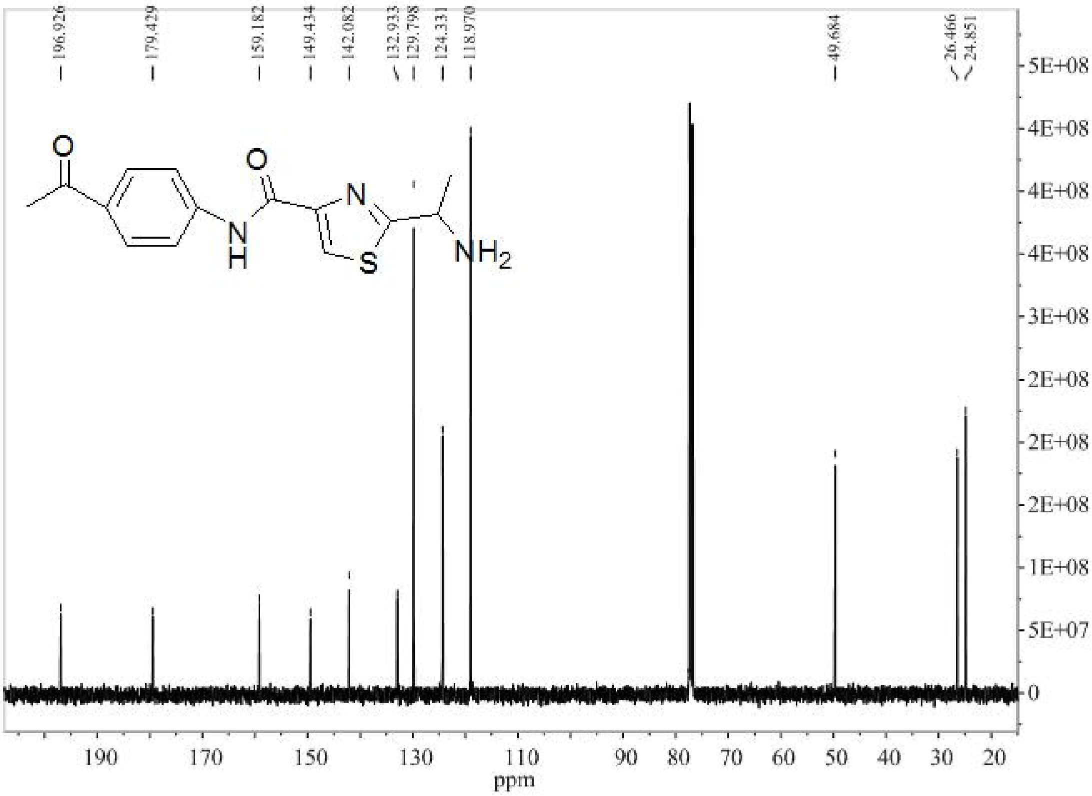
196.926
179.429
159.182
149.434
142.082
132.933
129.798
124.331
118.970
49.684
26.466
24.851
O
O
N
N
H
S
NH2
ppm

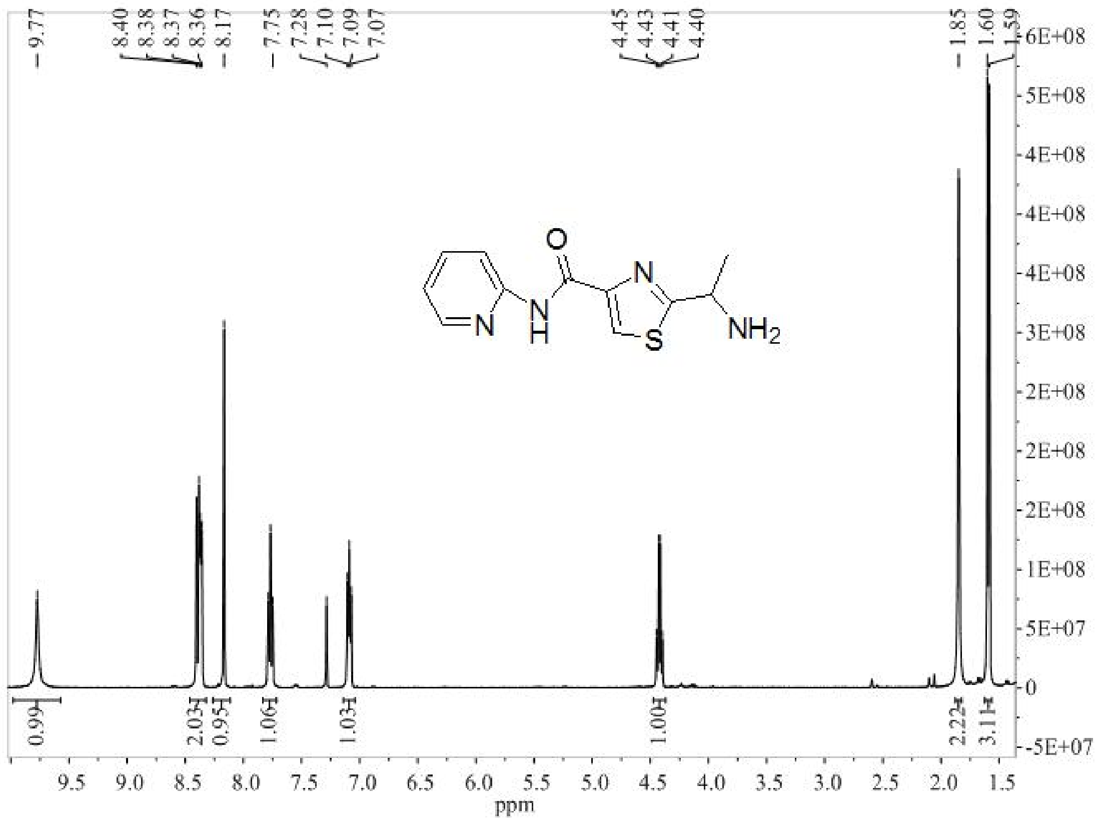
9.77
8.40
8.38
8.37
8.36
8.17
7.75
7.28
7.10
7.09
7.07
4.45
4.43
4.41
4.40
1.85
1.60
1.59
O
N
N
H
N
S
NH2
0.99
2.03
0.95
1.06
1.03
1.00
2.22
3.11
ppm

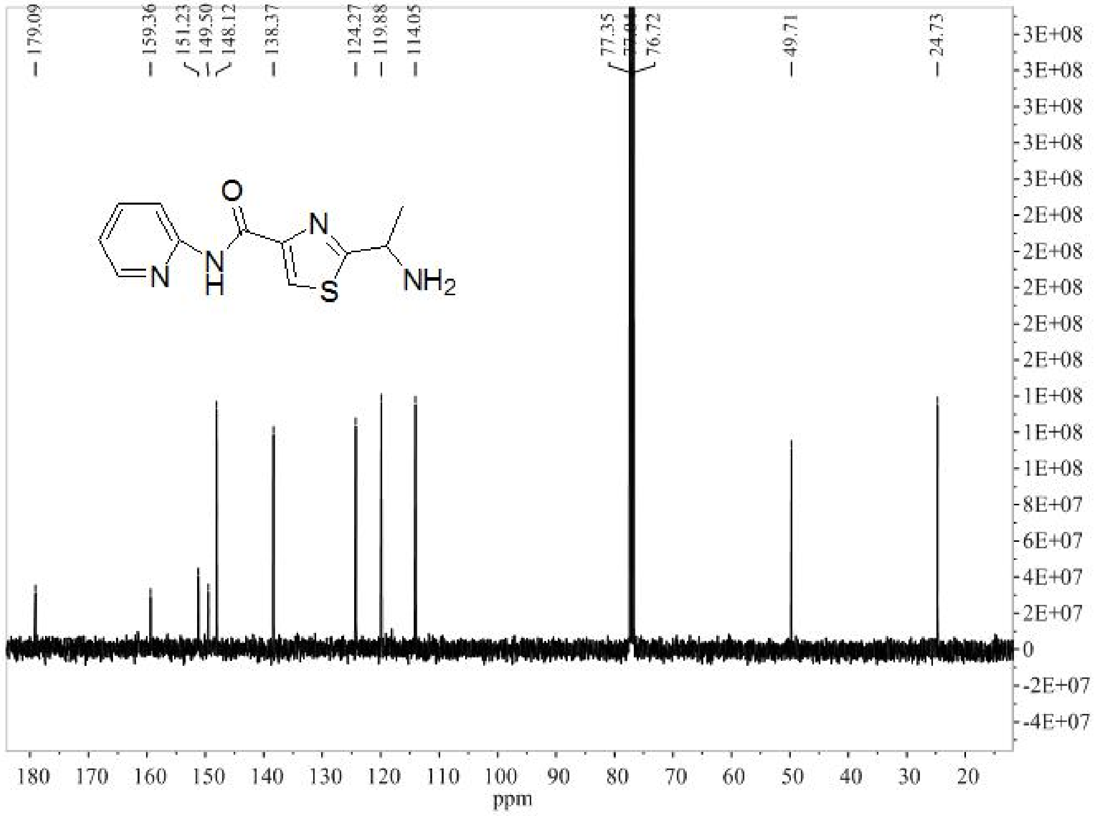
179.09
159.36
151.23
149.50
148.12
138.37
124.27
119.88
114.05
77.35
76.72
49.71
24.73
O
N
N
H
N
S
NH2
ppm

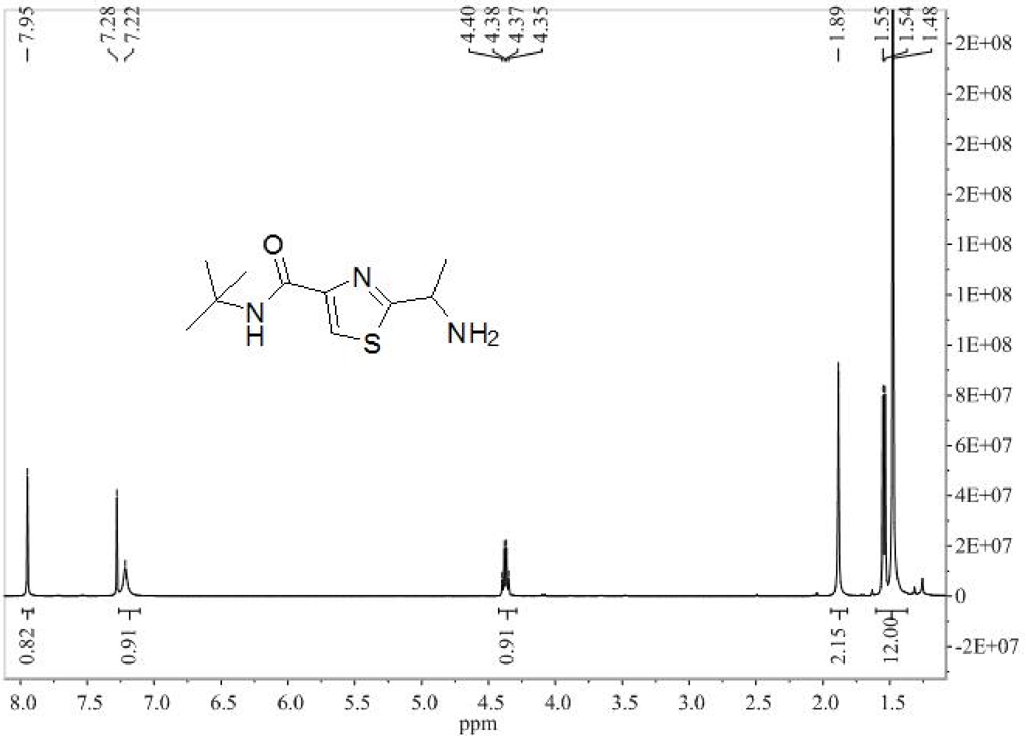

7.95
7.28
7.22
4.40
4.38
4.37
4.35
1.89
1.55
1.54
1.48
O
N
N
H
S
NH2
0.82
0.91
0.91
2.15
12.00
ppm

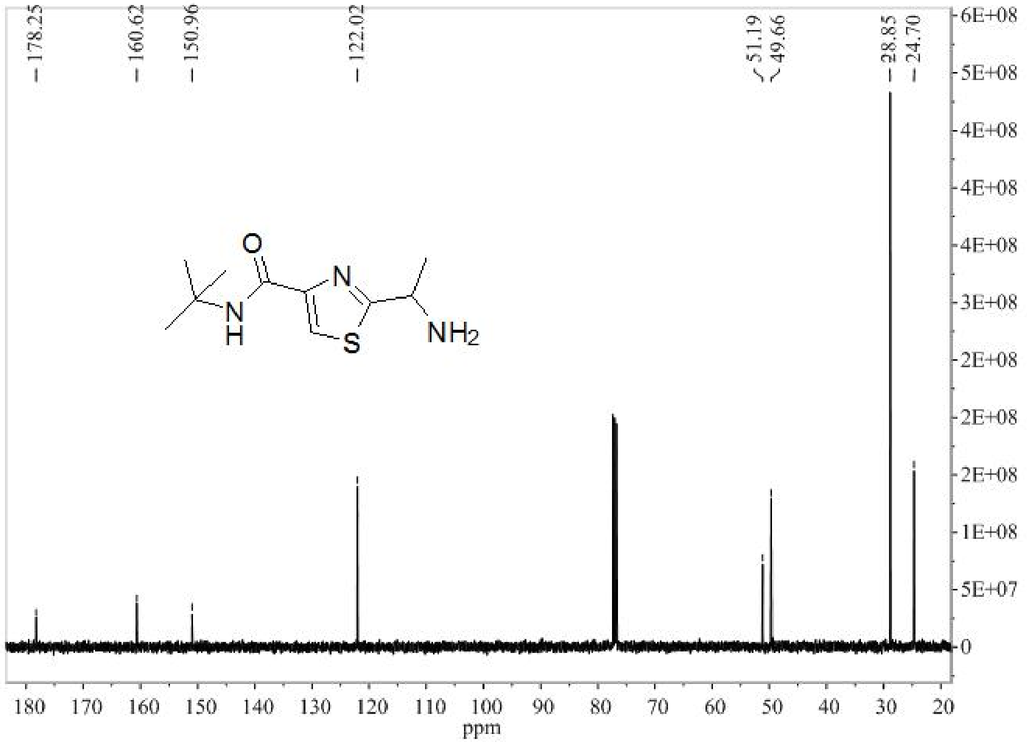

178.25
160.62
150.96
122.02
51.19
49.66
28.85
24.70
O
N
N
H
S
NH2
ppm

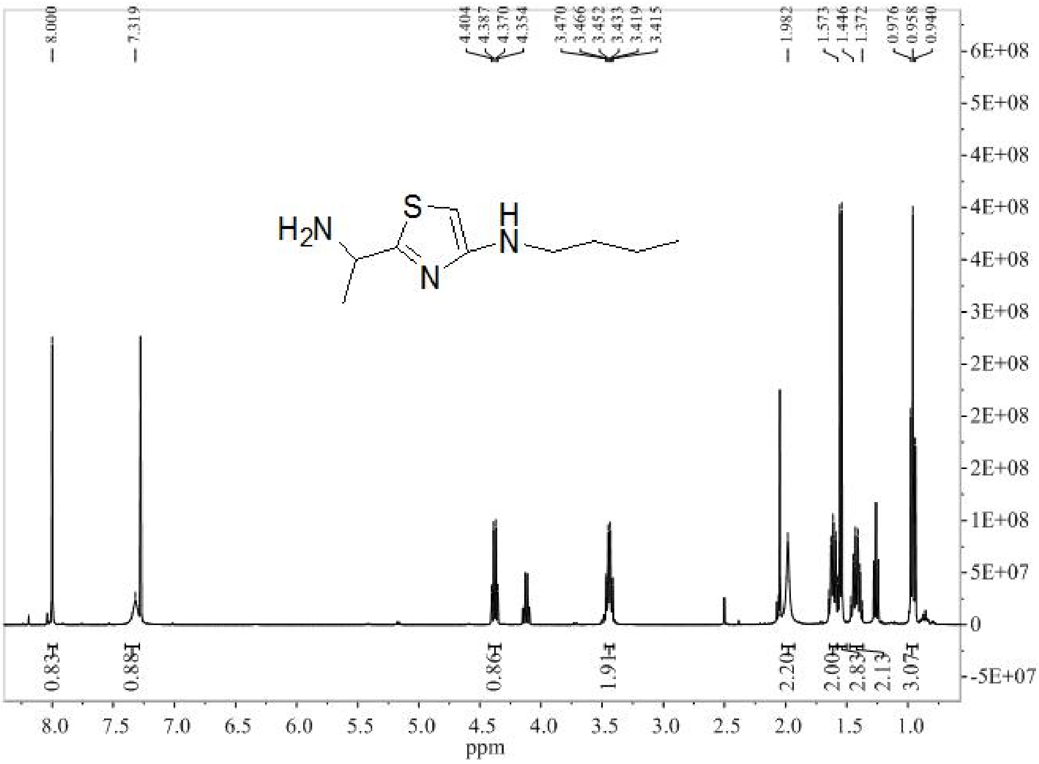
8.000
7.319
4.404
4.387
4.370
4.354
3.470
3.466
3.452
3.433
3.419
3.415
1.982
1.573
1.446
1.372
0.976
0.958
0.940
H2N
S
N
H
N
0.83
0.88
0.86
1.91
2.20
2.00
2.83
2.13
3.07
ppm

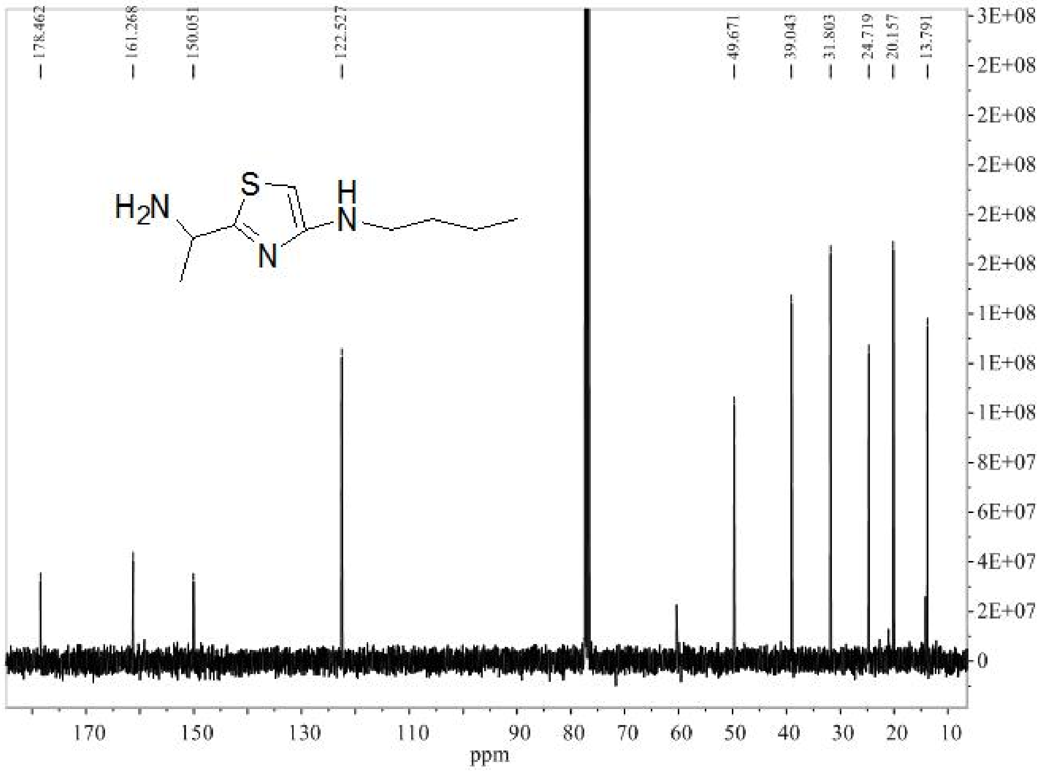
178.462
161.268
150.051
122.527
49.671
39.043
31.803
24.719
20.157
13.791
H2N
S
N
H
N
ppm

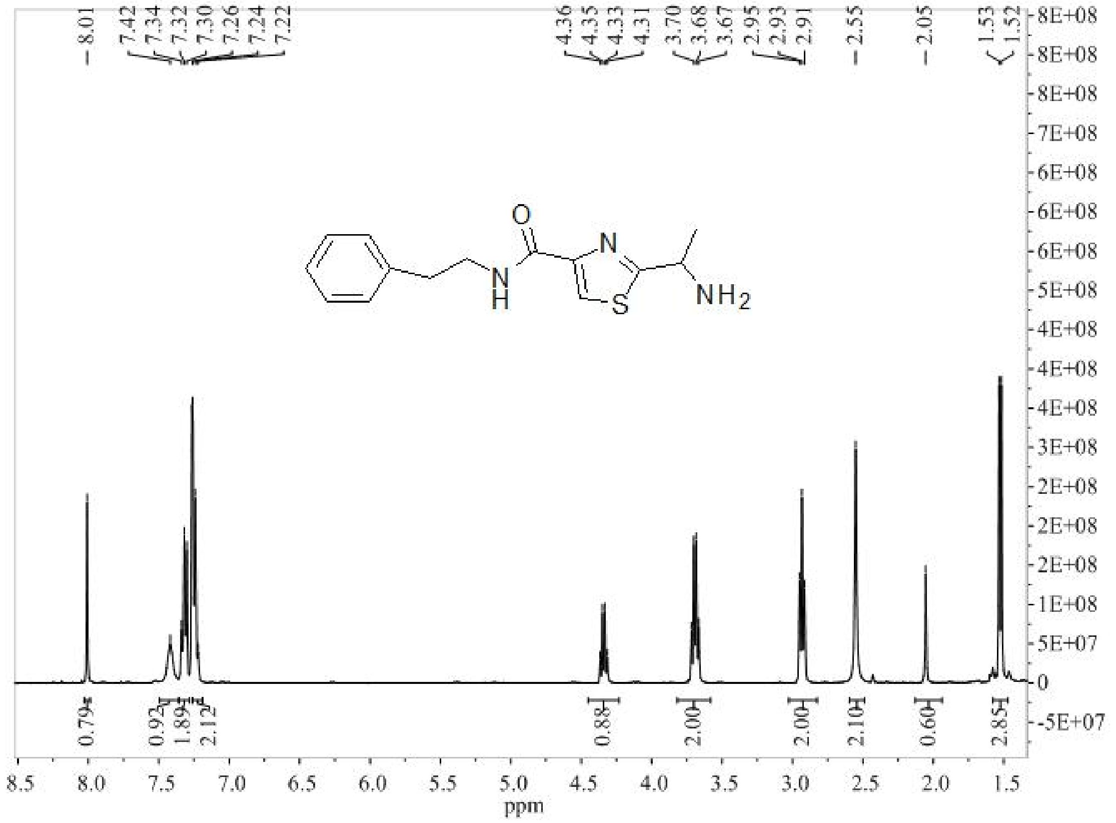
8.01
7.42
7.34
7.32
7.30
7.26
7.24
7.22
4.36
4.35
4.33
4.31
3.70
3.68
3.67
2.95
2.93
2.91
2.55
2.05
1.53
1.52
O
N
N
H
S
NH2
0.79
0.93
1.89
2.12
0.88
2.00
2.00
2.10
0.60
2.85
ppm

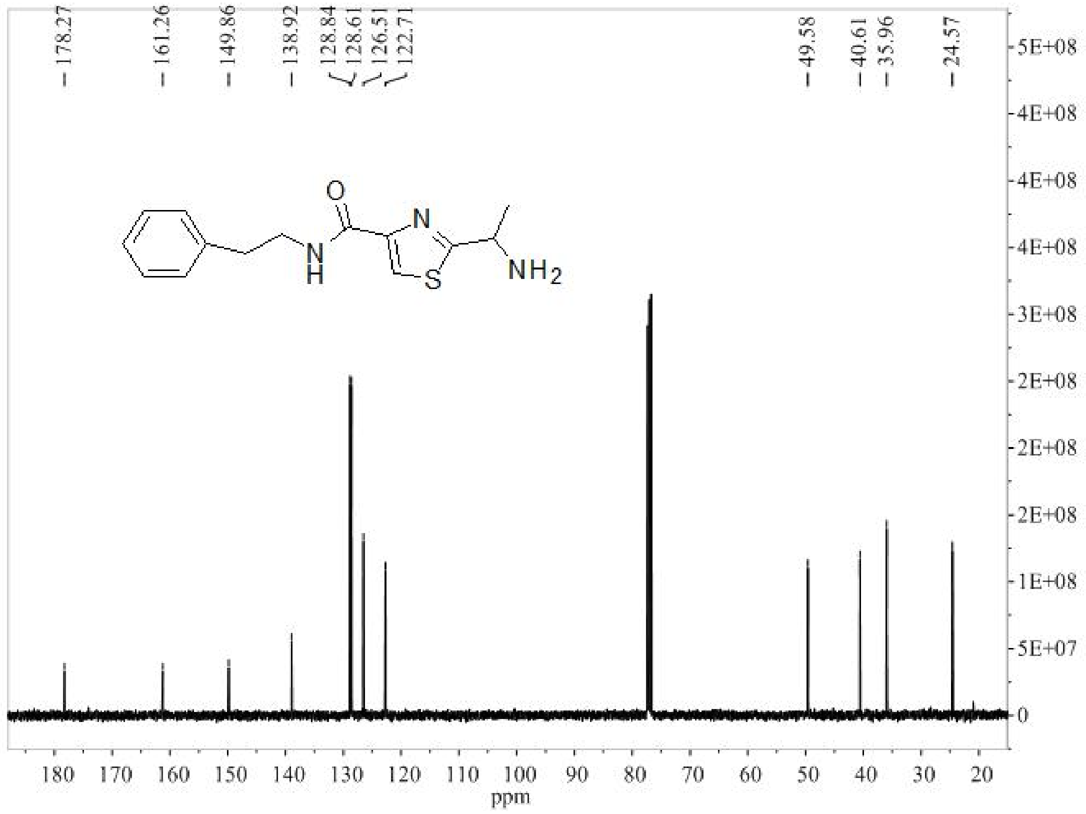
178.27
161.26
149.86
138.92
128.84
128.61
126.51
122.71
49.58
40.61
35.96
24.57
O
N
N
H
S
NH2
ppm

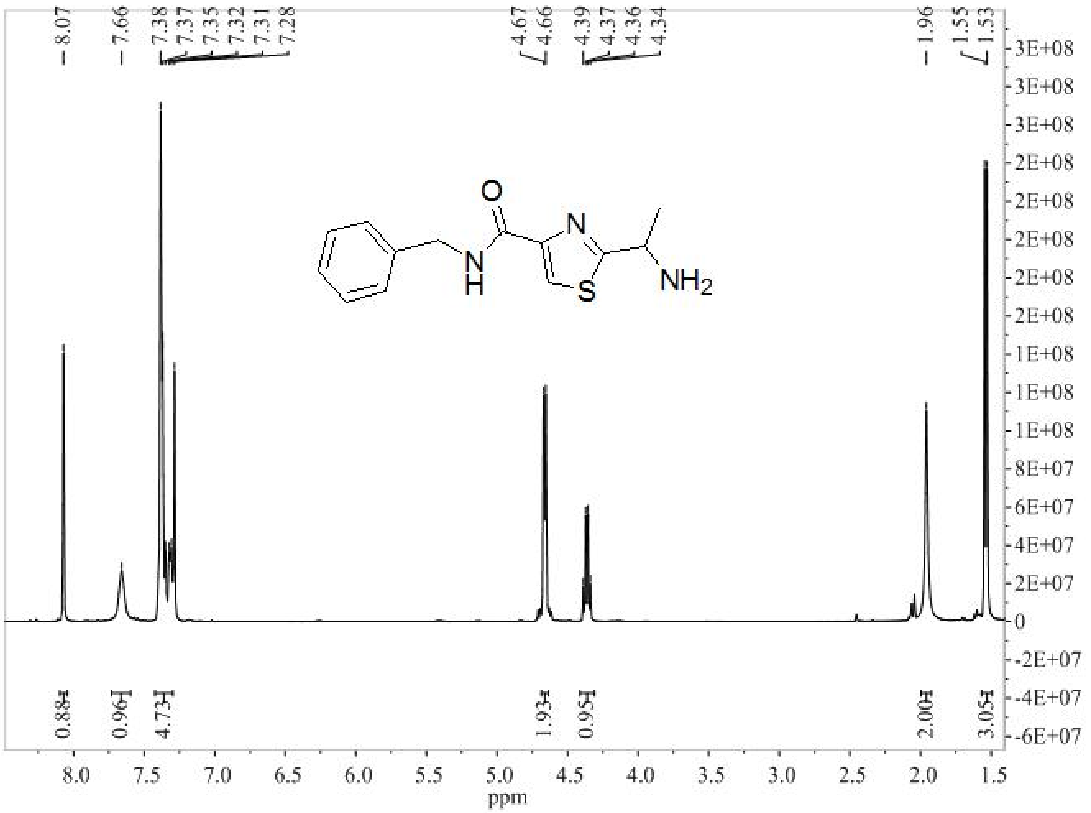
8.07
7.66
7.38
7.37
7.35
7.32
7.31
7.28
4.67
4.66
4.39
4.37
4.36
4.34
1.96
1.55
1.53
O
N
H
N
S
NH2
0.88
0.96
4.73
1.93
0.95
2.00
3.05
ppm

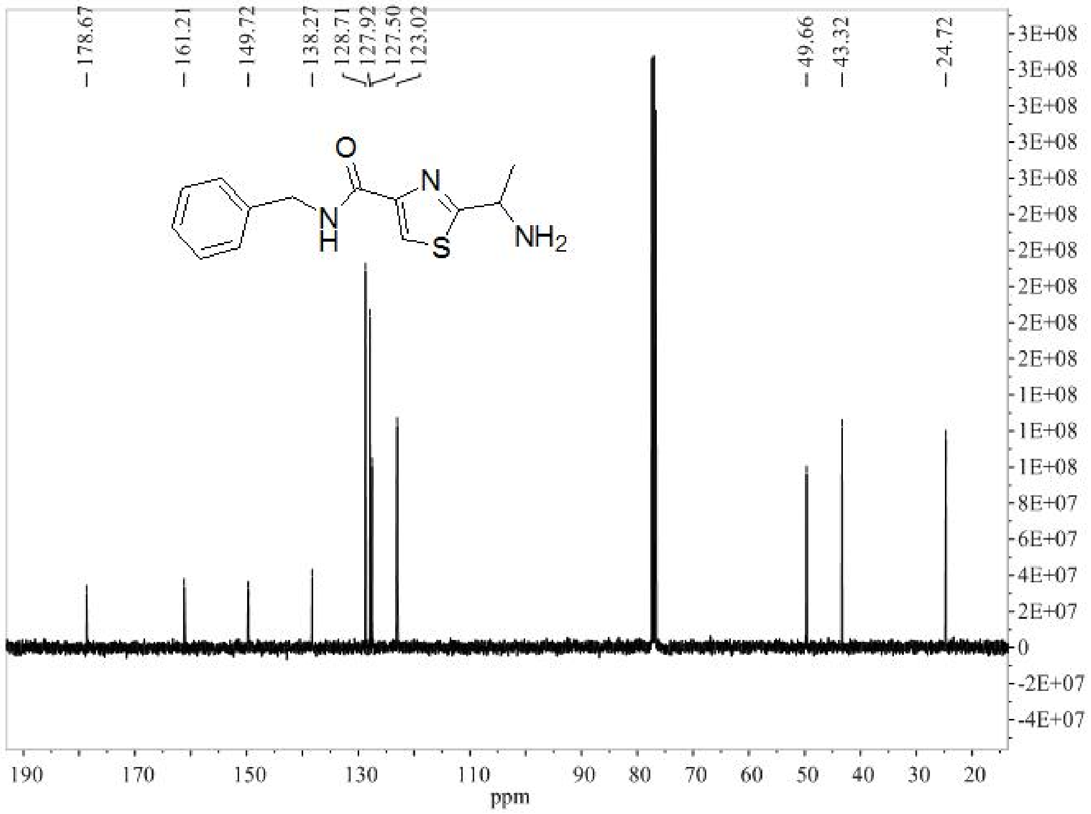
178.67
161.21
149.72
138.27
128.71
127.92
127.50
123.02
49.66
43.32
24.72
O
N
H
N
S
NH2
ppm

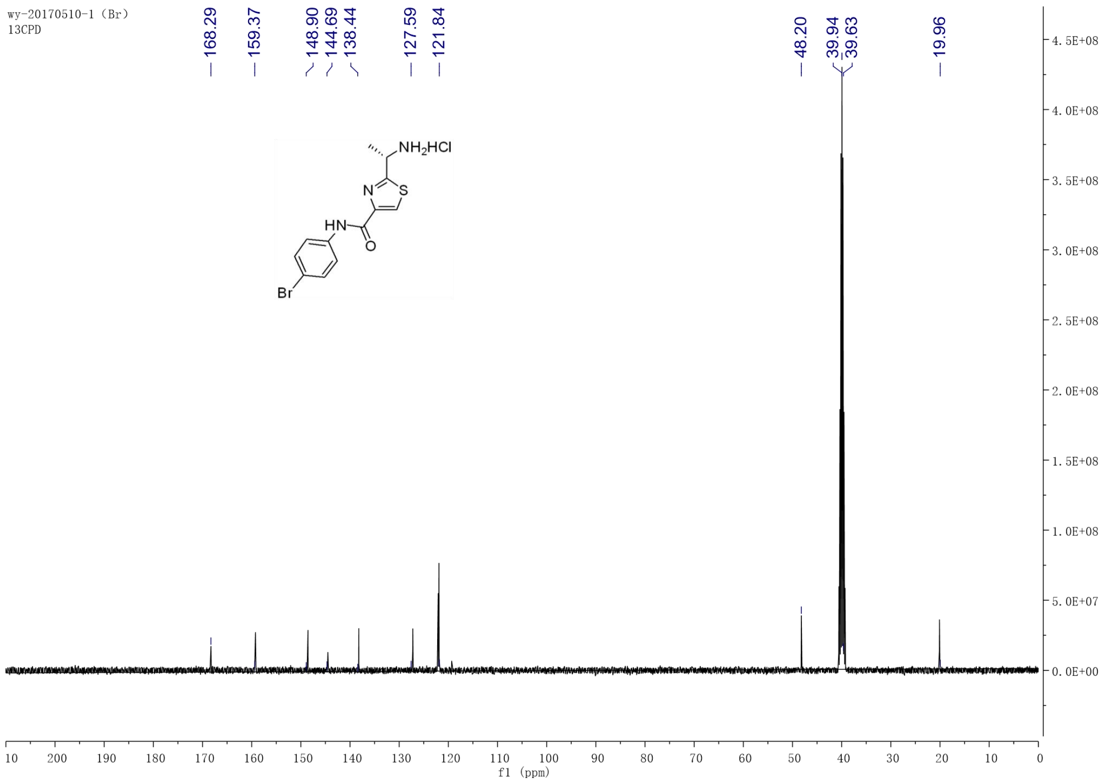
wy-20170510-1 (Br)
13CPD
168.29
159.37
148.90
144.69
138.44
127.59
121.84
48.20
39.94
39.63
19.96
NH2HCl
N
S
HN
O
Br
f1 (ppm)

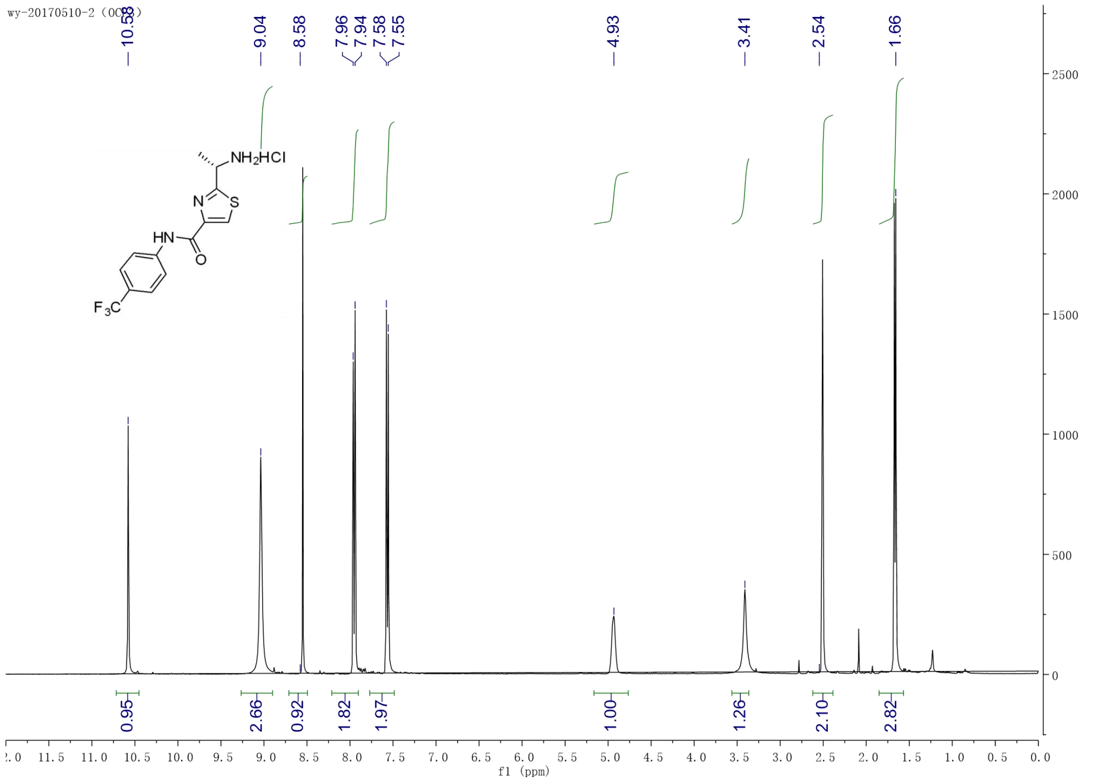
wy-20170510-2 (OCF3)
10.58
9.04
8.58
7.96
7.94
7.58
7.55
4.93
3.41
2.54
1.66
NH2HCl
N
S
HN
O
F3C
0.95
2.66
0.92
1.82
1.97
1.00
1.26
2.10
2.82
f1 (ppm)

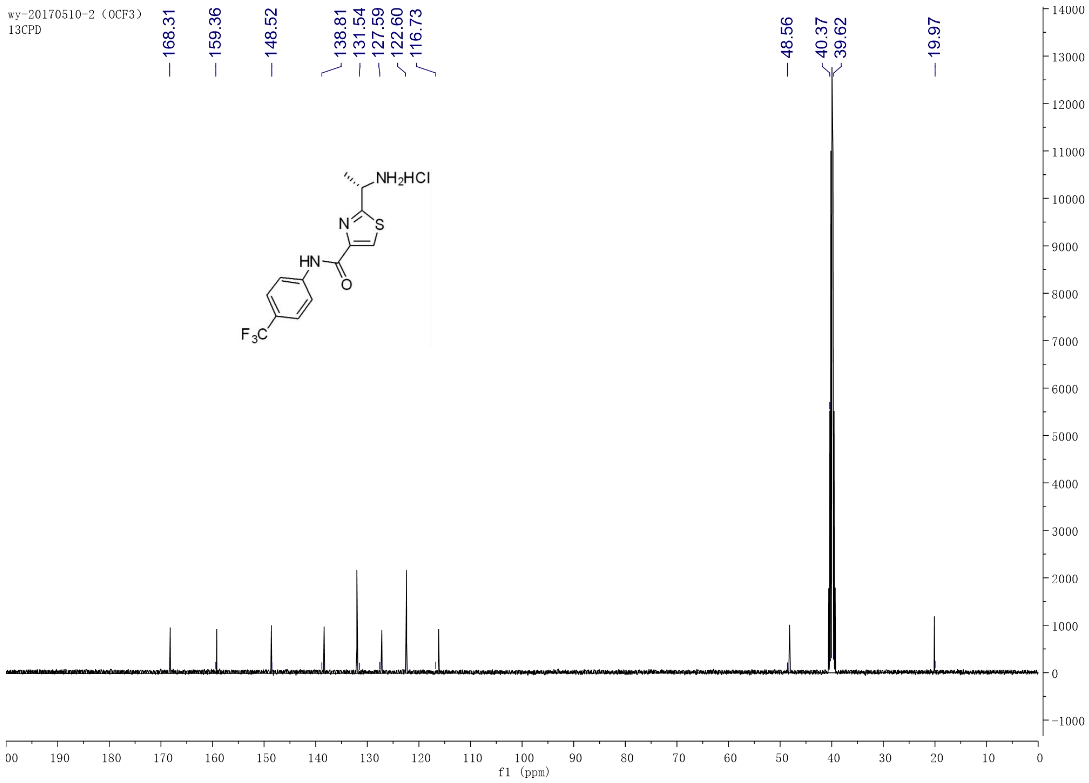

wy-20170510-2（OCF3）
13CPD
168.31
159.36
148.52
138.81
131.54
127.59
122.60
116.73
48.56
40.37
39.62
19.97
NH2HCl
N
S
HN
O
F3C
f1 (ppm)
